# Physician Practice Pattern Variations in Common Clinical Scenarios Within 5 US Metropolitan Areas

**DOI:** 10.1001/jamahealthforum.2021.4698

**Published:** 2022-01-28

**Authors:** Zirui Song, Sneha Kannan, Robert J. Gambrel, Molly Marino, Muthiah Vaduganathan, Mark A. Clapp, Jacqueline A. Seiglie, Patricia P. Bloom, Athar N. Malik, Matthew J. Resnick

**Affiliations:** 1 Department of Health Care Policy, Harvard Medical School, Boston, Massachusetts; 2 Department of Medicine, Massachusetts General Hospital, Boston; 3 Embold Health, Nashville, Tennessee; 4 Division of Cardiovascular Medicine, Department of Medicine, Brigham and Women’s Hospital, Boston, Massachusetts; 5 Department of Obstetrics & Gynecology, Massachusetts General Hospital, Boston; 6 Diabetes Unit, Massachusetts General Hospital, Boston; 7 Division of Gastroenterology, University of Michigan, Ann Arbor; 8 Department of Neurosurgery, Massachusetts General Hospital, Boston; 9 Department of Urology, Vanderbilt University Medical Center, Nashville, Tennessee

## Abstract

**Importance:**

While variations in quality of care have been described between US regions, physician-level practice pattern variations within regions remain poorly understood, notably among specialists.

**Objective:**

To examine within-area physician-level variations in decision-making in common clinical scenarios where guidelines specifying appropriateness or quality of care exist.

**Design, Setting, and Participants:**

This cross-sectional study used 2016 through 2019 data from a large nationwide network of commercial insurers, provided by Health Intelligence Company, LLC, within 5 metropolitan statistical areas (MSAs). Physician-level variations in appropriateness and quality of care were measured using 14 common clinical scenarios involving 7 specialties. The measures were constructed using public quality measure definitions, clinical guidelines, and appropriateness criteria from the clinical literature. Physician performance was calculated using a multilevel model adjusted for patient age, sex, risk score, and socioeconomic status with physician random effects. Measure reliability for each physician was calculated using the signal-to-noise approach. Within-MSA variation was calculated between physician quintiles adjusted for patient attributes, with the first quintile denoting highest quality or appropriateness and the fifth quintile reflecting the opposite. Data were analyzed March through October 2021.

**Main Outcomes and Measures:**

Fourteen measures of quality or appropriateness of care, with 2 measures each in the domains of cardiology, endocrinology, gastroenterology, pulmonology, obstetrics, orthopedics, and neurosurgery.

**Results:**

A total of 8788 physicians were included across the 5 MSAs, and about 2.5 million unique patient-physician pairs were included in the measures. Within the 5 MSAs, on average, patients in the measures were 34.7 to 40.7 years old, 49.1% to 52.3% female, had a mean risk score of 0.8 to 1.0, and more likely to have an employer-sponsored insurance plan that was either self-insured or fully insured (59.8% to 97.6%). Within MSAs, physician-level variations were qualitatively similar across measures. For example, statin therapy in patients with coronary artery disease ranged from 54.3% to 70.9% in the first quintile of cardiologists to 30.5% to 42.6% in the fifth quintile. Upper endoscopy in patients with gastroesophageal reflux disease without alarm symptoms spanned 14.6% to 16.9% in the first quintile of gastroenterologists to 28.2% to 33.8% in the fifth quintile. Among patients with new knee or hip osteoarthritis, 2.1% to 3.4% received arthroscopy in the first quintile of orthopedic surgeons, whereas 25.5% to 30.7% did in the fifth quintile. Appropriate prenatal screening among pregnant patients ranged from 82.6% to 93.6% in the first quintile of obstetricians to 30.9% to 65.7% in the fifth quintile. Within MSAs, adjusted differences between quintiles approximated unadjusted differences. Measure reliability, which can reflect consistency and reproducibility, exceeded 70.0% across nearly all measures in all MSAs.

**Conclusions and Relevance:**

In this cross-sectional study of 5 US metropolitan areas, sizeable physician-level practice variations were found across common clinical scenarios and specialties. Understanding the sources of these variations may inform efforts to improve the value of care.

## Key Points

**Question:**

To what extent do physician-level variations in the appropriateness or quality of care exist within metropolitan areas, notably among specialists?

**Findings:**

In this cross-sectional study of 8788 physicians across 7 specialties in 5 US metropolitan areas, sizeable physician-level practice pattern variations were evident across 14 common clinical scenarios where practice guidelines and clinical evidence can help discern, on average, the appropriateness or quality of clinical decisions. Variations were robust to adjustment for patient and area-level characteristics, and measure reliability was generally high.

**Meaning:**

Within-area physician-level variations in practice patterns were qualitatively similar across clinical scenarios, despite practice guidelines designed to reduce variation.

## Teaser

This cross-sectional study examines within-area physician-level variations in decision-making in common clinical scenarios where guidelines specifying appropriateness or quality of care exist.

## Introduction

While efforts to improve the value of health care have adopted a national scope, most health care decisions remain local. Patients and families typically choose among physicians and hospitals close to home. Employers and insurers contract with local physicians and hospitals for their insurance networks. Yet patients, purchasers, insurers, and health care delivery organizations face a common challenge: variations in the appropriateness or quality of care between physicians are generally unknown.

To date, variations in quality and spending have been largely described between regions, notably by the Dartmouth Atlas of Health Care.^1^ Following this seminal work, the Institute of Medicine also noted substantial variation within regions and recommended that efforts to improve the value of care focus on differences in clinical decision-making rather than geographic variation.^2,3^ Early surveys using clinical vignettes focused on primary care physicians revealed varying clinical preferences among physicians between high- and low-spending regions.^4,5,6^ Direct measurement of low-value care, also focused on primary care physicians, has found large variations between physicians, including within regions.^7,8,9,10^ However, the evidence on within-region physician-level variations in appropriateness or quality remains scant—especially for specialists whose services play an important role in determining health care spending and patient outcomes.

In this cross-sectional study, we examined within-area physician-level variations in appropriateness and quality across 14 sentinel clinical scenarios pertaining to 7 specialties. As a proof of concept, we hypothesized that clinically meaningful variations in claims data are measurable with rigor among similar patients across physicians of the same specialty within a metropolitan area. Measuring physician-level variations may inform quality improvement efforts and stimulate local competition on quality that benefits patients. It may also help patients, employers, and insurers choose physicians and design insurance benefits beyond word of mouth or historically contracted networks.^11,12,13^

## Methods

### Data Collection

We analyzed the 2016 through 2019 claims and enrollment data from a national network of insurers provided by Health Intelligence Company, LLC. We examined physicians in 5 metropolitan statistical areas (MSAs), each of which contains at least 1 urban area of 50 000 inhabitants,^14^ where included insurers had high market penetration. To protect confidentiality, all data were deidentified and HIPAA (Health Insurance Portability and Accountability Act) compliant. Moreover, we omitted MSA names and instead reported them as Southeast MSA 1, Southeast MSA 2, South Central MSA, Midwest MSA, and West MSA. Institutional review board approval was obtained from Harvard Medical School and Embold Health, and need for informed consent was waived owing to the use of deidentified data. This work followed the Strengthening the Reporting of Observational Studies in Epidemiology (STROBE) reporting guidelines for cross-sectional studies.^15^

### Measure Specification

We constructed quality measures for 14 common clinical scenarios based on current measures in the public domain, clinical guidelines from professional societies, appropriate use criteria, and established end points from the clinical literature.^16,17^ These scenarios were defined by eligible patients with certain diagnoses, specified via *International Statistical Classification of Diseases and Related Health Problems, Tenth Revision, *diagnosis codes, who received a given service, defined through the *Common Procedural Terminology* or drug codes, that reflected care of high or low appropriateness or quality. Physician performance in a measure was the share of patients whose care met the definition for appropriateness or quality, or reflected a continuous variable such as rates.

The clinical scenarios pertained to 7 specialties and included 2 scenarios each in cardiologist coronary artery disease care (stress tests and statin therapy), endocrinologist diabetes care (kidney function testing and drug therapy), gastroenterologist gastrointestinal tract care (polyp detection and appropriate endoscopy), pulmonologist chronic obstructive pulmonary disease care (drug therapy and spirometry), obstetrician prenatal and delivery care (prenatal screening and cesarean delivery), orthopedist joint care (preoperative care and arthroscopy), and orthopedic surgeon or neurosurgeon spine care (spinal fusion and physical therapy). Within each measure, patients were attributed to the physician who was plausibly most directly accountable for the care in that measure. Attribution to proceduralists was based on a triggering event (eg, births or surgery). Attribution to nonprocedural specialists was through the plurality of clinical encounters in that specialty over a 3-year period. Rare ties in attribution were broken by attributing to the most recent specialist the patient saw.

### Measure Validity

The measures were designed to assess variations in physician-level decision-making in standardized clinical scenarios where, on average, more appropriate or higher-quality care could be plausibly distinguished from less appropriate or lower-quality care among similar patients in similar situations across physicians of the same specialty. Deriving measures of appropriateness or quality from claims data is challenging owing to potentially unobserved aspects of the patient or clinician that may affect the quality of clinical decisions.^18,19^ Recognizing these limitations, we focused on the narrow set of 14 measures for which guidelines and prior literature provide clear distinctions on appropriateness and quality. The guidelines and evidence behind each measure are provided in eMethods 1 in the Supplement.

To support measure validity, 9 of the 14 measures adhered to measure specifications from the National Quality Forum, the National Committee for Quality Assurance’s Healthcare Effectiveness Data and Information Set, the Agency for Healthcare Research and Quality (AHRQ), or the Centers for Medicare & Medicaid Services.^20,21^ These specifications have gone through rigorous review and are commonly used by Medicare and commercial insurers. References to these measures in the public domain are provided in eFigures 1 through 7 in the Supplement.

For the 5 measures not currently in the public domain, we used detailed claims analyses to similarly restrict to patients in a specific clinical situation where rigorous guidelines exist. For example, in the measure of arthroscopic surgery for new hip or knee osteoarthritis, which has been demonstrated in multiple randomized clinical trials to lack benefit and is not recommended in such patients in guidelines,^22,23,24^ we identified patients without previous evidence of knee or hip pain who had incident osteoarthritis without meniscal abnormality. Within these patients, we measured rates of arthroscopy performed by their orthopedic surgeons in the subsequent year. The specifications for these measures are similarly provided in eFigures 1 through 7 in the Supplement.

To further examine and improve measure validity, 3 of the investigators (Z.S., S.K., M.J.R.) conducted a series of internal validation exercises by comparing the claims-based measure outputs and claims-based physician attribution to those obtained from a random subset of patient electronic medical record data. In these exercises, we selected a random subsample of patients in a given measure and constructed the measure output de novo by hand using medical record data. In essentially all cases, we found concordance between the 2 methods in physician attribution and measure output.

### Measure Reliability

Measure reliability for each physician was calculated using the signal-to-noise approach, which examines the ratio of the variation between physicians and total variation within a measure—the latter comprising between-physician and within-physician variation. A linear random effects model was fit to estimate the clinical event of interest, with the physician as the independent variable alongside a random intercept.^25^ The covariance parameter estimate serves as the between-physician variation; the within-physician variation was calculated by the sum of the squared residuals from the model, divided by N(N − 1). Standards for acceptable reliability (a reflection of consistency and reproducibility) depend on a measure’s specification and its intended use.^26,27^ Consistent with AHRQ methodology, we considered a reliability greater than 0.7 to be high and 0.4 to 0.7 to be acceptable.^24^ To improve reliability, physicians with fewer than 10 attributed patients in a measure were excluded. We reported the average number of patients per physician in each measure.

### Statistical Analyses

In unadjusted analyses, we calculated mean physician performance with 95% CIs reflecting uncertainty around the mean after adjustment for case volume. These reveal the observed variation in a transparent manner but do not account for patient factors or practice patterns that may explain between-physician differences.

In adjusted analyses, we estimated physician performance using a multilevel model that adjusted for patient age, sex, diagnostic cost group risk score, index of socioeconomic status, and physician random effects with observations clustered within physicians. The diagnostic cost group risk score is driven by clinical diagnoses and is commonly used by insurers in risk adjustment. The socioeconomic status index was constructed using 7 US Census variables on education, income, housing, poverty rate, and unemployment rate from the patient’s zip code of residence following AHRQ methods (eMethods 1 in the Supplement).^28^ Without patient-level data on social determinants of health in claims, this index helps address, though does not resolve, concerns about claims-based quality measurement noted in the Institute of Medicine report,^3^ such as differences in health care access, patient preferences, and other unobserved factors.

These “shrinkage” estimates from the random effects model typically reduce variations in performance between physicians, as outliers with less precise performance are pulled toward the mean performance in an area. Therefore, they present a more conservative description of within-MSA variation. For transparency, we present them side by side with the nonshrinkage estimates to show the contribution of the random effects model.

Physician performance in each measure and MSA was shown graphically with physicians ordered by mean performance, accompanied by 95% CIs. We then compared mean physician-level performance between quintiles of physicians in each measure, comparing quintile 1 (more appropriate or higher quality) to those in each subsequent quintile. We estimated between-quintile differences at the patient level with patients assigned to physicians and quintiles based on the random effects model. The coefficient of interest captured the difference in mean performance between physicians in quintile 1 and those in a subsequent quintile of a measure, adjusted for patient age, sex, risk score, and index of socioeconomic status derived from the 7 US Census variables based on the patient’s zip code of residence, according to the methodology of AHRQ.^28^ Standard errors were clustered by physician. Owing to the large number of comparisons across the 14 measures and 5 MSAs, with each comparison of equal importance relative to the others (in other words, we did not have a primary outcome), we did not conduct individual statistical tests for each difference in mean performance. We used 95% CIs to convey the uncertainty around mean differences in performance. Sensitivity analyses tested alternative specifications. For additional details, please see eMethods 2 in the Supplement.

Finally, we conducted 2 exploratory analyses. First, we explored the correlation between average physician performance across the 2 measures in a specialty and average spending on the corresponding episode of care within that specialty attributable to the physician (eg, cardiologist performance on coronary artery disease measures and spending on coronary artery disease–related claims). Spending reflected insurer-standardized negotiated prices using the HealthPartners standardized costs of care measures.^29^ This was motivated by the question of whether physicians whose episode-level spending was lower on average tended to be physicians whose patients also received more clinically appropriate care in the situations we measured. Second, in 1 MSA where we had data on physician organizational affiliations, we explored physician-level variations within organizations and across organizations. Analyses were performed using R software, version 4.0.5 (R Foundation).

## Results

### Study Population

A total of 8788 physicians across the 7 specialties and 5 MSAs, comprising about two-thirds of all specialists in these MSAs, were included in the 14 measures. The characteristics of patients are shown in Table 1, with the number of attributed patients per physician by measure displayed in eMethods 3 in the Supplement. Mean physician performance with 95% CIs for each measure are plotted in eFigures 1 through 7 in the Supplement, with the average measure reliability across specialists in a measure and the proportion of specialists with measure reliability above 70.0% reported for each MSA. Several example measures from the South Central MSA are shown in the Figure.

**Table 1.** Characteristics of the Study Population

Characteristics	No. of physicians measured in each MSA (% of physicians in the specialty)^a^				
	Southeast 1	Southeast 2	South Central	Midwest	West
Physicians by specialty					
Cardiology	300 (81)	318 (77)	366 (78)	561 (70)	174 (70)
Endocrinology	69 (91)	75 (85)	113 (93)	203 (88)	71 (85)
Gastroenterology	167 (91)	203 (93)	284 (92)	421 (90)	181 (93)
Obstetrics	277 (60)	311 (64)	608 (71)	809 (61)	360 (73)
Orthopedics (joint)	133 (53)	169 (48)	288 (54)	388 (55)	163 (44)
Orthopedic surgery/neurosurgery (spine)	107 (34)	157 (36)	287 (46)	384 (45)	79 (17)
Pulmonology	107 (72)	141 (74)	149 (71)	254 (70)	111 (61)
Patient population					
Age, mean (SD), y	39.1 (18.1)	40.7 (18.5)	34.7 (18.2)	36.4 (18.6)	36.2 (18.3)
Sex, %					
Female	51.2	52.3	49.1	50.4	49.2
Male	48.8	47.7	50.9	49.6	50.8
Risk score^b^	0.9	1.0	0.9	0.8	0.8
Plan type, %					
PPO	65.7	72.5	98.8	91.8	99.4
HMO/POS	33.7	27.0	0	8.0	0
Other	0.6	0.6	1.2	0.2	0.6
Employer type, %^c^					
Self-insured	36.7	35.2	64.4	58.8	NA
Fully insured	23.1	26.5	33.2	32.5	NA
Individual^d^	40.2	38.3	2.4	8.7	NA

Abbreviations: HMO, health maintenance organization; MSA, metropolitan statistical area; NA, not available; POS, point of service; PPO, preferred provider organization.

^a^ Physicians were eligible for a measure if they met the threshold for a minimum number of patients (n = 10) who contributed to a measure. Any physician who was eligible for at least 1 measure within a specialty in a given MSA was included.

^b^ The risk score is a measure of expected spending derived using age, sex, and clinical diagnoses. It is commonly used for risk adjustment. The Southeast and South Central markets used the US Department of Health and Human Services–Hierarchical Condition Categories risk adjustment model under the Affordable Care Act. The Midwest and West markets used the diagnostic cost group risk score commonly used by private insurers. Both scores have a population average of about 1, with higher scores indicating higher expected spending.

^c^ In the West MSA, employer type was not available from the data vendor owing to confidentiality.

^d^ Individual refers to nongroup insurance plans.

**Figure.**
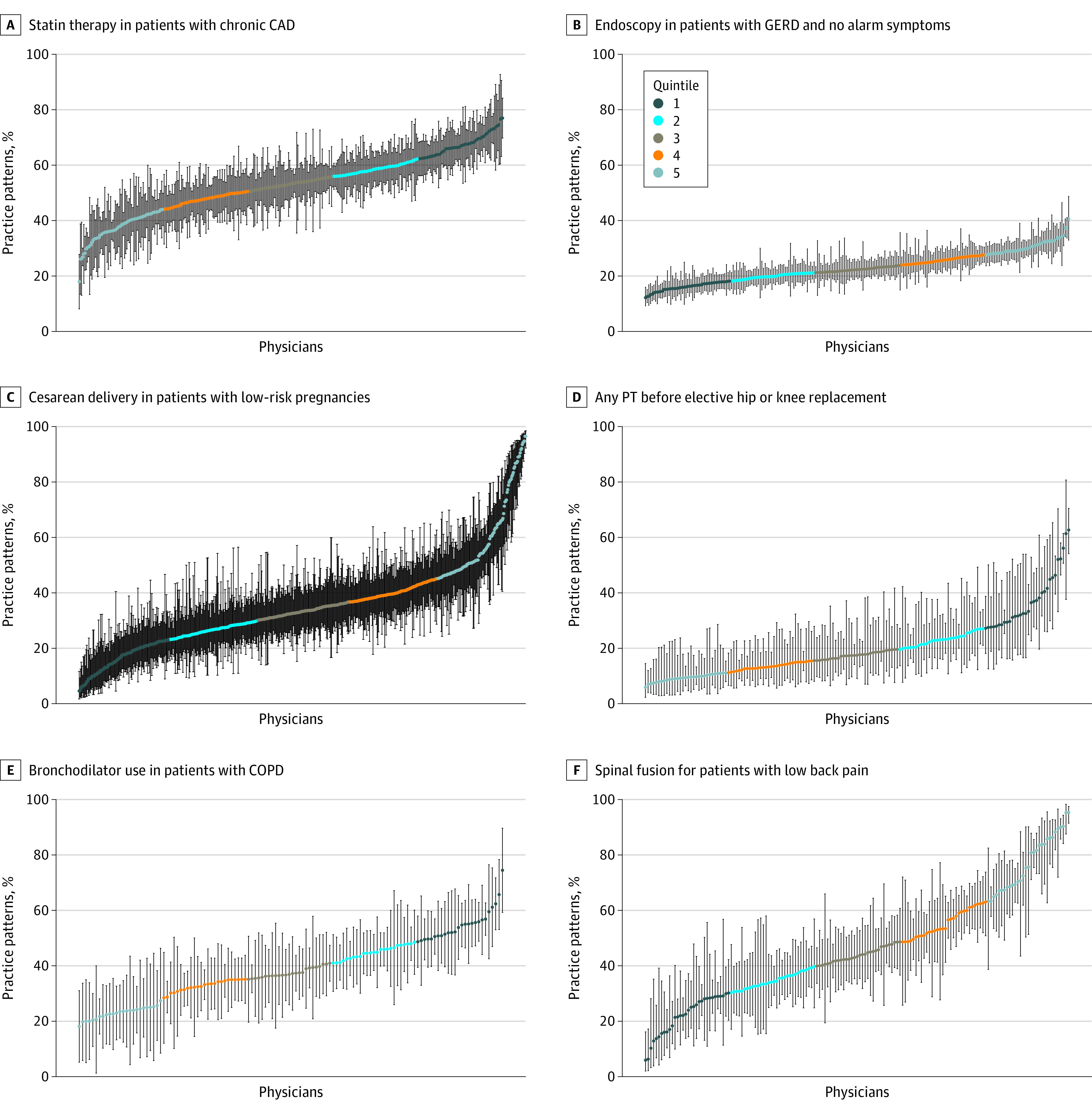
Physician-Level Variations in Practice Patterns Across 6 Example Clinical Scenarios in the South Central Metropolitan Statistical Area Example measures are shown for statin therapy in patients with chronic coronary artery disease (CAD) (A), endoscopy in patients with gastroesophageal reflux disease (GERD) and no alarm symptoms (B), caesarean delivery in patients with low-risk pregnancies (C), any physical therapy (PT) prior to elective hip or knee replacement (D), use of bronchodilator in patients with chronic obstructive pulmonary disease (COPD) (E), and spinal fusion for patients with low back pain (F). Each physician is denoted by a data point and vertical 95% CI. Quintile 1 represents, on average, more appropriate care, and quintile 5 denotes less appropriate care on average.

### Medical Specialists

Physician decision-making varied substantially across MSAs (Table 2). For example, among patients with stable chronic coronary artery disease, their cardiologists varied in the rates of stress testing from 5.4 to 7.4 tests per 100 patient-years in the first quintile (on average more appropriate) to 21.7 to 37.2 tests per 100 patient-years in the fifth quintile (on average less appropriate) within the MSAs. Physician-level measure reliability averaged 72.2% to 87.1% across the MSAs (eFigure 1 in the Supplement). Analogously, the proportion of patients with coronary artery disease being treated with a statin ranged from 54.3% to 70.9% in the first quintile to 30.5% to 42.6% in the fifth quintile of cardiologists, with the average measure reliability ranging from 79.1% to 88.3% (eFigure 1 in the Supplement).

**Table 2.** Physician-Level Variations in Practice Patterns by Medical Specialty^a^

Specialty	Quintile 1	Quintile 2		Quintile 3		Quintile 4		Quintile 5	
	Mean [reference]	Mean	Adjusted difference (95% CI)	Mean	Adjusted difference (95% CI)	Mean	Adjusted difference (95% CI)	Mean	Adjusted difference (95% CI)
**Cardiology**									
No. of stress tests in patients with stable chronic CAD per 100 patient-years^b^									
Southeast MSA 1	6.2	10.2	2.4 (1.8 to 3.1)	14.1	4.7 (4.1 to 5.4)	15.3	7.5 (6.8 to 8.1)	21.7	15.0 (13.2 to 16.9)
Southeast MSA 2	5.4	11.4	3.2 (2.5 to 3.8)	13.6	6.2 (5.6 to 6.8)	17.8	9.4 (8.7 to 10.1)	24.1	17.4 (15.4 to 19.3)
South Central MSA	6.6	11.9	5.0 (4.2 to 5.9)	17.1	8.9 (8.1 to 9.7)	22.5	13.2 (12.2 to 14.2)	37.2	25.4 (23.3 to 27.6)
Midwest MSA	7.4	12.6	3.5 (3.0 to 4.0)	15.1	6.7 (6.1 to 7.2)	19.9	10.5 (9.9 to 11.1)	30.9	21.8 (19.4 to 24.3)
West MSA	6.7	10.2	3.3 (2.5 to 4.0)	13.4	6.0 (5.2 to 6.8)	18.1	9.9 (9.1 to 10.8)	24.8	17.2 (14.8 to 19.6)
Statin therapy in patients with chronic CAD, %									
Southeast MSA 1	54.3	47.6	−7.4 (−9.0 to −5.8)	43.2	−12.0 (−13.5 to −10.4)	38.4	−17.0 (−18.6 to −15.3)	30.5	−24.4 (−26.3 to −22.5)
Southeast MSA 2	57.2	48.9	−8.8 (−10.4 to −7.3)	45.0	−13.4 (−14.8 to −11.9)	40.2	−18.5 (−19.9 to −17.0)	31.9	−26.9 (−29.1 to −24.7)
South Central MSA	67.7	58.9	−9.3 (−10.4 to −8.2)	54.3	−14.6 (−15.7 to −13.5)	48.0	−21.1 (−22.2 to −20.0)	36.7	−32.8 (−35.1 to −30.5)
Midwest MSA	68.5	61.3	−6.9 (−7.6 to −6.2)	56.9	−11.4 (−12.2 to −10.7)	51.4	−17.0 (−17.8 to −16.2)	40.6	−27.9 (−29.6 to −26.2)
West MSA	70.9	64.2	−7.3 (−9.1 to −5.6)	60.5	−12.4 (−14.0 to −10.8)	55.0	−17.6 (−19.4 to −15.8)	42.6	−30.5 (−33.5 to −27.6)
**Endocrinology**									
Kidney function test in patients with diabetes, %									
Southeast MSA 1	85.9	83.1	−3.0 (−4.4 to −1.5)	78.1	−8.2 (−9.8 to −6.5)	72.7	−13.7 (−16.0 to −11.5)	58.9	−27.7 (−32.6 to −22.9)
Southeast MSA 2	88.0	80.3	−7.9 (−10.5 to −5.3)	72.5	−16.0 (−18.0 to −14.1)	64.7	−23.7 (−26.0 to −21.4)	51.2	−37.0 (−43.9 to −30.1)
South Central MSA	93.2	90.2	−3.3 (−4.2 to −2.3)	86.8	−6.8 (−7.9 to −5.7)	82.5	−11.2 (−12.8 to −9.5)	69.8	−24.1 (−27.1 to −21.0)
Midwest MSA	85.8	80.2	−5.5 (−6.6 to −4.4)	74.0	−11.9 (−13.1 to −10.7)	67.3	−18.6 (−20.3 to −16.8)	49.6	−35.9 (−39.4 to −32.4)
West MSA	84.9	76.6	−8.4 (−11.4 to −5.5)	63.1	−22.0 (−25.8 to −18.2)	37.4	−48.3 (−51.8 to −44.9)	22.6	−62.1 (−66.5 to −57.7)
Oral antidiabetic drug in patients with diabetes, %^c^									
Southeast MSA 1	55.0	49.2	−5.3 (−7.3 to −3.3)	46.1	−8.5 (−10.5 to −6.5)	44.1	−11.4 (−13.5 to −9.2)	32.8	−23.2 (−27.6 to −18.9)
Southeast MSA 2	51.1	46.1	−4.7 (−6.6 to −2.7)	43.1	−8.8 (−10.4 to −7.1)	39.0	−13.3 (−15.3 to −11.2)	29.8	−20.8 (−28.2 to −13.3)
South Central MSA	57.6	52.9	−5.3 (−7.1 to −3.5)	47.4	−10.3 (−12.1 to −8.5)	44.1	−15.4 (−17.4 to −13.3)	33.4	−26.8 (−29.9 to −23.7)
Midwest MSA	55.4	49.7	−5.9 (−7.2 to −4.6)	45.6	−9.7 (−11.1 to −8.2)	41.7	−14.3 (−15.7 to −12.9)	30.9	−24.6 (−28.6 to −20.5)
West MSA	59.4	53.4	−5.3 (−7.8 to −2.7)	47.3	−13.6 (−15.9 to −11.4)	42.1	−17.9 (−20.1 to −15.7)	28.9	−31.2 (−36.8 to −25.5)
**Gastroenterology**									
Polyp detected on screening colonoscopy, %									
Southeast MSA 1	76.9	64.2	−12.7 (−15.7 to −9.7)	56.5	−20.6 (−23.7 to −17.5)	46.1	−30.8 (−33.7 to −27.9)	34.6	−42.6 (−46.4 to −38.7)
Southeast MSA 2	77.1	62.6	−14.3 (−16.6 to −12.1)	55.5	−21.8 (−24.1 to −19.6)	44.6	−32.7 (−35.3 to −30.1)	30.8	−46.7 (−50.9 to −42.4)
South Central MSA	84.3	72.8	−11.5 (−13.3 to −9.7)	64.7	−19.7 (−21.4 to −17.9)	56.8	−27.6 (−29.4 to −25.7)	45.1	−39.4 (−41.7 to −37.2)
Midwest MSA	81.9	66.5	−15.7 (−18.4 to −12.9)	58.9	−23.0 (−25.7 to −20.3)	52.0	−30.1 (−32.8 to −27.5)	38.5	−43.4 (−46.6 to −40.3)
West MSA	77.3	69.4	−8.2 (−10.0 to −6.4)	64.1	−13.7 (−15.5 to −11.9)	56.9	−20.9 (−22.6 to −19.1)	45.8	−31.9 (−35.0 to −28.9)
Endoscopy in patients with GERD and no alarm symptoms, %^d^									
Southeast MSA 1	16.7	21.3	4.6 (3.5 to 5.8)	24.4	7.8 (6.7 to 8.8)	27.7	11.0 (9.9 to 12.2)	32.0	15.4 (13.7 to 17.1)
Southeast MSA 2	16.9	20.4	3.5 (2.4 to 4.6)	22.7	5.9 (4.9 to 7.0)	25.3	8.5 (7.5 to 9.6)	29.2	12.3 (10.9 to 13.8)
South Central MSA	15.6	20.0	4.4 (3.8 to 5.0)	22.6	7.1 (6.5 to 7.8)	26.2	11.0 (10.3 to 11.7)	31.1	15.8 (14.8 to 16.8)
Midwest MSA	14.6	20.1	5.5 (4.6 to 6.4)	23.3	8.7 (7.8 to 9.6)	26.8	12.2 (11.3 to 13.1)	33.8	19.4 (17.6 to 21.3)
West MSA	15.5	19.2	3.6 (2.7 to 4.5)	21.9	6.4 (5.5 to 7.3)	24.3	8.9 (8.1 to 9.8)	28.2	12.8 (11.6 to 13.9)
**Pulmonology**									
Bronchodilator use in patients with COPD, %^e^									
Southeast MSA 1	43.7	38.4	−8.0 (−10.6 to −5.3)	35.0	−12.2 (−14.4 to −10.0)	32.4	−15.7 (−18.0 to −13.4)	25.9	−22.9 (−26.2 to −19.6)
Southeast MSA 2	45.1	39.8	−8.9 (−11.1 to −6.8)	33.5	−14.9 (−17.1 to −12.7)	29.7	−19.4 (−21.8 to −17.1)	24.9	−25.3 (−32.0 to −18.6)
South Central MSA	51.9	42.8	−11.6 (−14.1 to −9.0)	37.2	−19.6 (−22.3 to −16.9)	31.7	−25.2 (−27.8 to −22.5)	19.8	−38.8 (−41.6 to −36.1)
Midwest MSA	48.5	39.7	−10.6 (−13.1 to −8.1)	34.6	−17.2 (−19.6 to −14.8)	28.5	−24.1 (−26.6 to −21.5)	21.6	−33.7 (−36.7 to −30.7)
West MSA	52.2	43.2	−10.8 (−13.7 to −7.8)	39.7	−16.6 (−19.5 to −13.7)	32.9	−25.0 (−28.4 to −21.5)	24.7	−37.1 (−41.1 to −33.1)
Spirometry use in patients with COPD, %									
Southeast MSA 1	90.0	80.7	−9.3 (−11.5 to −7.2)	72.5	−18.0 (−20.1 to −15.9)	65.1	−25.7 (−28.4 to −23.0)	49.3	−41.5 (−45.0 to −37.9)
Southeast MSA 2	87.7	76.3	−11.9 (−15.0 to −8.8)	67.2	−21.1 (−23.7 to −18.4)	58.7	−29.4 (−32.1 to −26.8)	41.4	−46.7 (−54.8 to −38.7)
South Central MSA	98.7	92.0	−6.9 (−8.1 to −5.7)	83.2	−15.4 (−16.4 to −14.5)	73.3	−25.9 (−28.4 to −23.5)	59.0	−40.1 (−42.7 to −37.5)
Midwest MSA	90.3	78.3	−12.4 (−14.4 to −10.4)	66.4	−24.1 (−25.7 to −22.4)	58.3	−32.5 (−34.1 to −30.8)	40.1	−50.3 (−53.8 to −46.7)
West MSA	92.5	81.0	−11.6 (−14.6 to −8.7)	68.6	−24.2 (−28.0 to −20.3)	54.9	−38.2 (−41.7 to −34.6)	33.8	−58.9 (−67.0 to −50.9)

Abbreviations: CAD, coronary artery disease; COPD, chronic obstructive pulmonary disease; GERD, gastroesophageal reflux disease; MSA, metropolitan statistical area.

^a^ Measure specifications and supporting clinical guidelines are provided in eMethods 1 in the Supplement. Means represent unadjusted averages of performance by quintile of physicians. Adjusted differences between quintile 1 [reference] and quintiles 2 through 5, respectively, are derived from the statistical model adjusted for patient risk and socioeconomic variables, with standard errors clustered by physician.

^b^ Stress tests include exercise and pharmacologic stress tests.

^c^ Oral antidiabetic agents include metformin, sulfonylureas, thiazolidinediones, biguanides, bile acid agents, newer agents such as the sodium-glucose cotransporter-2 inhibitors, and others.

^d^ Alarm symptoms include vomiting, weight loss, anemia, dysphagia, and dyspepsia.

^e^ Bronchodilators include anticholinergic agents, β_2_ agonists, methylxanthines, and related agents.

Measures of endocrinologists, gastroenterologists, and pulmonologists exhibited similar variations within each MSA. However, the extent of within-MSA physician variation differed across MSAs. For example, annual kidney function testing in patients with diabetes ranged from 84.9% (quintile 1) to 22.6% (quintile 5) among endocrinologists in the West MSA, whereas it ranged from 93.2% (quintile 1) to 69.8% (quintile 5) in the South Central MSA. The average measure reliability similarly exceeded the AHRQ threshold of 70.0% for all measures and MSAs (eFigures 2-4 in the Supplement). All adjusted differences between quintiles closely approximated unadjusted differences (Table 2).

### Surgical Specialists

Among obstetricians, the proportion of pregnant patients who received appropriate prenatal screening varied from 82.6% to 93.6% (quintile 1) to 30.9% to 65.7% (quintile 5) within MSAs, and the proportion of low-risk pregnancies with cesarean delivery (on average less appropriate) ranged from 5.0% to 17.3% (quintile 1) to 51.0% to 61.5% (quintile 5) within MSAs. Average measure reliability ranged from 71.1% to 91.8% across the MSAs (eFigure 5 in the Supplement).

At least 1 session of physical therapy prior to elective hip or knee replacement (on average clinically indicated) was received by 19.1% to 64.8% of patients among orthopedists in quintile 1 compared with 3.9% to 16.6% in quintile 5, with measure reliability averaging 62.5% in the Southeast MSA and exceeding 80.0% elsewhere. The proportion of patients with new hip or knee osteoarthritis who underwent arthroscopic surgery (on average not indicated) ranged from 2.1% to 3.4% among orthopedists in quintile 1 to 25.5% to 30.7% among those in quintile 5, with average measure reliability also exceeding 75.0% in all MSAs (eFigure 6 in the Supplement).

Among patients with low back pain, the share who received spinal fusion (on average not indicated) ranged from 5.6% to 22.5% among spine surgeons in quintile 1 to 57.3% to 79.2% among those in quintile 5. Measure reliability averaged 85.0% or higher in all MSAs. The proportion of patients with cervical spine pain who received any physical therapy was 51.4% to 65.4% in quintile 1 compared with 5.7% to 29.6% in quintile 5 of surgeons. Average measure reliability similarly exceeded 70.0% in all MSAs (eFigure 7 in the Supplement). Across measures and MSAs, adjusted differences between quintiles closely approximated unadjusted differences (Table 3).

**Table 3.** Physician-Level Variations in Practice Patterns by Surgical Specialty^a^

Specialty	Quintile 1	Quintile 2		Quintile 3		Quintile 4		Quintile 5	
	Mean [reference]	Mean	Adjusted difference (95% CI)	Mean	Adjusted difference (95% CI)	Mean	Adjusted difference (95% CI)	Mean	Adjusted difference (95% CI)
**Obstetrics**									
Appropriate prenatal screening in pregnant patients, %^b^									
Southeast MSA 1	82.6	71.2	−11.1 (−13.6 to −8.6)	61.9	−20.1 (−22.6 to −17.6)	51.4	−30.5 (−33.1 to −27.9)	30.9	−51.2 (−57.2 to −45.2)
Southeast MSA 2	86.0	76.5	−9.6 (−11.6 to −7.7)	70.1	−15.8 (−17.6 to −14.0)	62.8	−23.4 (−25.5 to −21.3)	51.6	−33.8 (−37.8 to −29.9)
South Central MSA	89.5	81.9	−7.6 (−8.5 to −6.8)	76.1	−13.7 (−14.5 to −12.8)	68.6	−21.4 (−22.5 to −20.3)	52.3	−37.7 (−40.2 to −35.2)
Midwest MSA	93.6	88.6	−4.9 (−5.8 to −4.1)	82.7	−10.6 (−11.6 to −9.6)	75.9	−17.2 (−18.1 to −16.3)	65.7	−27.7 (−31.0 to −24.4)
West MSA	90.6	85.2	−5.3 (−6.3 to −4.3)	80.8	−9.6 (−10.7 to −8.6)	75.9	−14.6 (−15.8 to −13.4)	58.2	−32.2 (−37.7 to −26.6)
Cesarean delivery in patients with low-risk pregnancies, %									
Southeast MSA 1	15.7	29.4	13.8 (10.6 to 17.1)	37.1	21.5 (18.3 to 24.7)	44.6	28.9 (25.6 to 32.2)	61.5	45.5 (40.2 to 50.8)
Southeast MSA 2	17.3	27.8	10.3 (7.6 to 13.1)	34.3	16.9 (14.2 to 19.6)	40.4	23.2 (20.4 to 26.0)	53.8	36.6 (32.3 to 40.9)
South Central MSA	16.5	26.6	10.3 (9.0 to 11.6)	33.0	17.0 (15.7 to 18.2)	40.0	24.1 (22.8 to 25.4)	59.1	43.3 (39.7 to 46.9)
Midwest MSA	10.4	21.3	11.1 (9.9 to 12.2)	28.3	18.0 (16.9 to 19.2)	34.9	24.5 (23.4 to 25.7)	51.0	40.6 (37.9 to 43.3)
West MSA	5.0	19.1	13.7 (11.9 to 15.4)	26.7	21.6 (20.0 to 23.3)	35.5	30.1 (28.4 to 31.8)	56.8	51.7 (45.8 to 57.6)
**Orthopedics (joint)^c^**									
Any physical therapy prior to elective hip or knee replacement, %									
Southeast MSA 1	19.1	13.4	−5.9 (−8.7 to −3.0)	9.3	−10.2 (−12.8 to −7.6)	6.9	−13.2 (−16.2 to −10.2)	3.9	−15.9 (−18.7 to −13.0)
Southeast MSA 2	48.4	21.1	−27.3 (−37.1 to −17.6)	13.2	−35.1 (−44.6 to −25.7)	10.1	−38.4 (−47.8 to −29.0)	4.6	−43.7 (−53.3 to −34.2)
South Central MSA	44.6	24.0	−20.2 (−28.5 to −12.0)	17.9	−26.5 (−34.7 to −18.3)	12.4	−31.5 (−39.7 to −23.3)	7.7	−36.5 (−44.6 to −28.4)
Midwest MSA	60.0	33.0	−27.4 (−33.6 to −21.1)	26.8	−32.9 (−39.1 to −26.7)	20.9	−38.9 (−45.1 to −32.7)	14.2	−45.5 (−51.8 to −39.3)
West MSA	64.8	42.1	−22.3 (−29.1 to −15.5)	30.4	−33.8 (−40.1 to −27.6)	23.8	−41.3 (−47.6 to −35.1)	16.6	−49.1 (−56.4 to −41.9)
Arthroscopy in patients with new hip or knee osteoarthritis, %									
Southeast MSA 1	3.1	7.4	4.4 (3.1 to 5.7)	13.0	10.3 (9.0 to 11.6)	20.3	17.4 (16.2 to 18.6)	30.7	27.9 (25.5 to 30.2)
Southeast MSA 2	2.1	6.2	4.2 (3.2 to 5.1)	10.4	8.6 (7.8 to 9.5)	16.7	14.7 (13.5 to 15.8)	27.3	25.0 (22.1 to 27.8)
South Central MSA	2.4	6.4	4.0 (3.3 to 4.7)	11.8	9.5 (8.9 to 10.2)	18.0	15.9 (15.0 to 16.8)	30.4	28.1 (26.3 to 29.9)
Midwest MSA	2.5	6.2	3.6 (3.1 to 4.2)	9.9	7.3 (6.7 to 7.9)	15.2	12.6 (11.9 to 13.2)	25.5	23 (21.7 to 24.3)
West MSA	3.4	7.8	4.5 (3.5 to 5.5)	12.0	8.8 (7.8 to 9.7)	17.2	13.9 (12.8 to 14.9)	26.7	22.9 (20.0 to 25.9)
**Orthopedic surgery and neurosurgery (spine)**									
Spinal fusion in patients with low back pain, %									
Southeast MSA 1	16.9	31.6	14.1 (8.5 to 19.8)	41.0	23.3 (18.0 to 28.6)	54.7	36.6 (30.9 to 42.3)	75.1	57.5 (49.3 to 65.7)
Southeast MSA 2	5.6	23.8	18.1 (14.5 to 21.7)	34.3	28.9 (25.3 to 32.6)	44.2	38.7 (34.4 to 43.0)	64.1	58.8 (50.1 to 67.5)
South Central MSA	22.5	34.3	11.6 (8.7 to 14.5)	44.3	19.8 (16.8 to 22.7)	56.6	32.7 (29.4 to 36.0)	79.2	55.4 (48.8 to 61.9)
Midwest MSA	11.3	25.0	13.9 (10.7 to 17.1)	35.0	23.4 (20.5 to 26.3)	42.2	30.8 (27.8 to 33.7)	57.3	45.0 (40.6 to 49.4)
West MSA	6.0	21.8	16.3 (12.7 to 20.0)	30.0	24.4 (21.5 to 27.3)	46.7	39.7 (35.4 to 44.0)	68.2	63.0 (58.2 to 67.8)
Any physical therapy in patients with new cervical spine pain, %									
Southeast MSA 1	53.3	30.4	−22.5 (−30.0 to −15.1)	20.9	−32.4 (−39.8 to −25.0)	13.0	−40.1 (−47.5 to −32.8)	5.7	−48.1 (−55.5 to −40.7)
Southeast MSA 2	51.4	37.4	−13.3 (−18.7 to −7.8)	30.1	−20.3 (−25.7 to −14.9)	20.2	−30.3 (−35.9 to −24.7)	10.4	−39.9 (−46.2 to −33.7)
South Central MSA	63.0	45.9	−17.3 (−21.5 to −13.2)	35.0	−27.9 (−31.9 to −23.9)	28.0	−34.5 (−38.6 to −30.4)	16.9	−46.1 (−50.5 to −41.7)
Midwest MSA	65.4	54.8	−10.6 (−13.1 to −8.1)	48.2	−17.1 (−19.5 to −14.7)	41.0	−24.2 (−26.7 to −21.8)	29.6	−36.0 (−39.1 to −32.9)
West MSA	62.0	51.9	−9.4 (−13.8 to −5.0)	44.5	−17.7 (−21.7 to −13.8)	37.7	−24.2 (−28.5 to −19.9)	23.3	−37.1 (−44.1 to −30.1)

Abbreviation: MSA, metropolitan statistical area.

^a^ Measure specifications and supporting clinical guidelines are provided in eMethods 1 in the Supplement. Means represent unadjusted averages of performance by quintile of physicians. Adjusted differences between quintile 1 [reference] and quintiles 2 through 5, respectively, are derived from the statistical model adjusted for patient risk and socioeconomic variables, with standard errors clustered by physician.

^b^ Appropriate prenatal screening is defined as a pregnancy having all of the following tests: Rh factor, oral glucose tolerance test, group B strep, and urinalysis. A low-risk pregnancy is defined as the absence of diagnoses that define a high-risk pregnancy: infections such as HIV, cardiovascular disease, diabetes, kidney disease, preeclampsia, HELLP (hemolysis, elevated liver enzymes and low platelets) syndrome, multiple gestation, alloimmunization, damage to fetus, poor fetal growth, oligohydramnios/polyhydramnios, placental abnormalities, uterine abnormalities, hemorrhage, complicated delivery, postterm pregnancy, and other related conditions.

^c^ Physical therapy prior refers to physical therapy 4 months prior to surgery. Elective is defined as an elective surgical admission in the absence of emergency care. New knee osteoarthritis is defined as the first year after the initial diagnosis of knee osteoarthritis in a patient whose first visit with an orthopedic surgeon was within 120 days of their first diagnosis of hip or knee pain.

### Sensitivity and Secondary Analyses

Adjusted differences between quintiles were similar in sensitivity analyses, which suggested that observable differences in patient age, sex, clinical diagnoses, and socioeconomic status characteristics across physician quintiles contributed minimal bias toward the differences in performance between the quintiles (eTables 1-7 in the Supplement). Across the 7 specialties, average spending on corresponding episodes of care also exhibited large variations for similar patients across physicians, which may reflect differences in prices or health care utilization (eTable 8 in the Supplement). However, the correlation between average unadjusted appropriateness or quality and episode-level spending was generally weak (eFigure 8 in the Supplement). In the South Central MSA, where most specialists in the measures were affiliated with a health system, physician-level variations within organizations were qualitatively larger than variations in average performance between organizations (eFigure 9 in the Supplement).

## Discussion

Within 5 US metropolitan areas, sizeable variations in practice patterns between physicians were evident across 14 clinical situations, where, on average, guideline-based appropriateness and quality of clinical decisions were observable in claims. These within-area variations in practice patterns, qualitatively similar across different specialties, were likely explained in large part by differences in clinical decision-making, to the extent that adjustment for patient- and area-level characteristics within these defined clinical scenarios further standardized the patients beyond the inclusion criteria. However, unobserved patient factors such as preferences, inaccurate coding of clinical diagnoses that would have altered the assessment of appropriateness, clinical considerations unobservable in claims (eg, allergies, availability of physical therapy), and other social determinants of health not captured in the present data may have influenced the results. Our efforts to carefully define the clinical scenarios and the patients in them, along with sensitivity analyses, suggest that these factors likely would not qualitatively change the results. However, for any given situation or patient, the potential for confounding could not be fully eliminated.

This evidence adds to the Institute of Medicine recommendation to focus on within-region variations in clinical decision-making as a target of policy and quality improvement.^3,4^ It builds on earlier efforts to measure physician-level differences in patient outcomes (though less often in specific guideline-based clinical decisions) such as surgical report cards, as well as the seminal Dartmouth Atlas of Health Care literature on between-region variations in quality.^30,31,32,33,34,35,36,37^ For consumers, employers, and insurers, within-region physician-level variations may be useful because moving or shifting enrollees across regions is less practical. We caution that heterogeneity in delivery systems, incentives, and other within-MSA differences still exist and could partially explain the variations.^7^ However, we found that between-physician variations within physician organizations were generally larger than between-organization variations in 1 MSA.

This study demonstrates that measuring variations in decision-making at the clinician level using carefully defined clinical situations is possible in large claims data. It provides basic examples of clinical scenarios where evidence-based guidelines could be expected to have reduced such variations. Owing to imperfect risk adjustment and potential confounders, we consider this work a proof of concept and believe that between-physician comparisons can be improved with further data (eg, clinical data). Nevertheless, these findings are illustrative in showing the extent of variation that remains among plausibly similar patients in similar clinical scenarios. Moreover, rigorous approaches to measure reliability can help improve on earlier efforts to study physician-level variations.^38^ If conveyed in a collaborative and nonpunitive way (eg, initially as an educational tool rather than a financial incentive), such evidence may lead clinicians and their organizations to explore practice patterns relative to peers and identify areas of improvement.^39^ Understanding why some guidelines are followed more uniformly than others may further inform clinical education and training.

Evidence of local practice pattern variations may also encourage employers, insurers, and clinicians to collaborate on quality improvement efforts. Such evidence may eventually help patients and purchasers choose among local physicians or design insurance plans in a higher-value way. Today, to the extent data are used at all by employers and insurers to nudge patients toward higher-value clinicians, only differences in prices or spending are typically used, partly owing to the challenges of measuring physician-level (or organizational-level) quality and appropriateness of care. While clinician resistance to such measurement might be expected, measures that adhere closely to accepted clinical guidelines, address clinical scenarios with large sample sizes, and include adequate risk adjustment might garner more physician support.

Even so, attaching direct financial incentives to such measures in a pay-for-performance context may produce unintended consequences. Various pay-for-performance programs in recent years that tied financial rewards to quality measures have not achieved meaningful or sustained quality improvement. On the contrary, unintended consequences have been common, such as gaming behavior (eg, selecting or avoiding certain patients),^40^ exacerbating inequalities (eg, better-resourced hospitals or organizations disproportionately rewarded),^41,42^ and contributing to physician administrative costs and burnout.^43^

### Limitations

We emphasize several limitations. First, the 14 measures captured limited dimensions of quality or appropriateness in these specialties. Second, the data lacked further observable patient characteristics (eg, family history), which limited our ability to adjust for additional confounders. Moreover, claims data may not fully capture the preceding symptom burden that may have triggered subsequent testing and therapies. To the extent that unobserved patient complexity may explain sorting of patients to physicians or the contributions of patient demand to clinical decision-making, the present results may overestimate differences attributed to physician decision-making. Evidence of substantial residual confounding after risk adjustment exists.^44^ Thus, we advocate caution in individual physician comparisons. Third, because care is frequently team based, measures may misattribute decision-making to the specialist. Notably, in measures concerning chronic disease management (coronary artery disease, diabetes, and chronic obstructive pulmonary disease), primary care physicians may influence performance, though it was difficult to discern whether decisions originated from specialists (such as through recommendations) or from primary care physicians. Similarly, we could not disentangle physician effects from hospital or organization-level effects. Although statistical models could accommodate additional random effects for the hospital or organization, physician-level effects are not easily distinguishable from them.^45^ Finally, commercially insured populations may not generalize to other populations, although prior studies of low-value care and regional variations have shown correlations between payer populations.^46^

## Conclusions

In this cross-sectional study of physicians in 5 US metropolitan areas, sizeable physician-level practice pattern variations were found across specialties and common clinical scenarios. In addition to payment reform, understanding physician-level variations in decision-making may provide a complementary direction for improving value that is more clinically nuanced.

## Notes

Supplement. **eMethods 1.** Clinical Guidelines and Evidence Behind Measure Definitions **eMethods 2.** Measure Development and Statistical Analysis **eMethods 3.** Measures by Specialty and Number of Patients per Physician by Measure **eFigure 1.** Physician-Level Variation in Cardiologist Coronary Artery Disease Care **eFigure 2.** Physician-Level Variation in Endocrinologist Diabetes Care **eFigure 3.** Physician-Level Variation in Gastroenterologist Gastrointestinal Tract Care **eFigure 4.** Physician-Level Variation in Pulmonologist COPD Care **eFigure 5.** Physician-Level Variation in Obstetrician Prenatal and Delivery Care **eFigure 6.** Physician-Level Variation in Orthopedist Joint Care **eFigure 7.** Physician-Level Variation in Orthopedist/Neurosurgeon Spine Care **eFigure 8.** Relationship Between Quality and Cost at the Physician Level **eFigure 9.** Physician-Level Variations in Practice Patterns within Organizations **eTable 1.** Physician-Level Variation in Cardiologist Coronary Artery Disease Care **eTable 2.** Physician-Level Variation in Endocrinologist Diabetes Care **eTable 3.** Physician-Level Variation in Gastroenterologist Gastrointestinal Tract Care **eTable 4.** Physician-Level Variation in Pulmonologist COPD Care **eTable 5.** Physician-Level Variation in Obstetrician Prenatal and Delivery Care **eTable 6.** Physician-Level Variation in Orthopedist Joint Care **eTable 7.** Physician-Level Variation in Orthopedist/Neurosurgeon Spine Care **eTable 8.** Physician-Level Variations in Health Care Spending within Domains of Care
