# Supplementary material for: Physician Practice Pattern Variations in Common Clinical Scenarios Within 5 US Metropolitan Areas
Source: JAMA Health Forum. 2022 Jan 28;3(1):e214698. doi: 10.1001/jamahealthforum.2021.4698 (PMC8903123; doi:10.1001/jamahealthforum.2021.4698)
Supplement: Supplement. — eMethods 1. Clinical Guidelines and Evidence Behind Measure Definitions eMethods 2. Measure Development and Statistical Analysis eMethods 3. Measures by Specialty and Number of Patients per Physician by Measure eFigure 1. Physician-Level Variation in Cardiologist Coronary Artery Disease Care eFigure 2. Physician-Level Variation in Endocrinologist Diabetes Care eFigure 3. Physician-Level Variation in Gastroenterologist Gastrointestinal Tract Care eFigure 4. Physician-Level Variation in Pulmonologist COPD Care eFigure 5. Physician-Level Variation in Obstetrician Prenatal and Delivery Care eFigure 6. Physician-Level Variation in Orthopedist Joint Care eFigure 7. Physician-Level Variation in Orthopedist/Neurosurgeon Spine Care eFigure 8. Relationship Between Quality and Cost at the Physician Level eFigure 9. Physician-Level Variations in Practice Patterns within Organizations eTable 1. Physician-Level Variation in Cardiologist Coronary Artery Disease Care eTable 2. Physician-Level Variation in Endocrinologist Diabetes Care eTable 3. Physician-Level Variation in Gastroenterologist Gastrointestinal Tract Care eTable 4. Physician-Level Variation in Pulmonologist COPD Care eTable 5. Physician-Level Variation in Obstetrician Prenatal and Delivery Care eTable 6. Physician-Level Variation in Orthopedist Joint Care eTable 7. Physician-Level Variation in Orthopedist/Neurosurgeon Spine Care eTable 8. Physician-Level Variations in Health Care Spending within Domains of Care [file jamahealthforum-e214698-s001.pdf]

Supplemental Online Content

Song Z, Kannan S, Gambrel RJ, et al. Physician practice pattern variations in common clinical scenarios within 5 US metropolitan areas. *JAMA Health Forum*. 2022;3(1):e214698. doi:10.1001/jamahealthforum.2021.4698

eMethods 1. Clinical Guidelines and Evidence Behind Measure Definitions
eMethods 2. Measure Development and Statistical Analysis
eMethods 3. Measures by Specialty and Number of Patients per Physician by Measure
eFigure 1. Physician-Level Variation in Cardiologist Coronary Artery Disease Care
eFigure 2. Physician-Level Variation in Endocrinologist Diabetes Care
eFigure 3. Physician-Level Variation in Gastroenterologist Gastrointestinal Tract Care
eFigure 4. Physician-Level Variation in Pulmonologist COPD Care
eFigure 5. Physician-Level Variation in Obstetrician Prenatal and Delivery Care
eFigure 6. Physician-Level Variation in Orthopedist Joint Care
eFigure 7. Physician-Level Variation in Orthopedist/Neurosurgeon Spine Care
eFigure 8. Relationship Between Quality and Cost at the Physician Level
eFigure 9. Physician-Level Variations in Practice Patterns within Organizations
eTable 1. Physician-Level Variation in Cardiologist Coronary Artery Disease Care
eTable 2. Physician-Level Variation in Endocrinologist Diabetes Care
eTable 3. Physician-Level Variation in Gastroenterologist Gastrointestinal Tract Care
eTable 4. Physician-Level Variation in Pulmonologist COPD Care
eTable 5. Physician-Level Variation in Obstetrician Prenatal and Delivery Care
eTable 6. Physician-Level Variation in Orthopedist Joint Care
eTable 7. Physician-Level Variation in Orthopedist/Neurosurgeon Spine Care
eTable 8. Physician-Level Variations in Health Care Spending within Domains of Care

This supplemental material has been provided by the authors to give readers additional information about their work.

© 2022 Song Z et al. *JAMA Health Forum.*

eMethods 1. Clinical Guidelines and Evidence Behind Measure Definitions

The measures assessed in this study were informed by professional guidelines and the clinical literature. Guidelines were generally developed by specialty societies whose expertise pertain to the domain of clinical care that the measure addresses. The peer-reviewed clinical literature in most instances uses similar measures as endpoints in studies of analogous patient populations. The following offers a review of the key professional society guidelines and clinical literature that informed the development of measure definitions used in this study.

A. Cardiologist Coronary Artery Disease Care

Stress tests in patients with stable chronic coronary artery disease (CAD)

Guidance: In patients who have no new or worsening symptoms or no prior evidence of silent ischemia and are not at high risk for a recurrent cardiac event, the usefulness of annual surveillance exercise ECG testing is not well established. Nuclear MPI, echocardiography, or CMR, with either exercise or pharmacological stress or CCTA, is not recommended for follow-up assessment in patients with SIHD, if performed more frequently than at a) 5-year intervals after CABG or b) 2-year intervals after PCI.

Reference:

- Finh SD, Gardin JM, Abrams J, Berra K, Blankenship JC, Dallas AP, et al. 2012 ACCF/AHA/ACP/AATS/PCNA/SCAI/STS guideline for the diagnosis and management of patients with stable ischemic heart disease: a report of the American College of Cardiology Foundation/American Heart Association task force on practice guidelines, and the American College of Physicians, American Association for Thoracic Surgery, Preventive Cardiovascular Nurses Association, Society for Cardiovascular Angiography and Interventions, and Society of Thoracic Surgeons. *Circulation*. 2012 Dec 18;126(25):e354-471.
- Garner KK, Pomeroy W, Arnold JJ. Exercise Stress Testing: Indications and Common Questions. *Am Fam Physician*. 2017 Sep 1;96(5):293-299.
- Wolk MJ, Bailey SR, Doherty JU, Douglas PS, Hendel RC, Kramer CM, Min JK, Patel MR, Rosenbaum L, Shaw LJ, Stainback RF, Allen JM; American College of Cardiology Foundation Appropriate Use Criteria Task Force. ACCF/AHA/ASE/ASNC/HFSA/HRS/SCAI/SCCT/SCMR/STS 2013 multimodality appropriate use criteria for the detection and risk assessment of stable ischemic heart disease: a report of the American College of Cardiology Foundation Appropriate Use Criteria Task Force, American Heart Association, American Society of Echocardiography, American Society of Nuclear Cardiology, Heart Failure Society of America, Heart Rhythm Society, Society for Cardiovascular Angiography and Interventions, Society of Cardiovascular Computed Tomography, Society for Cardiovascular Magnetic Resonance, and Society of Thoracic Surgeons. *J Am Coll Cardiol*. 2014 Feb 4;63(4):380-406.

© 2022 Song Z et al. *JAMA Health Forum.*

Statin therapy in patients with chronic coronary artery disease (CAD)

Guidance: In patients who are 75 years of age or younger with clinical atherosclerotic cardiovascular disease (ASCVD), high-intensity statin therapy should be initiated or continued with the aim of achieving a 50% or greater reduction in LDL-C levels.

Reference:

- Grundy SM, Stone NJ, Bailey AL, Beam C, Birtcher KK, Blumenthal RS. 2018 AHA/ACC/AACVPR/AAPA/ABC/ACPM/ADA/AGS/APhA/ASPC/NLA/PCNA Guideline on the Management of Blood Cholesterol: A Report of the American College of Cardiology/American Heart Association Task Force on Clinical Practice Guidelines. *J Am Coll Cardiol*. 2019 Jun 25;73(24):e285-e350.
- Arnett DK, Blumenthal RS, Albert MA, Buroker AB, Goldberger ZD, Hahn EJ, Himmelfarb CD, Khera A, Lloyd-Jones D, McEvoy JW, Michos ED, Miedema MD, Muñoz D, Smith SC Jr, Virani SS, Williams KA Sr, Yeboah J, Ziaeian B. 2019 ACC/AHA Guideline on the Primary Prevention of Cardiovascular Disease: A Report of the American College of Cardiology/American Heart Association Task Force on Clinical Practice Guidelines. *J Am Coll Cardiol*. 2019 Sep 10;74(10):e177-e232.

B. Endocrinologist Diabetes Care

Kidney function test in patients with diabetes

Guidance: In patients with type 2 diabetes, an annual screening test of kidney function is indicated according to practice guidelines.

References:

- American Diabetes Association. 11. Microvascular complications and footcare: Standards of Medical Care in Diabetes—2019. *Diabetes Care*. 2019; 42: S124-S138.
- Kramer H, Molitch ME. Screening for kidney disease in adults with diabetes. *Diabetes Care*. 2005 Jul;28(7):1813-6.

Oral glucose-lowering medication in patients with diabetes

Guidance: In patients with type 2 diabetes, practice guidelines from the American Diabetes Association and the American College of Physicians recommend that clinicians use glucose-lowering agents to improve glycemic control. Metformin is the preferred initial pharmacologic agent for the treatment of type 2 diabetes. A patient-centered approach that includes additional pharmacotherapy, including sodium-glucose contransporter-2 (SGLT-2) inhibitors and glucagon-like peptide (GLP-1) agonists, is strongly recommended for patients with type 2 diabetes who have cardiovascular and renal comorbidities. In this study, we focused on oral glucose-lowering medications, which reflects slightly older guidance prior to the widespread adoption of newer medications, given that our data were from 2016-2019.

© 2022 Song Z et al. *JAMA Health Forum.*

References:

- Qaseem A, Barry MJ, Humphrey LL, Forciea MA; Clinical Guidelines Committee of the American College of Physicians. Oral Pharmacologic Treatment of Type 2 Diabetes Mellitus: A Clinical Practice Guideline Update From the American College of Physicians. *Ann Intern Med*. 2017 Feb 21;166(4):279-290.
- Qaseem A, Humphrey LL, Sweet DE, Starkey M, Shekelle P; Clinical Guidelines Committee of the American College of Physicians. Oral pharmacologic treatment of type 2 diabetes mellitus: a clinical practice guideline from the American College of Physicians. *Ann Intern Med*. 2012 Feb 7;156(3):218-31.
- American Diabetes Association. 9. Pharmacologic Approaches to Glycemic Treatment: Standards of Medical Care in Diabetes-2020. *Diabetes Care*. 2020 Jan;43(Suppl 1):S98-S110.
- Grundy SM, Stone NJ, Bailey AL, Beam C, Birtcher KK, Blumenthal RS, Braun LT, de Ferranti S, Faiella-Tommasino J, Forman DE, Goldberg R, Heidenreich PA, Hlatky MA, Jones DW, Lloyd-Jones D, Lopez-Pajares N, Ndumele CE, Orringer CE, Peralta CA, Saseen JJ, Smith SC Jr, Sperling L, Virani SS, Yeboah J. 2018 AHA/ACC/AACVPR/AAPA/ABC/ACPM/ADA/AGS/APhA/ASPC/NLA/PCNA Guideline on the Management of Blood Cholesterol: A Report of the American College of Cardiology/American Heart Association Task Force on Clinical Practice Guidelines. *Circulation*. 2019 Jun 18;139(25):e1082-e1143.

C. Gastroenterologist Gastrointestinal Tract Care

Polyp detection on screening colonoscopy

Guidance: An adenoma detection rate is defined as the rate by which a physician finds one or more precancerous polyps during a normal screening colonoscopy. Professional societies, including the American College of Gastroenterology, have determined the benchmark rate should be at least 25% in men and 15% in women. Evidence shows that a 1% increase in the adenoma detection rate was associated with a 3% decrease in the risk of colorectal cancer and reduced cancer mortality. This general inverse relationship has been found in multiple studies. Because the composition of a polyp is unknown to the gastroenterologist at the time of the colonoscopy, a gastroenterologist who finds disproportionately more non-adenomatous polyps may be penalized by the narrow definition centered on adenomatous polyps. Thus, we expanded the measure definition to include all polyps detected during the colonoscopy.

References:

- Corley DA, Jensen CD, Marks AR, Zhao WK, Lee JK, Doubeni CA, Zauber AG, de Boer J, Fireman BH, Schottinger JE, Quinn VP, Ghai NR, Levin TR, Quesenberry CP. Adenoma detection rate and risk of colorectal cancer and death. *N Engl J Med*. 2014 Apr 3;370(14):1298-306.
- Kaminski MF, Wieszczy P, Rupinski M, Wojciechowska U, Didkowska J, Kraszewska E, Kobiela J, Franczyk R, Rupinska M, Kocot B, Chaber-Ciopinska A, Pachlewski J,

© 2022 Song Z et al. *JAMA Health Forum.*

Polkowski M, Regula J. Increased Rate of Adenoma Detection Associates With Reduced Risk of Colorectal Cancer and Death. *Gastroenterology*. 2017 Jul;153(1):98-105.
- Rex DK, Schoenfeld PS, Cohen J, Pike IM, Adler DG, Fennerty MB, Lieb JG 2nd, Park WG, Rizk MK, Sawhney MS, Shaheen NJ, Wani S, Weinberg DS. Quality indicators for colonoscopy. *Am J Gastroenterol*. 2015 Jan;110(1):72-90.
- Lieberman DA, Rex DK, Winawer SJ, Giardiello FM, Johnson DA, Levin TR. Guidelines for colonoscopy surveillance after screening and polypectomy: a consensus update by the US Multi-Society Task Force on Colorectal Cancer. *Gastroenterology*. 2012 Sep;143(3):844-857.

Endoscopy in patients with GERD and no alarm symptoms

Guidance: Endoscopy is generally not recommended in patients with gastroesophageal reflux disease in the absence of alarm symptoms, such as weight loss, anemia, bleeding, dysphagia, and odynophagia. These alarm symptoms are associated with esophagitis and peptic strictures, and the presence of alarm symptoms render endoscopy more clinically appropriate.

References:

- DeVault KR, Castell DO; American College of Gastroenterology. Updated guidelines for the diagnosis and treatment of gastroesophageal reflux disease. *Am J Gastroenterol*. 2005 Jan;100(1):190-200.
- Katz PO, Gerson LB, Vela MF. Guidelines for the diagnosis and management of gastroesophageal reflux disease. *Am J Gastroenterol*. 2013 Mar;108(3):308-28.
- Shaheen NJ, Weinberg DS, Denberg TD, Chou R, Qaseem A, Shekelle P; Clinical Guidelines Committee of the American College of Physicians. Upper endoscopy for gastroesophageal reflux disease: best practice advice from the clinical guidelines committee of the American College of Physicians. *Ann Intern Med*. 2012 Dec 4;157(11):808-16.
- ASGE Standards of Practice Committee. The role of endoscopy in the management of GERD. *Gastrointest Endosc*. 2015;81(6):1305-10.

D. Pulmonologist Chronic Obstructive Pulmonary Disease (COPD) Care

Spirometry in patients with chronic obstructive pulmonary disease (COPD)

Guidance: The GOLD international COPD guidelines and other national guidelines recommend spirometry as the gold standard for accurate and repeatable measurement of lung function in patients with COPD. Evidence suggests that when spirometry confirms the presence of COPD, physicians initiate more appropriate treatment.

References:

- Global Initiative for Chronic Obstructive Lung Disease. Global strategy for the diagnosis, management, and prevention of chronic obstructive pulmonary disease. (Updated 2007). (http://www.goldcopd.org.)

© 2022 Song Z et al. *JAMA Health Forum.*

- Pauwels RA, Buist AS, Calverley PM, Jenkins CR, Hurd SS; GOLD Scientific Committee. Global strategy for the diagnosis, management, and prevention of chronic obstructive pulmonary disease. NHLBI/WHO Global Initiative for Chronic Obstructive Lung Disease (GOLD) Workshop summary. *Am J Respir Crit Care Med*. 2001 Apr;163(5):1256-76.
- National Collaborating Centre for Chronic Conditions. Chronic obstructive pulmonary disease: national clinical guideline on management of chronic obstructive pulmonary disease in adults in primary and secondary care. *Thorax* 2003, 59 (Suppl 1); 1-232.

Bronchodilator in patients with chronic obstructive pulmonary disease (COPD)

Guidance: Inhaled bronchodilators in chronic obstructive pulmonary disease (COPD) are central to preventing and reducing symptoms, as well as improving lung function. The NHLBI/WHO Global Initiative for Chronic Obstructive Lung Disease (GOLD) guidelines state that bronchodilator medications are central to the management of COPD.

References:

- Singh D, Agusti A, Anzueto A, Barnes PJ, Bourbeau J, Celli BR, et al. Global Strategy for the Diagnosis, Management, and Prevention of Chronic Obstructive Lung Disease: the GOLD science committee report 2019. *Eur Respir J*. 2019 May 18;53(5):1900164.
- Gentry S, Gentry B. Chronic Obstructive Pulmonary Disease: Diagnosis and Management. *Am Fam Physician*. 2017 Apr 1;95(7):433-441.
- Pauwels RA, Buist AS, Calverley PM, Jenkins CR, Hurd SS; GOLD Scientific Committee. Global strategy for the diagnosis, management, and prevention of chronic obstructive pulmonary disease. NHLBI/WHO Global Initiative for Chronic Obstructive Lung Disease (GOLD) Workshop summary. *Am J Respir Crit Care Med*. 2001 Apr;163(5):1256-76.
- National Heart, Lung, and Blood Institute. *Guidelines for the Diagnosis and Management of Asthma (EPR-3)*. 2012 Sep.

E. Obstetrician Prenatal and Delivery Care

Appropriate prenatal screening in pregnant patients

Guidance: Appropriate prenatal screening tests are recommended. Routine tests include the Rh factor, oral glucose tolerance test, group B strep, and a urinalysis.

References:

- American Congress of Obstetricians and Gynecologists/American Academy of Pediatrics. *2012 Guidelines for Perinatal Care (7th Edition)*. Zolotor AJ, Carlough MC. Update on prenatal care. *Am Fam Physician*. 2014 Feb 1;89(3):199-208.
- American College of Obstetricians and Gynecologists' Committee on Practice Bulletins—Obstetrics. Antepartum Fetal Surveillance: ACOG Practice Bulletin Summary, Number 229. *Obstet Gynecol*. 2021 Jun 1;137(6):1134-1136.

© 2022 Song Z et al. *JAMA Health Forum.*

Caesarean delivery in patients with low-risk pregnancies

Guidance: Cesarean delivery is the most common major surgery in the U.S., used in over a quarter of low-risk births and about one in three births overall. In the absence of maternal or fetal indications for cesarean delivery, the American College of Obstetricians and Gynecologists and multiple public health and professional societies have advised that a plan for vaginal delivery is safe and appropriate and should be recommended. Reducing the use of cesarean procedures in first-time deliveries is also a goal of Health People 2030.

References:

- American College of Obstetricians and Gynecologists (College); Society for Maternal-Fetal Medicine, Caughey AB, Cahill AG, Guise JM, Rouse DJ. Safe prevention of the primary cesarean delivery. *Am J Obstet Gynecol*. 2014 Mar;210(3):179-93.
- Spong CY, Berghella V, Wenstrom KD, Mercer BM, Saade GR. Preventing the first cesarean delivery: summary of a joint Eunice Kennedy Shriver National Institute of Child Health and Human Development, Society for Maternal-Fetal Medicine, and American College of Obstetricians and Gynecologists Workshop. *Obstet Gynecol*. 2012 Nov;120(5):1181-93.
- Healthy People 2030. Reduce cesarean births among low-risk women with no prior births—MICH-06. Washington (DC): Department of Health and Human Services, Office of Disease Prevention and Health Promotion; 2020. (https://health.gov/healthypeople/objectives-and-data/browse-objectives/pregnancy-and-childbirth/reduce-cesarean-births-among-low-risk-women-no-prior-births-mich-06)

F. Orthopedist Joint Care

Any physical therapy (PT) prior to elective hip or knee replacement

Guidance: The clinical practice guideline on the Management of Osteoarthritis of the Hip: Evidence-Based Clinical Practice Guideline from the American Academy of Orthopaedic Surgeons (AAOS) indicates strong evidence in support of physical therapy as a treatment to improve function and reduce pain for patients with osteoarthritis of the hip and mild to moderate symptoms, and limited evidence which supports that supervised exercise before total knee arthroplasty might improve pain and physical function after surgery. The American Physical Therapy Association endorses preoperative physical therapy, based evidence of improved outcomes after surgery. In other clinical literature as discussed by the Agency for Healthcare Research and Quality, physical therapy prior to total joint replacement has demonstrated either favorable or non-inferior effects on patient outcomes, ranging from evidence of decreased use of post-acute services, to improved post-operative function, to no additional benefits.

References:

- Rees HW. Management of Osteoarthritis of the Hip. *J Am Acad Orthop Surg*. 2020 Apr 1;28(7):e288-e291.

- Snow R, Granata J, Ruhil AV, et al. Associations between preoperative physical therapy and post-acute care utilization patterns and cost in total joint replacement. *J Bone Joint Surg Am*. 2014 Oct 1;96(19):e165.
- Moyer R, Ikert K, Long K, et al. The Value of Preoperative Exercise and Education for Patients Undergoing Total Hip and Knee Arthroplasty: A Systematic Review and Meta-Analysis. *JBJS Rev*. 2017 Dec;5(12):e2.
- Calatayud J, Casana J, Ezzatvar Y, Jakobsen MD, Sundstrup E, Andersen LL. High-intensity preoperative training improves physical and functional recovery in the early post-operative periods after total knee arthroplasty: a randomized controlled trial. *Knee Surg Sports Traumatol Arthrosc*. 2017;25:2864–2872.
- Evgeniadis G, Beneka A, Malliou P, Mavromoustakos S, Godolias G. Effects of pre- or postoperative therapeutic exercise on the quality of life, before and after total knee arthroplasty for osteoarthritis. *J Back Musculoskel Rehabil*. 2008;21:161–169.
- Matassi F, Duerinckx J, Vandenneucker H, Bellemans J. Range of motion after total knee arthroplasty: the effect of a preoperative home exercise program. *Knee Surg Sports Traumatol Arthrosc*. 2014;22:703–709.
- McKay C, Prapavessis H, Doherty T. The effect of a prehabilitation exercise program on quadriceps strength for patients undergoing total knee arthroplasty: a randomized controlled pilot study. *PM&R*. 2012;4:647–656.
- Jette DU, Hunter SJ, Burkett L, Langham B, Logerstedt DS, Piuzzi NS, Poirier NM, Radach LJL, Ritter JE, Scalzitti DA, Stevens-Lapsley JE, Tompkins J, Zeni J Jr; American Physical Therapy Association. Physical Therapist Management of Total Knee Arthroplasty. *Phys Ther*. 2020 Aug 31;100(9):1603-1631.

Arthroscopy in patients with new hip or knee osteoarthritis

<u>Guidance</u>: Multiple randomized controlled trials have shown that in patients with knee osteoarthritis, outcomes after arthroscopic lavage or debridement were no better than those after placebo, and that arthroscopy provided no additional benefit relative to optimized physical and medical therapy. This is especially true in isolated knee osteoarthritis, in which guidelines advise no role for arthroscopy. In cases of knee osteoarthritis with meniscal tear, randomized controlled trials and meta-analyses have also shown similar outcomes between arthroscopy and physical therapy. Moreover, arthroscopic abrasion arthroplasty has had mixed results in osteoarthritis, including worsened symptoms and function and roughly half of patients undergoing total knee replacement within 3 years. In hip osteoarthritis, there is also no established role for arthroscopy, although no known randomized controlled trials have been published. There is some evidence that arthroscopic debridement of the labrum has minimal benefit in patients older than 45 years. Rigorous data among younger populations are not yet broadly available.

<u>References</u>:

- Moseley JB, O'Malley K, Petersen NJ, Menke TJ, Brody BA, Kuykendall DH, et al. A controlled trial of arthroscopic surgery for osteoarthritis of the knee. *N Engl J Med*. 2002 Jul 11;347(2):81-8.

© 2022 Song Z et al. *JAMA Health Forum.*

- Kirkley A, Birmingham TB, Litchfield RB, Giffin JR, Willits KR, Wong CJ, et al. A randomized trial of arthroscopic surgery for osteoarthritis of the knee. *N Engl J Med*. 2008 Sep 11;359(11):1097-107.
- Siemieniuk RAC, Harris IA, Agoritsas T, Poolman RW, Brignardello-Petersen R, Van de Velde S, et al. Arthroscopic surgery for degenerative knee arthritis and meniscal tears: a clinical practice guideline. *BMJ*. 2017 May 10;357:j1982.
- Katz JN, Brophy RH, Chaisson CE, de Chaves L, Cole BJ, Dahm DL, Donnell-Fink LA, Guermazi A, Haas AK, Jones MH, Levy BA, Mandl LA, Martin SD, Marx RG, Miniaci A, Matava MJ, Palmisano J, Reinke EK, Richardson BE, Rome BN, Safran-Norton CE, Skoniecki DJ, Solomon DH, Smith MV, Spindler KP, Stuart MJ, Wright J, Wright RW, Losina E. Surgery versus physical therapy for a meniscal tear and osteoarthritis. *N Engl J Med*. 2013;368(18):1675.
- Thorlund JB, Juhl CB, Roos EM, Lohmander LS. Arthroscopic surgery for degenerative knee: Systematic review and meta-analysis of benefits and harms. *BMJ*. 2015;350:h2747.
- Herrlin S, Hållander M, Wange P, Weidenhielm L, Werner S. Arthroscopic or conservative treatment of degenerative medial meniscal tears: A prospective randomised trial. *Knee Surg Sports Traumatol Arthrosc*. 2007;15(4):393.
- Herrlin SV, Wange PO, Lapidus G, Hållander M, Werner S, Weidenhielm L. Is arthroscopic surgery beneficial in treating non-traumatic, degenerative medial meniscal tears? A five year follow-up. *Knee Surg Sports Traumatol Arthrosc*. 2013 Feb;21(2):358-64.
- Khan M, Evaniew N, Bedi A, Ayeni OR, Bhandari M. Arthroscopic surgery for degenerative tears of the meniscus: A systematic review and meta-analysis. *CMAJ*. 2014;186(14):1057.
- Wilkin G, March G, Beaulé PE. Arthroscopic acetabular labral debridement in patients forty-five years of age or older has minimal benefit for pain and function. *J Bone Joint Surg Am*. 2014 Jan;96(2):113-8.
- Rand JA. Role of arthroscopy in osteoarthritis of the knee. *Arthroscopy*. 1991;7(4):358.
- Mounsey A, Ewigman B. Arthroscopic surgery for knee osteoarthritis? Just say no. *J Fam Pract*. 2009 Mar;58(3):143-5.

G. Orthopedist/Neurosurgeon Low Back Care

Spinal fusion in patients with low back pain

Guidance: Guidelines indicates that only a small minority of patients with low back pain ever require surgery, and this small proportion of patients have a clear clinical indication including severe or progressive motor weakness and signs and symptoms of cauda equina syndrome. There is no evidence that early referral for surgery in the absence of severe or progressive neurologic deficits improves outcomes for main types of low back pain including lumbar disc prolapse with radiculopathy and spinal stenosis (UptoDate®). A four-year follow-up of two randomized controlled trials found no benefit of spinal fusion surgery compared to nonsurgical treatment. Meta-analyses of the clinical literature including randomized controlled trials found no clear benefit of lumbar vertebral fusion compared to nonsurgical treatment. One key nuance is that surgery seemed to be more effective than

© 2022 Song Z et al. *JAMA Health Forum.*

unstructured non-surgical management, but was not more effective than structured or intensive physical therapy.

References:

- Treatment of degenerative lumbar spinal stenosis: summary. Evidence report/technology assessment number 32. Rockville, MD: Agency for Healthcare Research and Quality, June 2001. (AHRQ publication no. 01-E047.)
- Chou R, Baisden J, Carragee EJ, Resnick DK, Shaffer WO, Loeser JD. Surgery for low back pain: a review of the evidence for an American Pain Society Clinical Practice Guideline. *Spine* (Phila Pa 1976). 2009;34(10):1094.
- Mirza SK, Deyo RA. Systematic review of randomized trials comparing lumbar fusion surgery to nonoperative care for treatment of chronic back pain. *Spine* (Phila Pa 1976). 2007;32(7):816.
- Brox JI, Nygaard ØP, Holm I, Keller A, Ingebrigtsen T, Reikerås O. Four-year follow-up of surgical versus non-surgical therapy for chronic low back pain. *Ann Rheum Dis*. 2010;69(9):1643.
- Weinstein JN, Lurie JD, Olson PR, et al. United States' trends and regional variations in lumbar spine surgery: 1992-2003. *Spine* (Phila Pa 1976) 2006; 31:2707.
- Weber H. Lumbar disc herniation. A controlled, prospective study with ten years of observation. *Spine* (Phila Pa 1976) 1983; 8:131.
- Vroomen PC, de Krom MC, Knottnerus JA. Predicting the outcome of sciatica at short-term follow-up. *Br J Gen Pract.* 2002; 52:119.

Any physical therapy (PT) in patients with new cervical spine pain (%)

Guidance: For patients with newly diagnosed cervical spine pain, multiple guidelines recommend physical therapy prior to surgery. Failure to respond to physical therapy, along with failure of other non-invasive or conservative management, is part of the indication for surgery. Therefore, in this measure comprising patients 35 years or older with new cervical spine pain who saw a spine surgeon within 120 days of the diagnosis, we measure the share of patients who received physical therapy within 120 days of the first visit. To improve measure validity, patients who were seen by the spine surgeon more than 120 days after the initial diagnosis of cervical spine pain were excluded.

References:

- Hsu JR, Mir H, Wally MK, Seymour RB; Orthopaedic Trauma Association Musculoskeletal Pain Task Force. Clinical Practice Guidelines for Pain Management in Acute Musculoskeletal Injury. *J Orthop Trauma*. 2019 May;33(5):e158-e182.
- Childress MA, Becker BA. Nonoperative Management of Cervical Radiculopathy. *Am Fam Physician*. 2016 May 1;93(9):746-54.
- Blanpied PR, Gross AR, Elliott JM, Devaney LL, Clewley D, Walton DM, Sparks C, Robertson EK. Neck Pain: Revision 2017: Clinical Practice Guidelines Linked to the International Classification of Functioning, Disability and Health From the Orthopaedic Section of the American Physical Therapy Association. *J Orthop Sports Phys Ther*. 2017 Jul;47(7):A1-A83.

© 2022 Song Z et al. *JAMA Health Forum.*

eMethods 2: Measure Development and Statistical Analysis

This section of the supplementary appendix provides additional information on measure development and statistical analysis.

A. Clinical Scenarios

Through an extensive process of literature review, assessment of current measures in the public domain (e.g. National Quality Forum, Centers for Medicare and Medicaid Services, and Agency for Healthcare Research and Quality), and through consultations with clinical subject matter experts, we defined measures of appropriate care in a set of 14 clinical scenarios spanning 7 specialties: coronary artery disease care (stress tests in patients with stable chronic coronary artery disease and statin therapy in patients with chronic coronary artery disease), endocrinologist diabetes care (kidney function testing and oral glucose-lowering medication therapy in patients with diabetes), gastroenterologist gastrointestinal care (polyp detection rates on screening colonoscopy and endoscopy in patients with gastroesophageal reflux disease and no alarm symptoms), pulmonologist COPD and asthma care (bronchodilator and spirometry in patients with COPD), obstetrician prenatal and delivery care (appropriate prenatal screening in pregnant patients and Caesarean delivery in patients with low-risk pregnancy), orthopedist joint care (any physical therapy prior to elective hip or knee replacement and arthroscopy in patients with new hip or knee osteoarthritis), and orthopedist or neurosurgeon spine care (spinal fusion in patients with low back pain and physical therapy in patients with cervical spine pain).

In defining these measures, we selected clinical scenarios that are common. For example, back pain is one of the most common chief complaints presented to physicians in the outpatient setting in the U.S. Similarly, childbirth is a common event in the general population. Second, we focused on clinical scenarios in which specific observable events reflecting appropriateness of care would be plausibly captured in the administrative claims data. Observable events included the presence of a clinical service, test, or prescription drug. We assessed such events in a defined population of patients who were in each clinical scenario (e.g. physical therapy prior to elective hip or knee replacement and arthroscopy in patients with new hip or knee osteoarthritis).

With attention toward measure validity, we defined the measures to be appropriate for specific patient populations observable in the claims data as recommended in practice guidelines, the literature, or established quality measurement definitions. For example, A low-risk pregnancy was defined as the absence of multiple gestation; maternal endocrine, gastrointestinal, or cardiovascular conditions (either pre-existing or gestational); conditions of the placenta, amniotic fluid, and uterus; and fetal conditions including fetal abnormalities, fetal demise, and reduced fetal movement or growth (more details can be found in eFigures 1-7).

To improve measure validity, 9 of the 14 measures largely adhered to specifications from the National Quality Forum, NCQA Healthcare Effectiveness Data and Information Set, Agency for Healthcare Research and Quality, and Centers for Medicare and Medicaid Services. These specifications have gone through rigorous peer review and are commonly used by Medicare and commercial insurers. For measures focused on clinically indicated medications, such as the share of patients with coronary artery disease on a statin or share of patients with diabetes mellitus on an oral glucose-lowering agent, patient-level adherence was measured each month. Aggregated to the physician-level, such a measure reflects the proportion of months a physician’s attributed patients were on the indicated medication.

Given that we estimated physician-level variations in measure performance, we linked a given measure to the specialty most likely responsible for performance on the measure (e.g. for measures on child birth, we linked child births to the obstetrician on the delivery claim). Some measures, however, such as patients with coronary artery disease on a statin (which we defined as a cardiologist measure), could be influenced by other specialties such as primary care. In the data, we were not able to disentangle how much of practice patterns was driven by primary care, recommended by specialists and implemented by other clinicians, or driven by specialists. Thus, to the extent that performance was driven by primary care or other clinicians, this study attributes that to the specialist, who would plausibly review those clinical decisions by colleagues and modify them as necessary upon seeing the patient.

We calculated the reliability of each measure for each physician using the signal to noise approach, which examines the ratio of the variation between physicians and total variation within a measure, the latter comprising between-provider plus within-provider variation. A linear random effects model was fit to estimate the clinical event of interest (e.g., the numerator), with the provider as the independent variable, and a random intercept. The covariance parameter estimate serves as the between-provider variation; the within-provider variation was calculated by the sum of the squared residuals from the model, divided by N(N-1). Consistent with prior work, a reliability greater than 0.7 was considered high and a reliability between 0.4 and 0.7 was considered acceptable. To improve the reliability of measures and exclude physicians with low case volumes, we defined a minimum threshold of 10 patient cases conforming to a measure's clinical scenario that a physician must have to be included in the measure. For example, for measures concerning low-risk pregnancy, an obstetrician must have performed at least 10 low-risk deliveries to be eligible. The average and interquartile range of number of patients per physician for each measure is shown in eMethods 3.

We assigned specialties using the principal specialty code in the claims. Physician specialties were defined systematically using the National Uniform Claim Committee (NUCC) Provider Taxonomy as reported in the Center for Medicare and Medicaid Services (CMS) National Plan and Provider Enumeration System (NPPES).

B. Statistical Analysis

Within each measure, adjusted differences of physician performance were estimated using data at the patient level; in other words, the patient was the unit of analysis. We used a standard model that compared patients attributed to physicians in quintile 1 (best performance) in a given measure to patients attributed to physicians in each of the subsequent quintiles in the same measure. At the patient level, because a given measure's output could be expressed as a binary outcome (e.g. for a patient with new hip or knee osteoarthritis attributed to an orthopedic surgeon, whether the patient received arthroscopic surgery) or a count-based outcome (e.g. for a patient with stable chronic coronary artery disease attributed to a cardiologist, the number of stress tests received), the model used a logit or linear functional form.

$$Y_{ijk} = \alpha + \rho DxCG_i + \delta SES_i + \beta Quintile_{jk} + \varepsilon_{ijk}$$

In this model, Y_{ijk} denotes performance on a given measure for patient *i,* who is attributed to physician *j,* in metropolitan statistical area *k*. DxCG denotes the DxCG risk score, which uses age, sex, and clinical diagnoses to derive a measure that reflects expected spending and is often

used by insurers for risk adjustment. *SES* denotes a composite score of social determinants of health derived from seven U.S. Census variables based on the patient's zip code of residence, according to the methodology of the Agency for Healthcare Research and Quality.[1] These seven variables were: median household income, percent with less than high school graduation among populations 25 years and over, percent with bachelor's degree or higher among populations 25 years and over, percent of families below the poverty level among all people, civilian labor force unemployment rate, median value of owner-occupied units, and percentage of households containing one or more person per room. *Quintile* denotes a binary variable indicating the quintile of performance of an attributed physician (quintiles 2-5) relative to the reference quintile (quintile 1) for a given measure in a metropolitan statistical area. Within each measure in each metropolitan statistical area, quintile 1 was defined as physicians whose performance reflected the most favorable end of the distribution—denoting more appropriate or guideline-concordant care on average—relative to the subsequent quintiles 2-5. This produced the coefficient of interest, which captured the magnitude of the difference in mean performance between quintile 1 physicians and physicians in one of the subsequent quintiles. Standard errors were clustered at the physician level. Due to the large number of comparisons in the study across the 14 measures and 5 metropolitan statistical areas, each of which we took to be of equal importance relative to the others (in other words, we did not have a primary outcome), we did not conduct individual statistical tests for each difference in mean performance. We used 95% confidence intervals to convey the uncertainty around mean differences in performance.

eTables 1-7 show results from the base model and results from sensitivity analyses. Model 1 results are the main estimates. Sensitivity analyses tested the robustness of the main estimates to alterations in the model, focusing on the role of clinical risk and socioeconomic status adjustment. Model 2 omitted clustered standard errors. Model 3 omitted the DxCG risk score. Model 4 omitted the SES score. Model 5 omitted both the DxCG and SES scores. Stable estimates among sensitivity analyses relative to Model 1 would suggest that any observable differences in patient age, sex, clinical diagnoses, and socioeconomic status characteristics across physician quintiles contributed minimal bias toward the differences in performance between the quintiles. However, they would not adjust for all potential confounders. The interpretation of the regression coefficients varies by measure (e.g. average differences in utilization or percentage point differences in measure performance between quintiles).

[1] Agency for Healthcare Research and Quality. Creating and Validating an Index of Socioeconomic Status, in Creation of New Race-Ethnicity Codes and SES Indicators for Medicare Beneficiaries - Chapter 3. Rockville, MD. 2008 Jan. (http://archive.ahrq.gov/research/findings/final-reports/medicareindicators/medicareindicators3.html)

eMethods 3. Measures by Specialty and Number of Patients per Physician by Measure*

	Southeast MSA 1	Southeast MSA 2	South Central MSA	Midwest MSA	West MSA
	Average No. of Patients per Physician in the Measure (25th percentile – 75th percentile)				
**Cardiologists**					
Stress tests in patients with stable chronic CAD (per 100 patient years)	162 (68-200)	153 (74-200)	239 (100-338)	173 (71-251)	148 (54-212)
Statin therapy in patients with chronic CAD (%)	169 (67-214)	149 (69-191)	161 (62-224)	117 (44-167)	87 (35-110)
**Endocrinologists**					
Kidney function test in patients with diabetes (%)	271 (161-357)	261 (103-375)	570 (172-870)	388 (143-562)	319 (193-404)
Oral glucose-lowering medication in patients with diabetes (%)	279 (145-356)	248 (91-342)	355 (97-534)	240 (80-357)	165 (87-219)
**Gastroenterologists**					
Polyp detected on screening colonoscopy (%)	202 (90-302)	192 (72-248)	399 (163-566)	325 (82-451)	234 (109-318)
Endoscopy in patients with GERD and no alarm symptoms (%)	272 (108-339)	215 (97-300)	549 (270-766)	383 (145-533)	239 (99-348)
**Pulmonologists**					
Bronchodilator in patients with COPD (%)	70 (31-86)	56 (20-68)	54 (24-68)	41 (19-51)	26 (17-31)
Spirometry in patients with COPD (%)	87 (39-110)	70 (26-92)	118 (47-166)	98 (36-130)	76 (34-92)
**Obstetricians**					
Appropriate prenatal screening in pregnant patients (%)	43 (17-53)	33 (14-35)	269 (196-250)	167 (114-190)	50 (18-64)
Caesarean delivery in patients with low-risk pregnancies (%)	49 (20-60)	45 (23-51)	114 (50-156)	90 (36-119)	64 (23-87)
**Orthopedic Surgeons (Joint)**					
Any PT prior to elective hip or knee replacement (%)	44 (19-52)	55 (19-69)	91 (26-109)	86 (24-101)	72 (24-93)
Arthroscopy in patients with new hip or knee osteoarthritis (%)	43 (21-54)	46 (20-67)	93 (32-126)	92 (38-129)	54 (29-70)
**Orthopedic Surgeons and Neurosurgeons (Spine)**					
Spinal fusion in patients with low back pain (%)	40 (20-49)	34 (18-39)	99 (37-131)	70 (27-93)	55 (26-71)
Any PT in patients with new cervical spine pain (%)	30 (16-40)	27 (14-32)	61 (20-80)	41 (18-52)	31 (17-38)

* CAD = coronary artery disease; GERD = gastroesophageal reflux disease; COPD = chronic obstructive pulmonary disease; PT = physical therapy.

© 2022 Song Z et al. *JAMA Health Forum.*

eFigure 1. Physician-Level Variation in Cardiologist Coronary Artery Disease Care

Stress tests in patients with stable chronic CAD (per 100 patient years)*

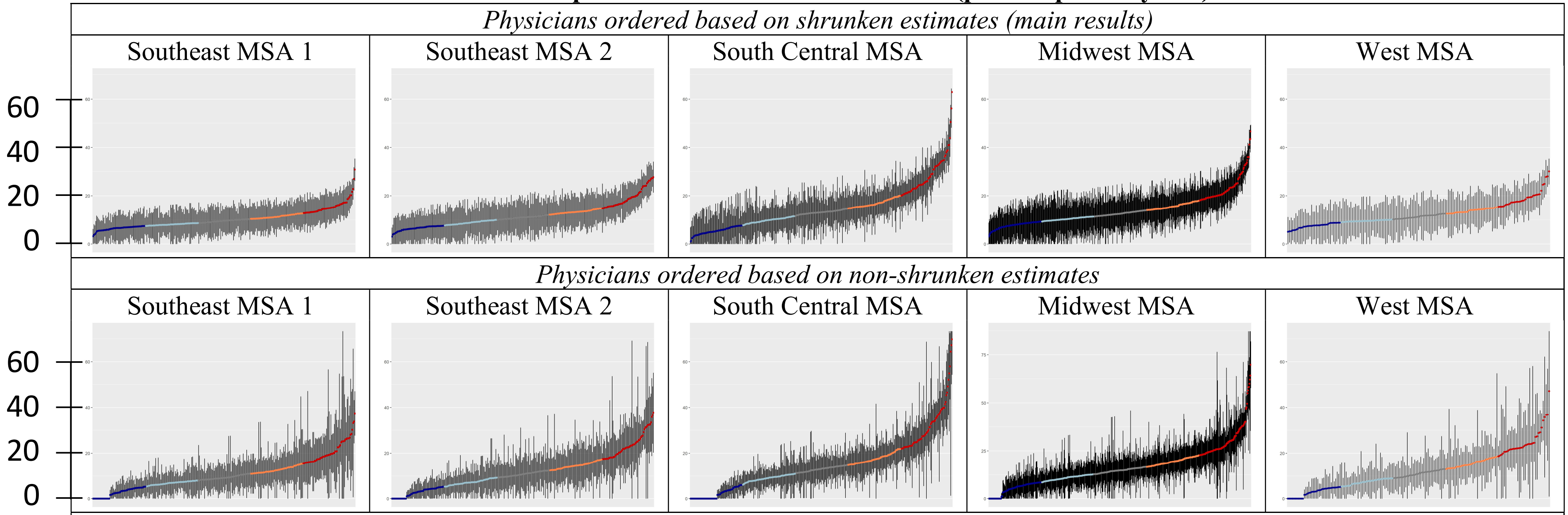

Average measure reliability (%)				
72.2	75.9	87.1	79.6	73.0
Proportion of physicians with reliability above 70% (%)				
60.8	75.7	94.0	82.5	64.7

* Denominator: patients with stable chronic coronary artery disease (CAD). Stable chronic CAD is defined as a patient with CAD for at least 6 months and had no ED visits for chest pain, office visits for chest pain, or admissions for AMI within the last 30 days. Numerator: patients in the denominator who had a cardiac stress test. Exclusions: patient enrollment time containing any ED visit for chest pain, office visit for chest pain, or admission for AMI within the last 30 days. A lower quantity of testing denotes more favorable performance. Attribution was to the cardiologist with whom a patient had the most office visits within 3 years. Measure validity is based on the specifications of the Centers for Medicare and Medicaid Services and National Quality Forum measure: Cardiac Stress Imaging Not Meeting Appropriate Use Criteria: Testing in Asymptomatic, Low-Risk Patients (NQF ID: 0672).[2]

[2] Centers for Medicare and Medicaid Services. Measures Inventory Tool. Cardiac Stress Imaging Not Meeting Appropriate Use Criteria: Testing in Asymptomatic, Low-Risk Patients. (https://cmit.cms.gov/CMIT_public/ViewMeasure?MeasureId=1856)

© 2022 Song Z et al. *JAMA Health Forum.*

Statin therapy in patients with chronic coronary artery disease (CAD) (%)*

Physicians ordered based on shrunken estimates (main results)

Southeast MSA 1 | Southeast MSA 2 | South Central MSA | Midwest MSA | West MSA

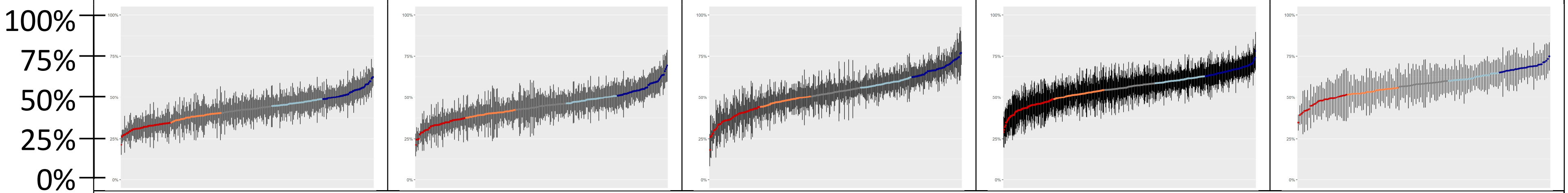

Physicians ordered based on non-shrunken estimates

Southeast MSA 1 | Southeast MSA 2 | South Central MSA | Midwest MSA | West MSA

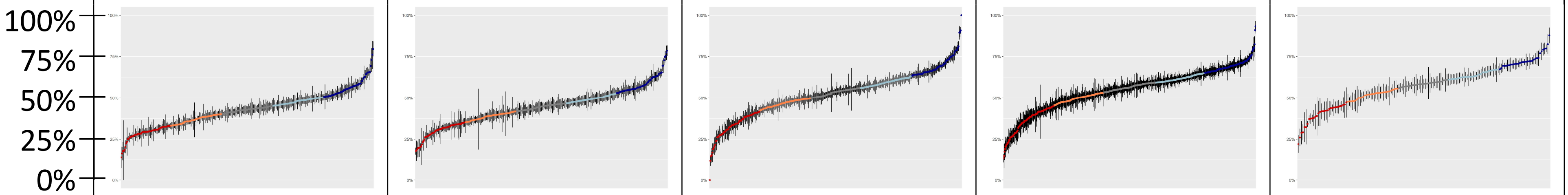

Southeast MSA 1	Southeast MSA 2	South Central MSA	Midwest MSA	West MSA
Average measure reliability (%)				
84.0	84.5	88.3	83.6	79.1
Proportion of physicians with reliability above 70% (%)				
89.9	89.9	96.2	88.6	80.4

* Denominator: patients with chronic coronary artery disease (CAD) who have prescription drug coverage in their insurance plan. Chronic CAD is defined as a patient with CAD for at least 6 months. Numerator: patients in the denominator who had a medication fill for a statin. Exclusions: members for whom we could not observe a diagnosis of CAD earlier than 6 months prior to the statin fill. A higher proportion denotes more favorable performance. Attribution was to the cardiologist with whom the patient had the most office visits within 3 years. Measure validity is based on the specifications of the National Committee for Quality Assurance measure: Statin Therapy for Patients with Cardiovascular Disease.[3]

[3] National Committee for Quality Assurance. Statin Therapy for Patients with Cardiovascular Disease and Diabetes (SPC/SPD). (https://www.ncqa.org/hedis/measures/statin-therapy-for-patients-with-cardiovascular-disease-and-diabetes/)

© 2022 Song Z et al. *JAMA Health Forum.*

eFigure 2. Physician-Level Variation in Endocrinologist Diabetes Care

Kidney function test in patients with diabetes (%)*

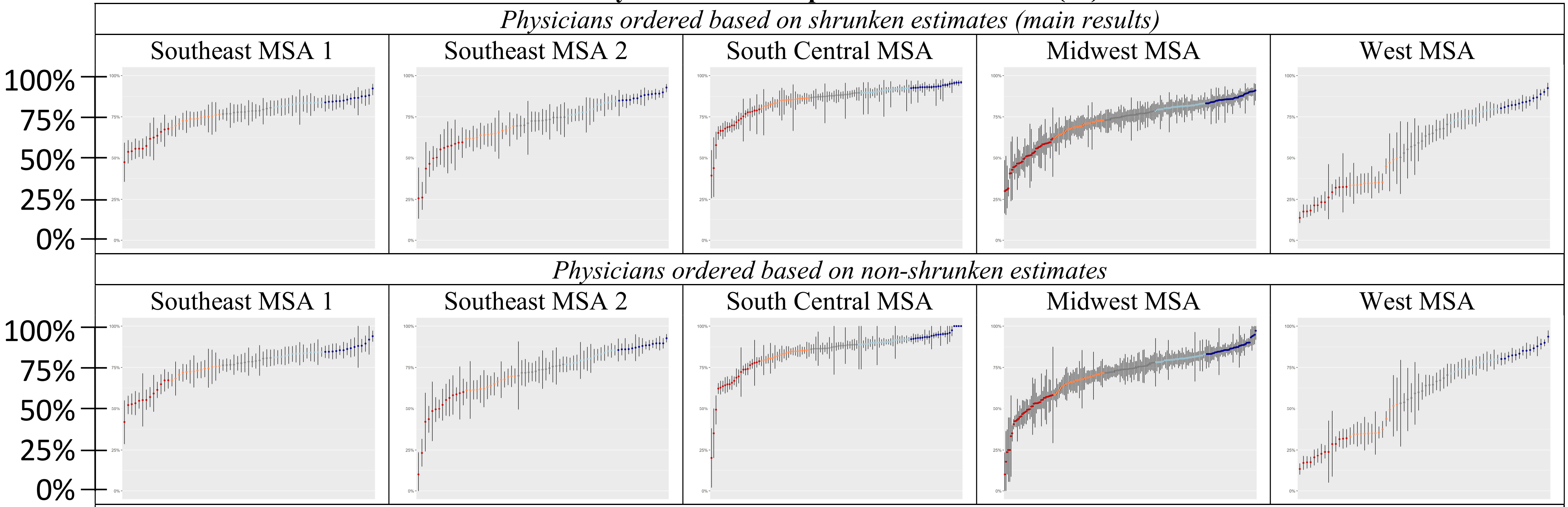

Average measure reliability (%)				
90.8	93.6	91.7	93.6	97.4
Proportion of physicians with reliability above 70% (%)				
97.1	100.0	91.9	99.5	100.0

* Denominator: patients with diabetes aged 18-75 years and see an endocrinologist. Diabetes is defined as a diagnosis of type 1 or type 2 diabetes within the past 5 years. Numerator: patients in the denominator who received a kidney function test in a year (annual measure). Exclusions: patients with hospice care. Required continuous enrollment in each year eligible for measurement. A higher proportion denotes more favorable performance. Attribution was to the endocrinologist with whom the patient had the most office visits within 3 years. Measure validity is based on the specifications of the Centers for Medicare and Medicaid Services and National Quality Forum measure: Diabetes: Medical Attention for Nephropathy (NQF ID: 0062).[4]

[4] Centers for Medicare and Medicaid Services. Measures Inventory Tool. Diabetes: Medical Attention for Nephropathy. (https://cmit.cms.gov/CMIT_public/ViewMeasure?MeasureId=1406)

© 2022 Song Z et al. *JAMA Health Forum.*

Oral glucose-lowering medication in patients with diabetes (%)*

Physicians ordered based on shrunken estimates (main results)

Southeast MSA 1	Southeast MSA 2	South Central MSA	Midwest MSA	West MSA

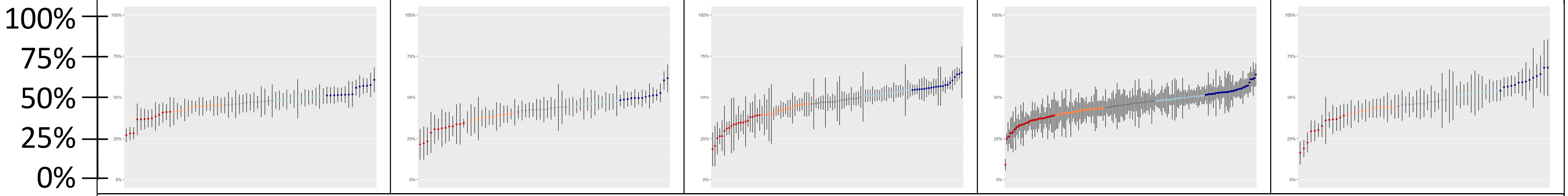

Physicians ordered based on non-shrunken estimates

Southeast MSA 1	Southeast MSA 2	South Central MSA	Midwest MSA	West MSA

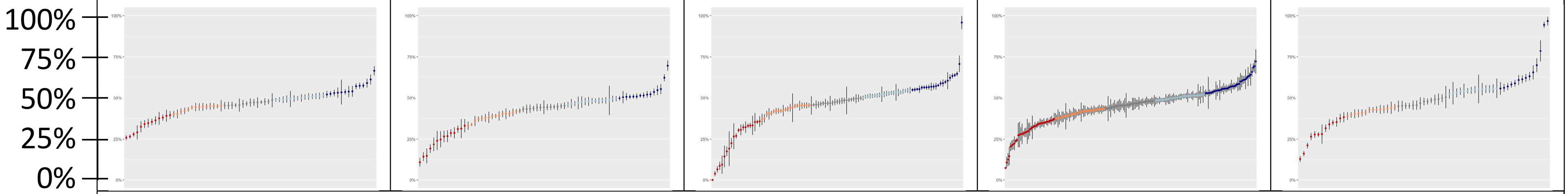

Southeast MSA 1	Southeast MSA 2	South Central MSA	Midwest MSA	West MSA
Average measure reliability (%)				
85.7	86.9	88.6	85.0	88.8
Proportion of physicians with reliability above 70% (%)				
94.2	92.8	87.9	86.2	92.8

* Denominator: patients 18 years of age and older with diabetes who have prescription drug coverage in their insurance plan and see an endocrinologist. Diabetes is defined as a diagnosis of type 1 or type 2 diabetes within the past 5 years. Numerator: patients in the denominator who filled a prescription for an oral glucose-lowering agent. Oral glucose-lowering agents include metformin, sulfonylureas, thiazolidinediones, biguanides, bile acid agents, newer agents such as the sodium-glucose cotransporter-2 (SGLT-2) inhibitors, and others. A higher proportion denotes more favorable performance. Attribution was to the endocrinologist with whom the patient had the most office visits within 3 years. Measure validity borrows from the specifications of the Centers for Medicare and Medicaid Services and National Quality Forum measure: Medication Adherence for Diabetes Medications (NQF ID: 0541).[5]

[5] Centers for Medicare and Medicaid Services. Measures Inventory Tool. Medication Adherence for Diabetes Medications. (https://cmit.cms.gov/CMIT_public/ViewMeasure?MeasureId=4075)

© 2022 Song Z et al. *JAMA Health Forum.*

eFigure 3. Physician-Level Variation in Gastroenterologist Gastrointestinal Tract Care

Polyp detected on screening colonoscopy (%)*

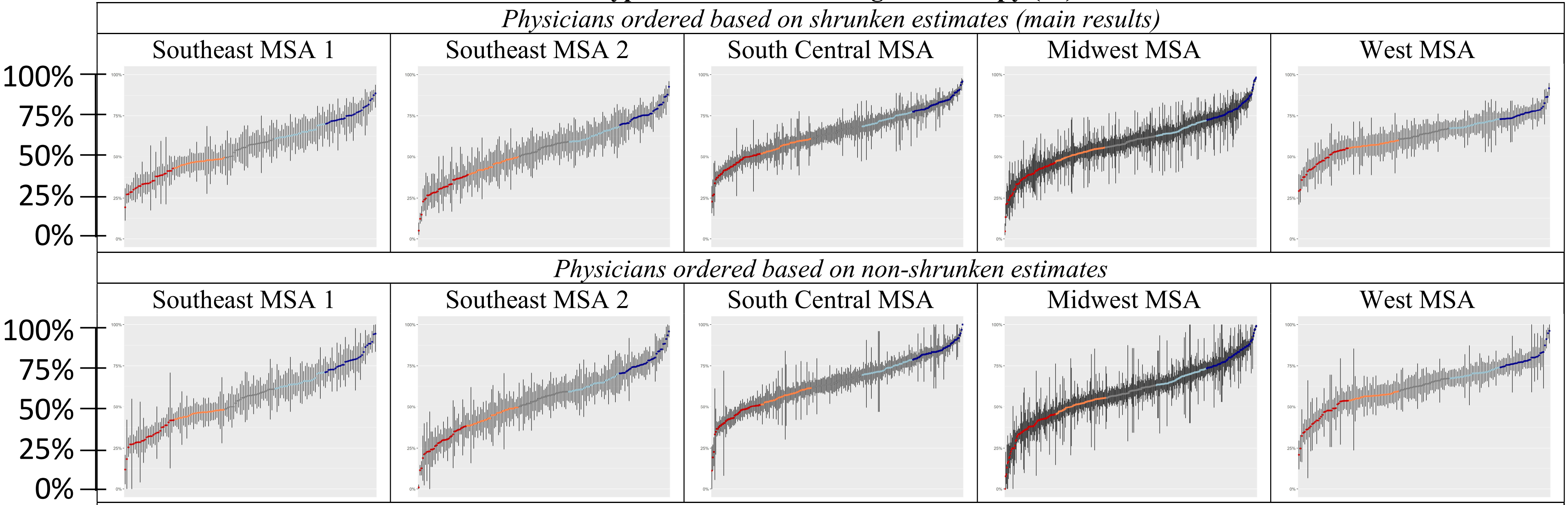

Southeast MSA 1	Southeast MSA 2	South Central MSA	Midwest MSA	West MSA
Average measure reliability (%)				
92.5	93.7	94.7	93.7	90.7
Proportion of physicians with reliability above 70% (%)				
98.6	100.0	98.4	99.5	96.5

* Denominator: patients 50 years of age or older who had a screening colonoscopy and had at least one documented risk factor for colorectal cancer (e.g. family history of colonic polyps, family history of malignant neoplasm of digestive organs, and encounter for screening for malignant neoplasm of colon). A screening colonoscopy is performed for colorectal cancer prevention and is distinct from a diagnostic colonoscopy, which is often performed during a diagnostic workup such as in response to a gastrointestinal bleed or other signs and symptoms. Numerator: patients in the denominator whose colonoscopy revealed a polyp, whether it was benign, an adenoma, or a colorectal cancer. A higher proportion denotes more favorable performance. Attribution was to the gastroenterologist who performed the screening colonoscopy. Measure validity relies on the specifications of the Centers for Medicare and Medicaid Services and National Quality Forum measure Quality ID #343: Screening Colonoscopy Adenoma Detection Rate.[6]

[6] Centers for Medicare and Medicaid Services. Quality ID #343. Screening Colonoscopy Adenoma Detection Rate. (https://qpp.cms.gov/docs/QPP_quality_measure_specifications/CQM-Measures/2019_Measure_343_MIPSCQM.pdf)

© 2022 Song Z et al. *JAMA Health Forum.*

Endoscopy in patients with gastroesophageal reflux disease (GERD) and no alarm symptoms (%)*

Physicians ordered based on shrunken estimates (main results)

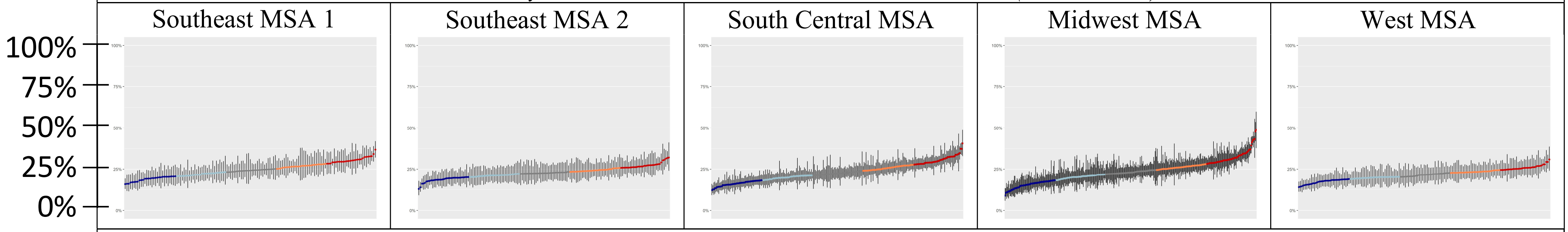

Physicians ordered based on non-shrunken estimates

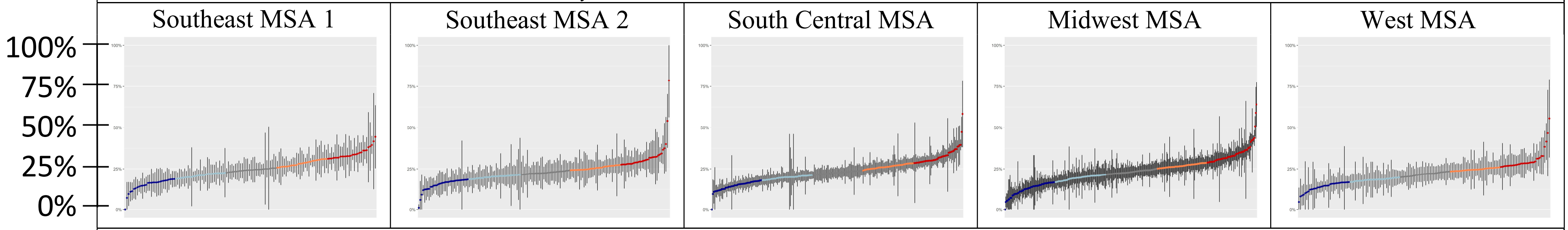

Southeast MSA 1	Southeast MSA 2	South Central MSA	Midwest MSA	West MSA
Average measure reliability (%)				
78.2	72.7	87.4	86.8	74.3
Proportion of physicians with reliability above 70% (%)				
77.3	60.1	92.1	91.2	67.1

* Denominator: patients who had a diagnosis of GERD in the past 5 years and who has had no alarm symptoms. Alarm symptoms include weight loss, anemia, bleeding, dysphagia, and odynophagia and render endoscopy more appropriate in the setting of GERD. Numerator: patients in the denominator who had an upper endoscopy during the measurement period. Exclusions: a diagnosis of GERD greater than 5 years prior; presence of alarm symptoms. Continuous enrollment required. A lower proportion denotes more favorable performance. Attribution was to the gastroenterologist with whom a patient had the most office visits within 3 years. Given the apparent absence of such a measure in the public inventory, this measure is novel. Measure validity relies on the practice guidelines and clinical evidence that indicate, on average, endoscopy in patients with GERD and no alarm symptoms is less appropriate, as noted in Methods S1.

© 2022 Song Z et al. *JAMA Health Forum.*

eFigure 4. Physician-Level Variation in Pulmonologist COPD Care

Spirometry in patients with chronic obstructive pulmonary disease (COPD)*

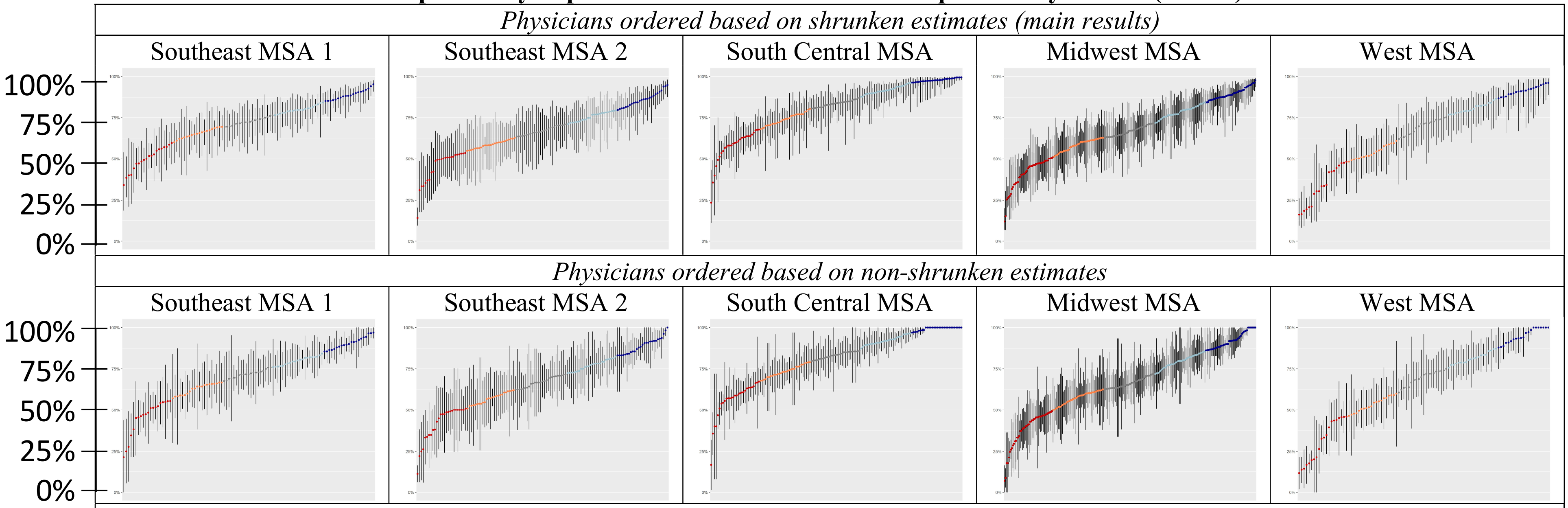

Average measure reliability (%)				
88.2	87.3	91.5	91.2	91.9
Proportion of physicians with reliability above 70% (%)				
99.0	99.2	99.3	98.7	100.0

* Denominator: patients with COPD. Numerator: patients in the denominator who have had any spirometry. Continuous enrollment required. A higher proportion denotes more favorable performance. Attribution was to the pulmonologist with whom the patient had the most office visits within 3 years. Measure validity relies on the specifications of the Centers for Medicare and Medicaid Services and National Quality Forum measure Quality ID #51: Chronic Obstructive Pulmonary Disease (COPD): Spirometry Evaluation (NQF ID: 0091),[7] and the National Committee for Quality Assurance measure: Use of Spirometry Testing in the Assessment and Diagnosis of COPD.[8]

---

[7] Centers for Medicare and Medicaid Services. Quality ID #51 (NQF 0091): Chronic Obstructive Pulmonary Disease (COPD): Spirometry Evaluation. (https://qpp.cms.gov/docs/QPP_quality_measure_specifications/CQM-Measures/2019_Measure_051_MIPSCQM.pdf)

[8] National Committee for Quality Assurance. Use of Spirometry Testing in the Assessment and Diagnosis of COPD (SPR). (https://www.ncqa.org/hedis/measures/use-of-spirometry-testing-in-the-assessment-and-diagnosis-of-copd/)

© 2022 Song Z et al. *JAMA Health Forum.*

Bronchodilator in patients with chronic obstructive pulmonary disease (COPD) (%)*

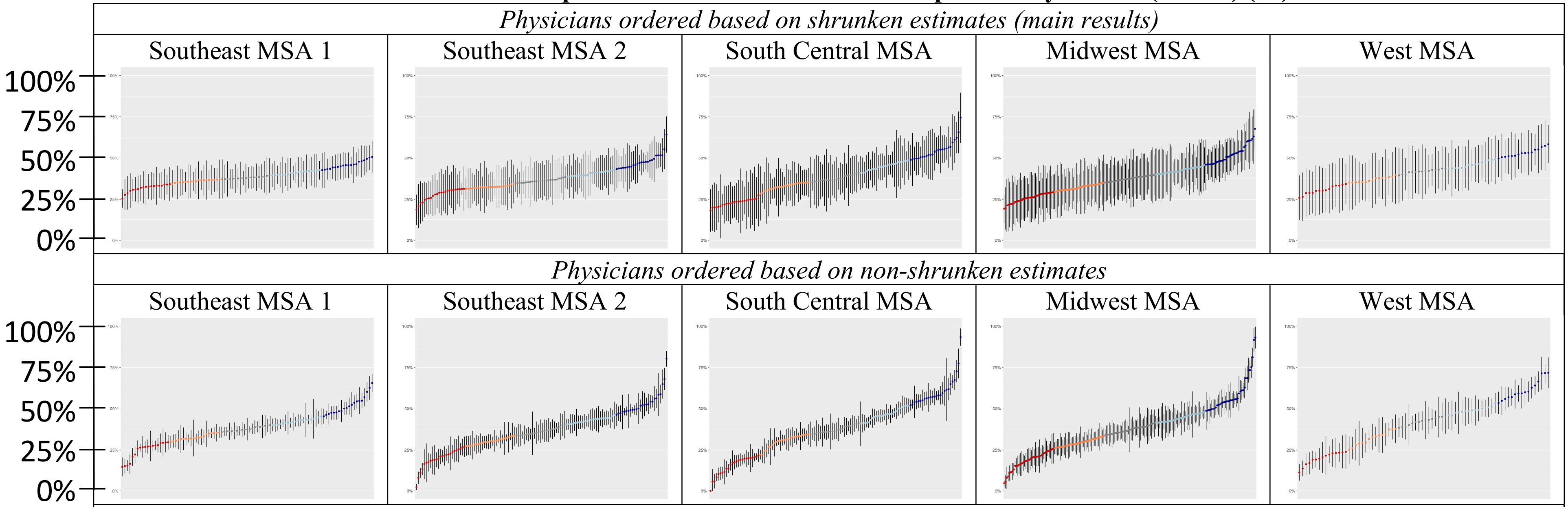

Southeast MSA 1	Southeast MSA 2	South Central MSA	Midwest MSA	West MSA
Average measure reliability (%)				
71.8	75.3	83.3	78.0	73.1
Proportion of physicians with reliability above 70% (%)				
58.3	68.9	91.3	79.5	67.1

* Denominator: patients older than 40 years and under than 65 years of age with COPD and prescription drug coverage in their insurance plan. Numerator: patients in the denominator who had a medication fill for a bronchodilator, which included anticholinergic agents, beta-2-agonists, methylxanthines, and related agents. A higher proportion denotes more favorable performance. Attribution was to the pulmonologist with whom the patient had the most office visits within 3 years. Measure validity relies on the specifications of the Centers for Medicare and Medicaid Services and National Quality Forum measure: Chronic Obstructive Pulmonary Disease (COPD): Long-Acting Inhaled Bronchodilator Therapy (NQF ID: 0102).[9]

[9] Centers for Medicare and Medicaid Services. Measures Inventory Tool. Chronic Obstructive Pulmonary Disease (COPD): Long-Acting Inhaled Bronchodilator Therapy. (https://cmit.cms.gov/CMIT_public/ViewMeasure?MeasureId=328)

© 2022 Song Z et al. *JAMA Health Forum.*

eFigure 5. Physician-Level Variation in Obstetrician Prenatal and Delivery Care

Appropriate prenatal screening in pregnant patients (%)*

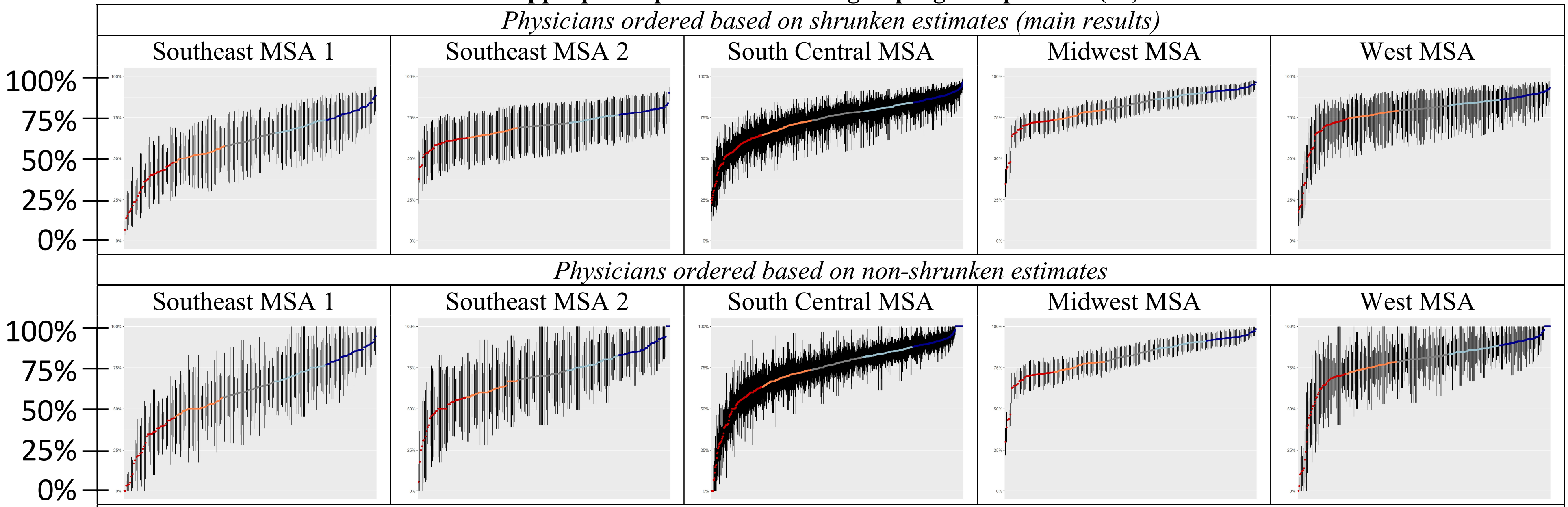

Southeast MSA 1	Southeast MSA 2	South Central MSA	Midwest MSA	West MSA
Average measure reliability (%)				
84.7	71.1	86.5	90.9	79.7
Proportion of physicians with reliability above 70% (%)				
100.0	46.1	95.0	100.0	81.2

* Denominator: low risk pregnancies defined as the absence of multiple gestation; maternal endocrine, gastrointestinal, or cardiovascular conditions (either pre-existing or gestational); conditions of the placenta, amniotic fluid, and uterus; and fetal conditions including fetal abnormalities, fetal demise, and reduced fetal movement or growth. Required minimum 8 months of continuous enrollment. Numerator: pregnancies in the denominator that received the following recommended prenatal tests prior to delivery: Rh factor, glucose tolerance test, group B strep, and urinalysis. A higher proportion denotes more favorable performance. Attribution was to the obstetrician with whom the patient had the most visits, prior to surgery or delivery if applicable. Measure validity is derived from the Maternity Care Performance Measurement Set of the American Congress of Obstetricians and Gynecologists, National Committee for Quality Assurance, and Physician Consortium for Performance Improvement.[10] Additional guidance was derived from the Centers for Medicare and Medicaid Services and National Quality Forum measure: Prenatal and Postpartum Care: Timeliness of Prenatal Care (NQF ID: 1517); the NQF endorsement for this particular measure has since been removed.[11]

---

[10] American Congress of Obstetricians and Gynecologists, National Committee for Quality Assurance, and Physician Consortium for Performance Improvement. Maternity Care Performance Measurement Set. 2012 Mar 17. (https://www.ahrq.gov/sites/default/files/wysiwyg/CHIPRA-BMI-Maternity-Care-Measures.pdf)
[11] Centers for Medicare and Medicaid Services. Measures Inventory Tool. Prenatal and Postpartum Care: Timeliness of Prenatal Care. (https://cmit.cms.gov/CMIT_public/ViewMeasure?MeasureId=5894)

© 2022 Song Z et al. *JAMA Health Forum.*

Caesarean delivery in patients with low-risk pregnancies (%)*

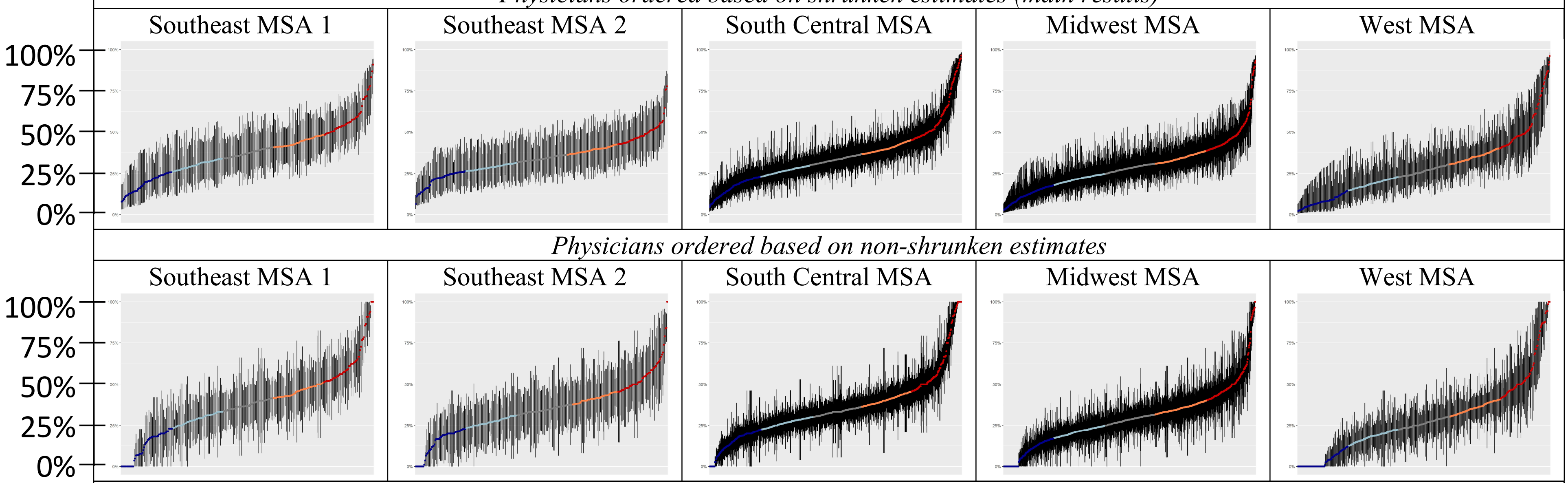

Average measure reliability (%)				
84.7	78.6	91.8	89.2	89.0
Proportion of physicians with reliability above 70% (%)				
95.3	83.3	99.1	97.8	96.3

* Denominator: uncomplicated deliveries defined as the absence of abnormal presentation, fetal death, or multiple gestation. Numerator: deliveries in the denominator performed by Cesarean delivery. A lower proportion denotes more favorable performance. Attribution was to the obstetrician who performed the delivery. This definition and the measure validity were derived from the specifications of the AHRQ measure on Cesarean deliveries in uncomplicated pregnancy.[12] Additional guidance was obtained from the Centers for Medicare and Medicaid Services and National Quality Forum measure on Cesarean Birth (NQF ID: 471).[13] Output correlated closely with a definition that additionally defined uncomplicated deliveries as the absence of HIV, cardiovascular disease, diabetes, kidney disease, pre-eclampsia, HELLP syndrome, alloimmunization, damage to fetus, poor fetal growth, oligo/polyhydramnios, placental abnormalities, uterine abnormalities, hemorrhage, complicated delivery, and post-term pregnancy.

---

[12] Agency for Healthcare Research and Quality. Inpatient Quality Indicator 21 (IQI 21) Cesarean Delivery Rate, Uncomplicated. 2017 Mar. (https://www.qualityindicators.ahrq.gov/Downloads/Modules/IQI/V60/TechSpecs/IQI_21_Cesarean_Delivery_Rate_Uncomplicated.pdf)

[13] Centers for Medicare and Medicaid Services. Measures Inventory Tool. PC-02: Cesarean Birth (PC02-CH). (https://cmit.cms.gov/CMIT_public/ViewMeasure?MeasureId=2831)

© 2022 Song Z et al. *JAMA Health Forum.*

eFigure 6. Physician-Level Variation in Orthopedist Joint Care

Any physical therapy (PT) prior to elective hip or knee replacement (%)*

Physicians ordered based on shrunken estimates (main results)

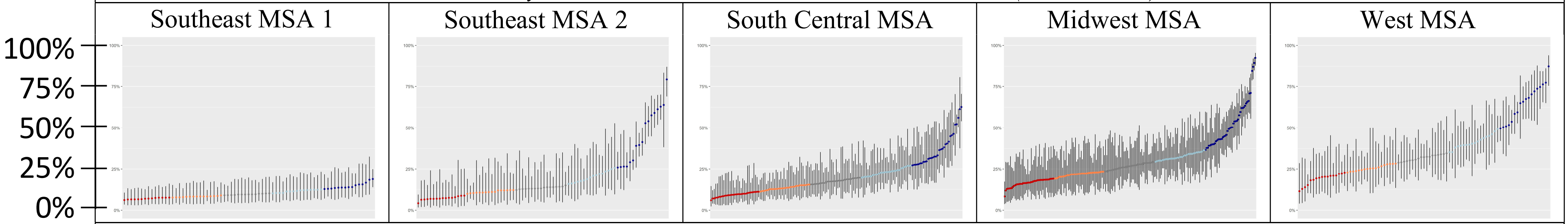

Physicians ordered based on non-shrunken estimates

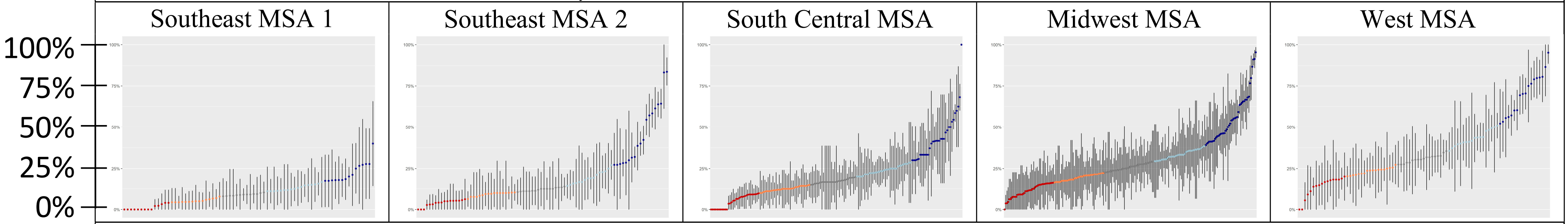

	Southeast MSA 1	Southeast MSA 2	South Central MSA	Midwest MSA	West MSA
Average measure reliability (%)	62.5	84.0	82.6	85.7	87.8
Proportion of physicians with reliability above 70% (%)	13.7	93.9	85.5	94.5	96.6

* Denominator: patients who had an elective hip or knee replacement. Elective is defined as an elective surgical admission in the absence of emergency care. Numerator: patients in the denominator who had physical therapy within 4 months before the procedure. A higher proportion denotes more favorable performance. Exclusions: transfers from one hospital to another hospital for surgery. Required 120 days (4 months) of continuous enrollment prior to surgery. Attribution was to the orthopedic surgeon with whom the patient had the most visits, prior to surgery if applicable. This definition and the measure validity were constructed from practice guidelines and the clinical literature, including those from the American Academy of Orthopaedic Surgeons, American Physical Therapy Association, and Agency for Healthcare Research and Quality that advise physical therapy prior to lower extremity joint replacement (see Methods S1 in this document). This measure was not adapted from a measure in the public domain, although its specification followed the model of other procedural measures in the public domain. Its validity is further enhanced by restricting to cases of elective joint replacement, for which the opportunity for at least one session of a physical therapy trial was plausibly available.

© 2022 Song Z et al. *JAMA Health Forum.*

Arthroscopy in patients with new hip or knee osteoarthritis (%)*

Physicians ordered based on shrunken estimates (main results)

Southeast MSA 1	Southeast MSA 2	South Central MSA	Midwest MSA	West MSA

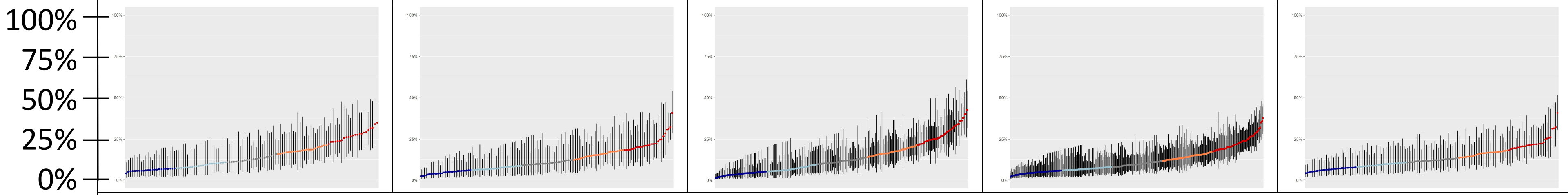

Physicians ordered based on non-shrunken estimates

Southeast MSA 1	Southeast MSA 2	South Central MSA	Midwest MSA	West MSA

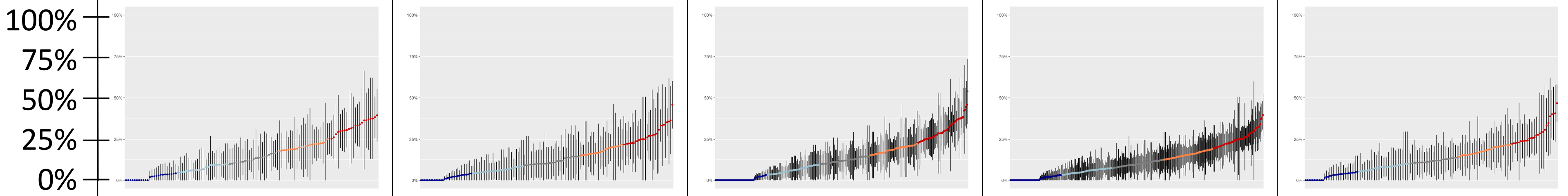

Average measure reliability (%)

Southeast MSA 1	Southeast MSA 2	South Central MSA	Midwest MSA	West MSA
76.6	76.5	84.8	81.4	75.7

Proportion of physicians with reliability above 70% (%)

Southeast MSA 1	Southeast MSA 2	South Central MSA	Midwest MSA	West MSA
70.4	70.1	86.8	79.2	72.1

* Denominator: patients 45 years or older with newly diagnosed hip or knee osteoarthritis. Numerator: patients in the denominator who had arthroscopic surgery within 1 year after the first diagnosis. A lower proportion denotes more favorable performance. Required 180 days of continuous enrollment prior to first diagnosis and 365 days following the first diagnosis. Attribution was to the orthopedic surgeon with whom the patient had the most visits, prior to arthroscopy if applicable. The measure definition derived from the clinical literature, including multiple randomized controlled trials and meta-analysis results, as well as practice guidelines resources such as UptoDate® that indicate no discernible benefit of arthroscopy for hip and knee osteoarthritis (see Methods S1 in this document). With regard to measure validity, this measure was not adapted from a measure in the public domain, although its specification followed the model of other procedural measures in the public domain.

© 2022 Song Z et al. *JAMA Health Forum.*

eFigure 7. Physician-Level Variation in Orthopedist/Neurosurgeon Spine Care

Spinal fusion in patients with low back pain (%)*

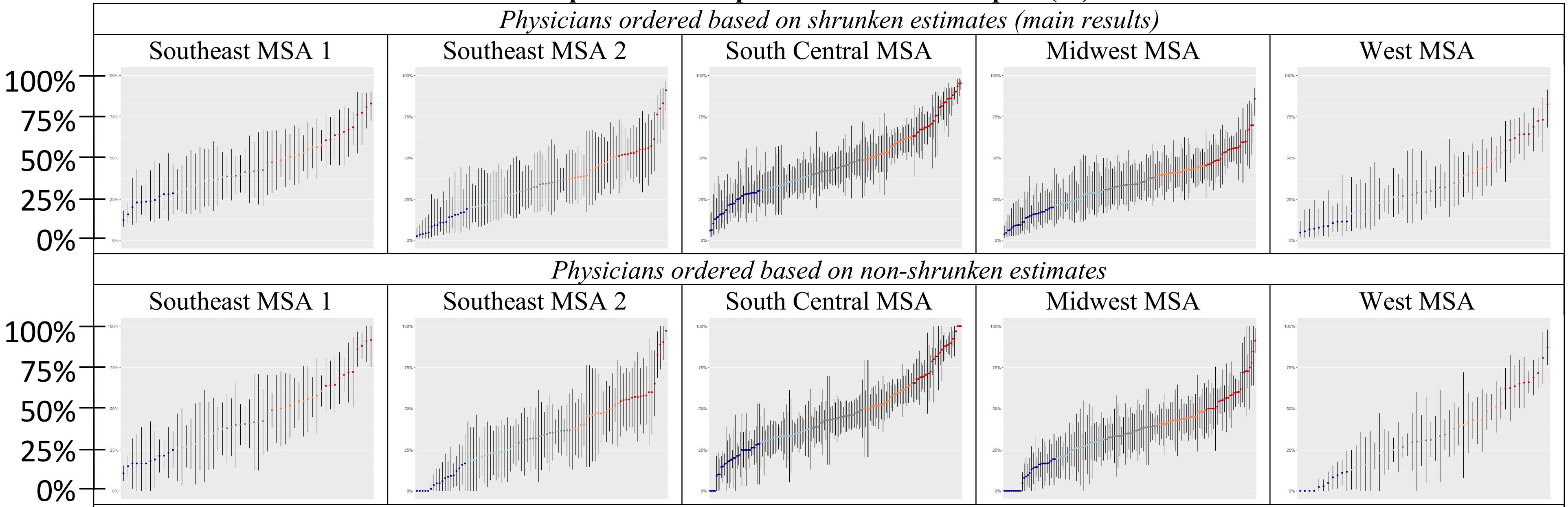

Southeast MSA 1	Southeast MSA 2	South Central MSA	Midwest MSA	West MSA
Average measure reliability (%)				
85.0	87.5	92.5	88.4	89.4
Proportion of physicians with reliability above 70% (%)				
100.0	100.0	100.0	97.9	98.1

* Denominator: patients with a diagnosis of low back pain (including radiculopathy, spondylolisthesis, spinal stenosis, panniculitis, sciatica, lumbago, muscle spasms, etc.) who underwent lumbar spinal surgery. Numerator: patients in the denominator who had a lumbar spinal fusion, as opposed to any other type of back surgery (e.g. decompression, diskectomy, laminectomy, etc.). A lower proportion denotes more favorable performance. Attribution was to the orthopedic surgeon or neurosurgeon who performed the surgery. The measure definition relied on the clinical literature, including multiple randomized controlled trials and meta-analyses, as well as practice guidelines resources such as UptoDate® that indicate little discernible benefit of spinal fusion for low back pain compared to physical therapy or other structured non-surgical management (see Methods S1 in this document). With regard to measure validity, this measure was not adapted from a measure in the public domain, although its specification followed the model of other procedural measures in the public domain.

© 2022 Song Z et al. *JAMA Health Forum.*

Any physical therapy (PT) in patients with new cervical spine pain (%)*

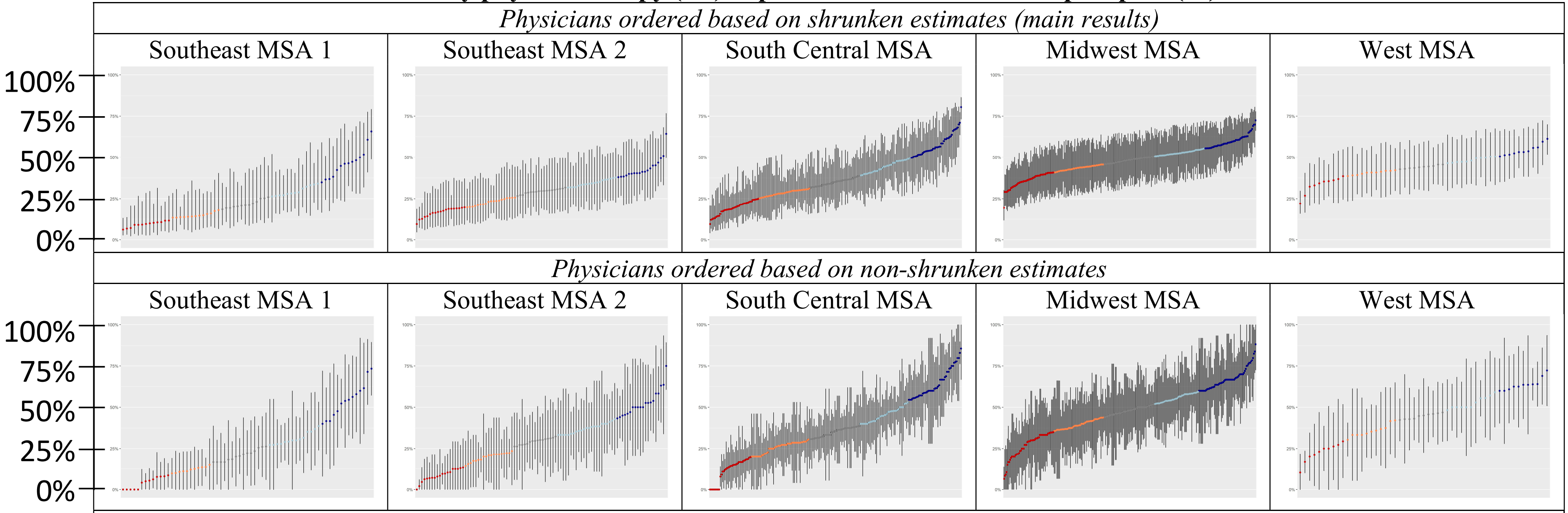

Southeast MSA 1	Southeast MSA 2	South Central MSA	Midwest MSA	West MSA
Average measure reliability (%)				
80.4	74.7	84.0	73.1	71.5
Proportion of physicians with reliability above 70% (%)				
90.9	67.4	91.7	56.6	52.8

* Denominator: patients 35 years or older with newly diagnosed cervical spine pain. Numerator: patients in the denominator who had at least one physical therapy encounter within 4 months of the first visit. Exclusions: patients who were seen by the surgeon more than 120 days after the initial diagnosis of cervical spine pain. Required 180 days of continuous enrollment prior to first diagnosis and 120 days (4 months) following the first diagnosis. A higher proportion denotes more favorable performance. Attribution was to the orthopedic surgeon or neurosurgeon with whom the patient had the most visits, prior to cervical spine procedures if applicable. The measure definition relied on the clinical literature, including practice guidelines that recommend physical therapy before surgery (see Methods S1 in this document).

© 2022 Song Z et al. *JAMA Health Forum.*

eFigure 8. Relationship Between Quality and Cost at the Physician Level

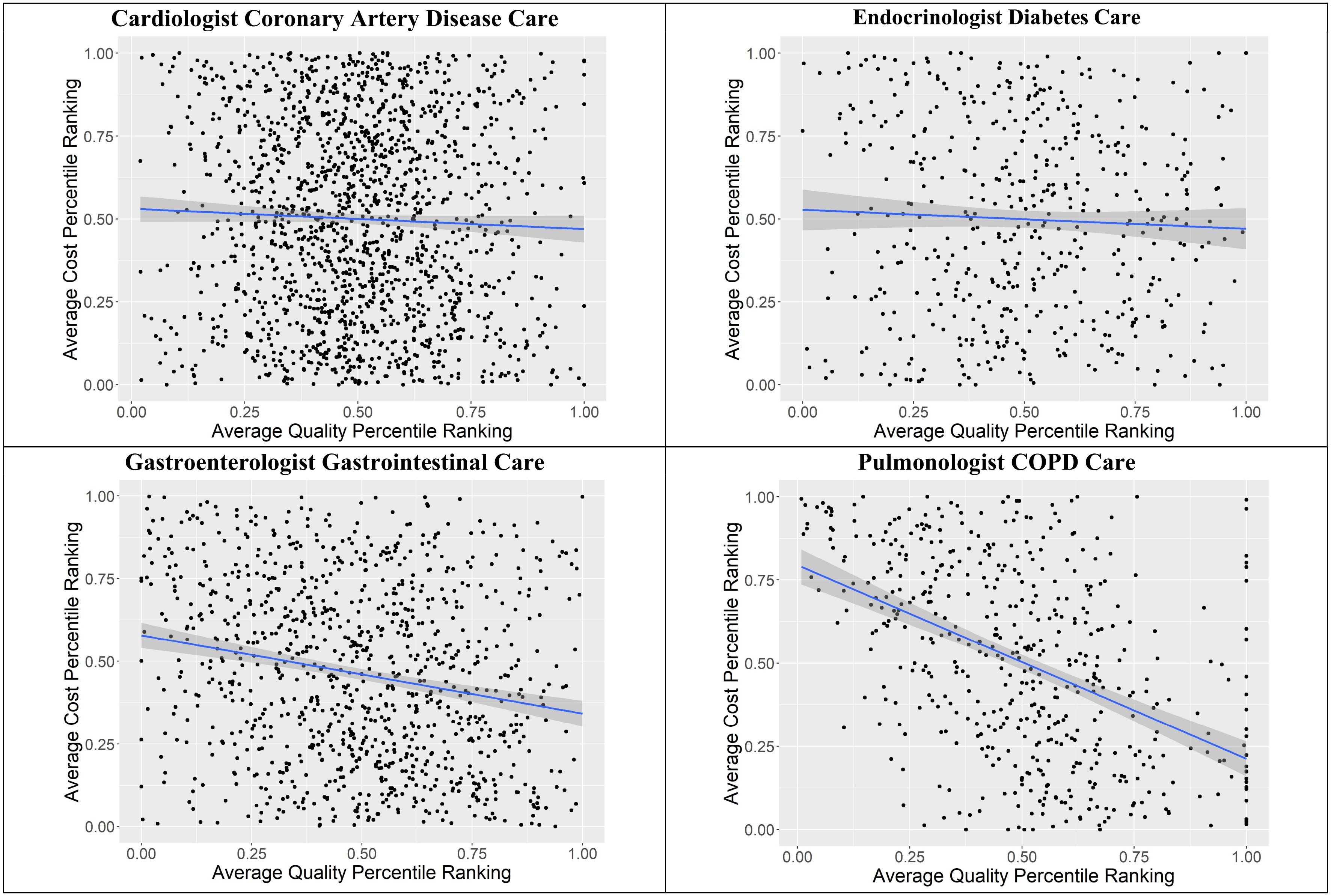

© 2022 Song Z et al. *JAMA Health Forum.*

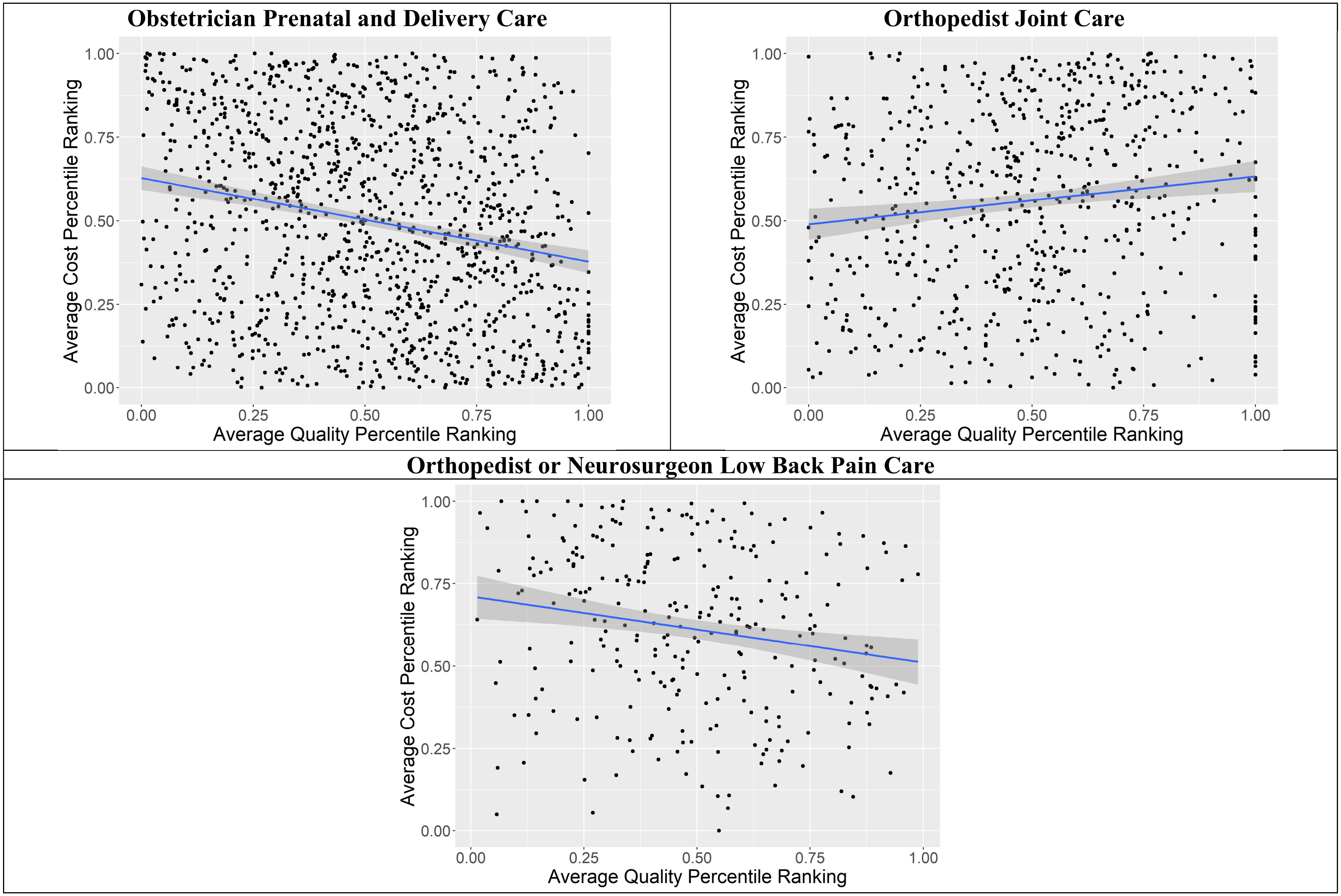

* Each data point is a physician. Each physician's unadjusted percentile of performance across measures is plotted vs. the percentile of corresponding episode-level spending. Within each clinical domain, physicians are pooled across all markets. An ordinary least squares best-fit line is overlayed.

© 2022 Song Z et al. *JAMA Health Forum.*

eFigure 9. Physician-Level Variations in Practice Patterns within Organizations*

Specialists (South Central MSA)	Share in Health System (%)
Cardiologists	84.8
Endocrinologists	57.9
Gastroenterologists	83.9
Pulmonologists	74.3
Obstetricians	81.9
Orthopedists (Joint)	85.3
Ortho/Neurosurgery (Spine)	81.4

Note: Data enabling linkages of physicians to their affiliated organization were available only for the South Central metropolitan statistical area (MSA). In this MSA, we were able to identify 4 health systems, each of which is a physician organization (labeled A through D in the graphs below). This table shows the proportion of specialists in each measure in this MSA who were affiliated with a health system.

Cardiologists

Stress tests in patients with stable chronic coronary artery disease (per 100 patient years)

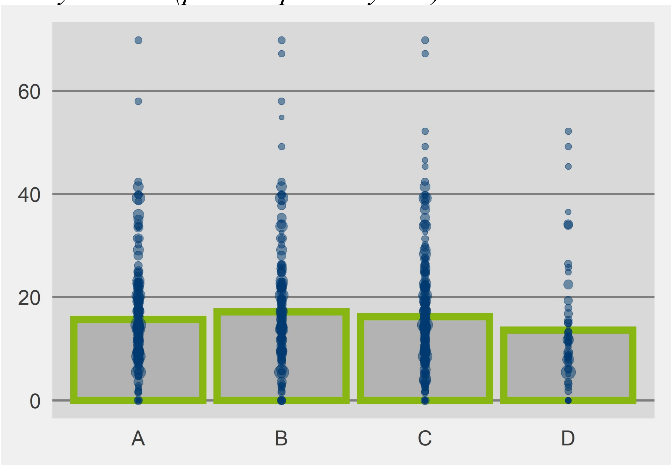

Statin therapy in patients with chronic coronary artery disease (%)

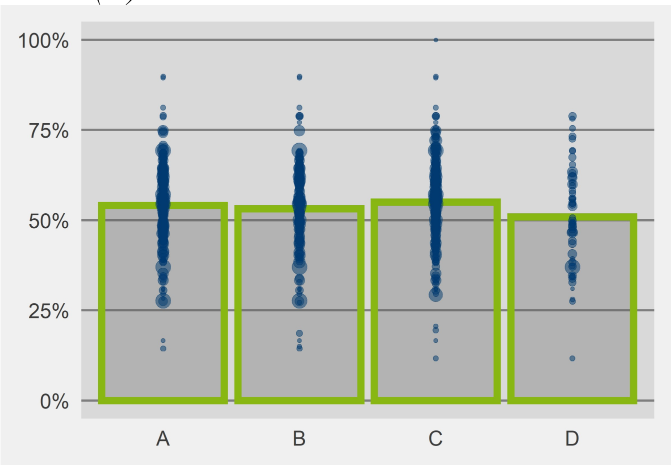

Endocrinologists

Kidney function test in patients with diabetes (%)

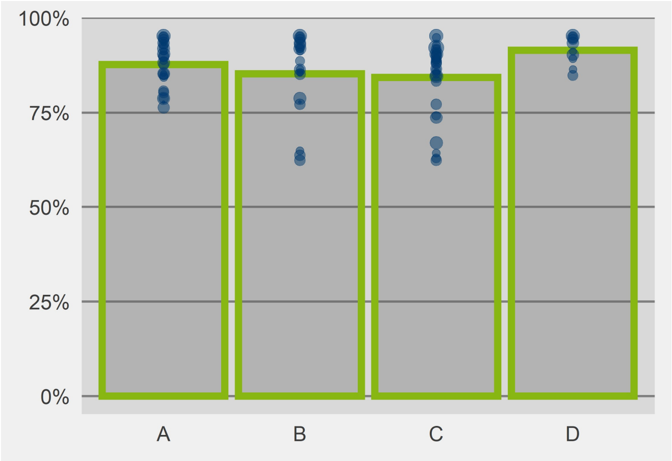

Oral glucose-lowering medication in patients with diabetes (%)

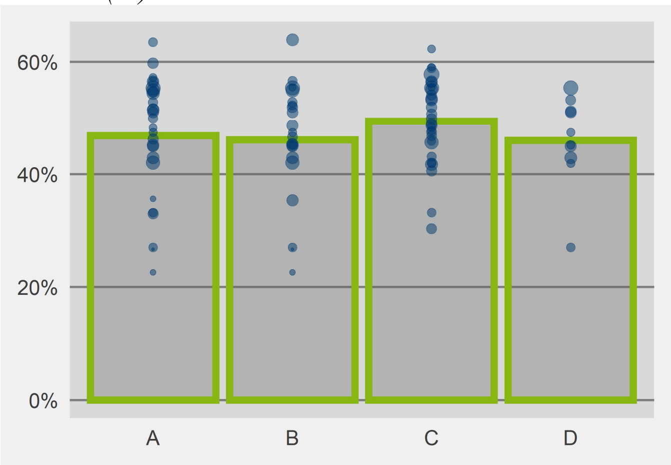

© 2022 Song Z et al. *JAMA Health Forum.*

Gastroenterologists

Polyp detected on screening colonoscopy (%)

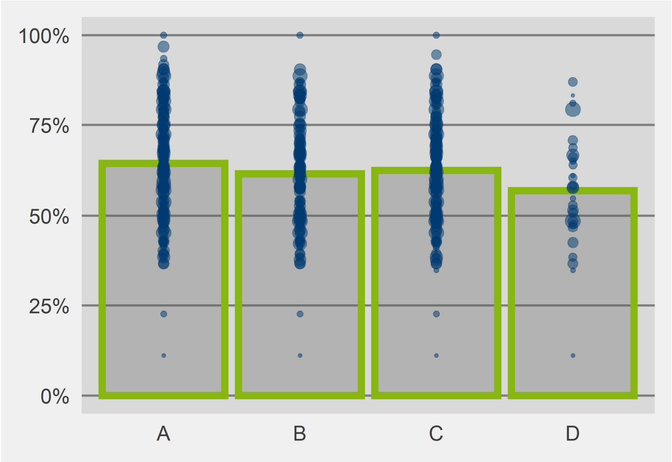

Endoscopy in patients with gastroesophageal reflux disease and no alarm symptoms (%)

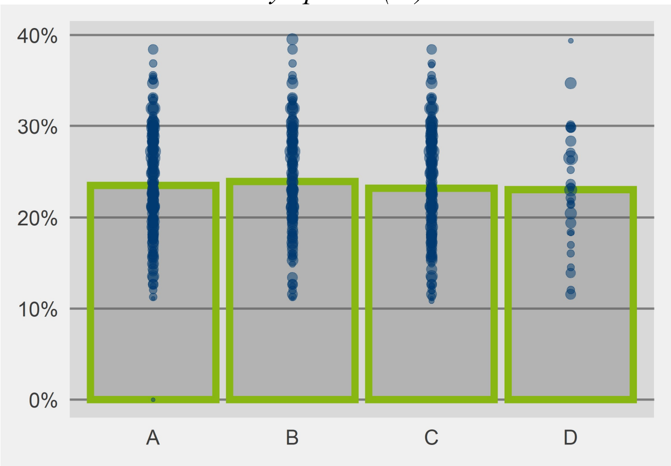

Pulmonologists

Bronchodilator in patients with chronic obstructive pulmonary disease (%)

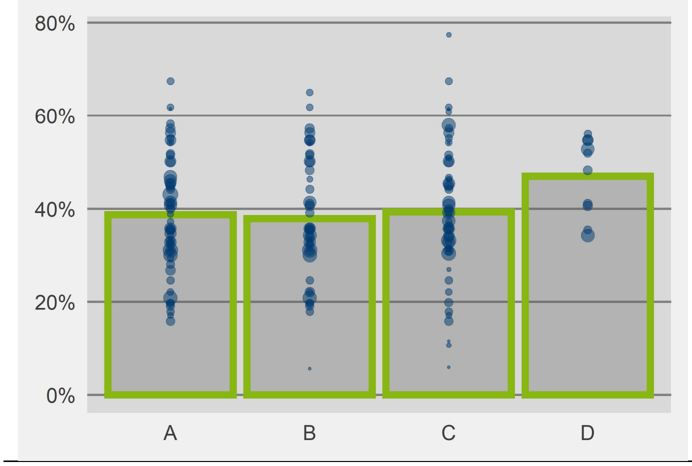

Spirometry in patients with chronic obstructive pulmonary disease (%)

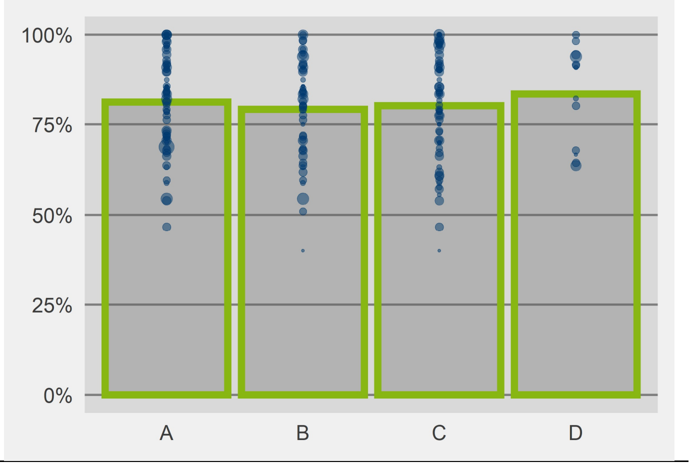

© 2022 Song Z et al. *JAMA Health Forum.*

Obstetricians

Appropriate prenatal screening in pregnant patients (%)

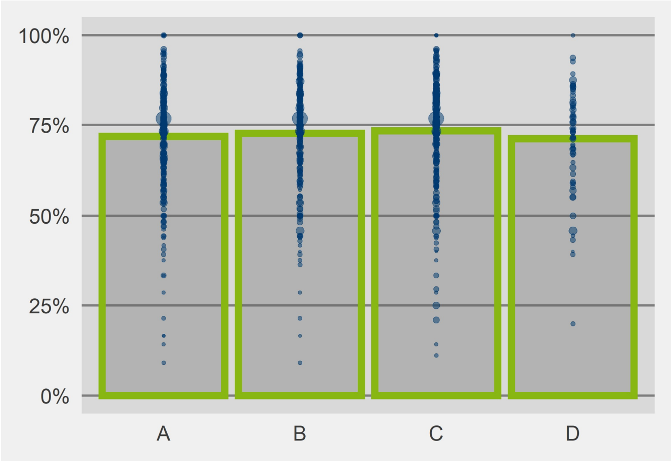

Caesarean delivery in patients with low-risk pregnancy (%)

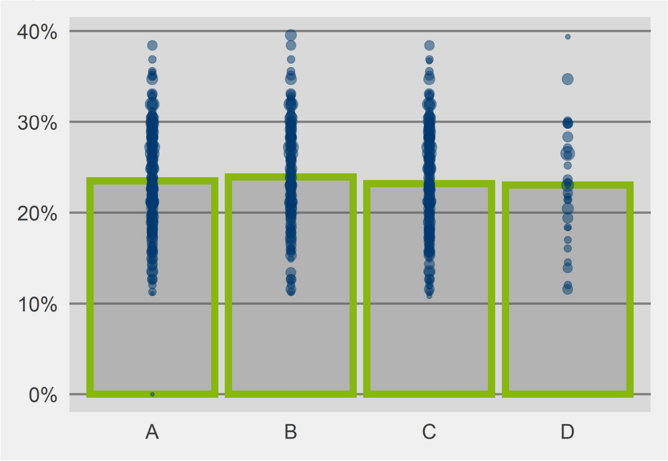

Orthopedic Surgeons (Joint)

Any physical therapy prior to elective hip or knee replacement (%)

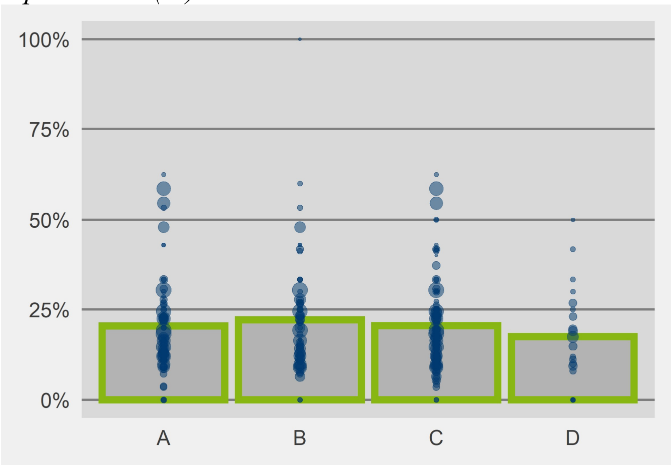

Arthroscopy in patients with new hip or knee osteoarthritis (%)

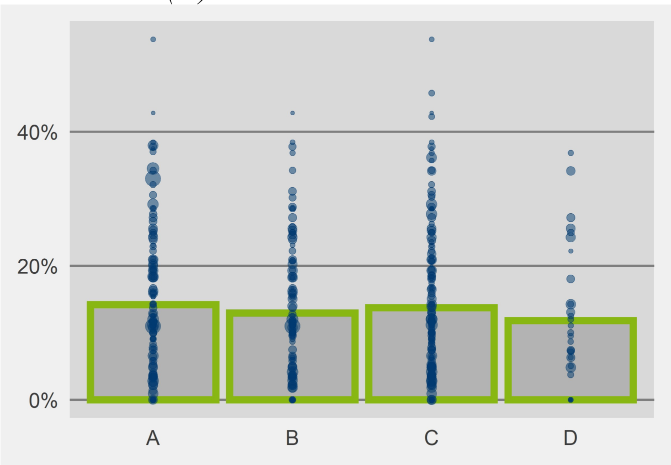

© 2022 Song Z et al. *JAMA Health Forum.*

Orthopedic Surgeons and Neurosurgeons (Spine)

Spinal fusion in patients with low back pain (%)

Any physical therapy in patients with new cervical spine pain (%)

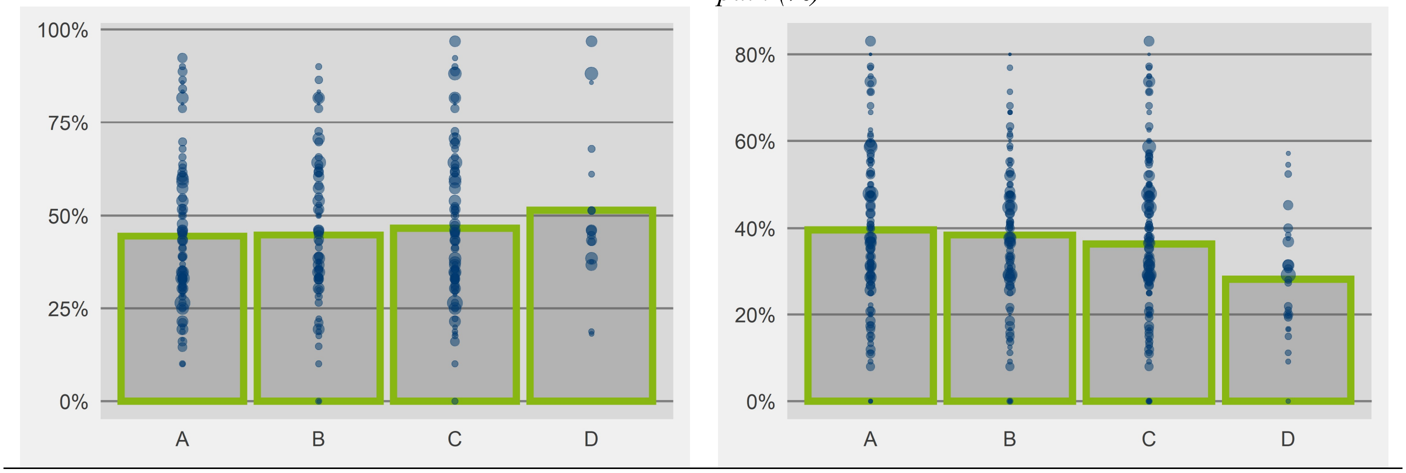

* Data enabling linkages of physicians to their affiliated organization were available only for the South Central metropolitan statistical area (MSA). In this MSA, we were able to identify 4 health systems, each of which is a physician organization (labeled A through D). Linkages were then constructed between providers and provider organizations using affiliation data and website searches. The table preceding the graphs shows the proportion of specialists in each measure that were affiliated with a health system in the MSA. The graphs show the physician-level practice pattern variations captured by the 14 measures within each of the 4 health systems, with each data point corresponding to a physician's unadjusted performance and the size of the data point proportional to the number of attributed patients. The green bar graphs show the average physician-level performance on the measure at the organizational level.

© 2022 Song Z et al. *JAMA Health Forum.*

eTable 1. Physician-Level Variation in Cardiologist Coronary Artery Disease Care*

Stress tests in patients with stable chronic coronary artery disease (per 100 patient years)						
**Region**	**Comparison Group**	**Model 1 Difference vs. Quintile 1 (95% CI)**	**Model 2 Difference vs. Quintile 1 (95% CI)**	**Model 3 Difference vs. Quintile 1 (95% CI)**	**Model 4 Difference vs. Quintile 1 (95% CI)**	**Model 5 Difference vs. Quintile 1 (95% CI)**
Southeast MSA 1	Quintile 2	2.4 (1.8 to 3.1)	2.4 (1.5 to 3.4)	2.5 (1.9 to 3.1)	2.6 (2.0 to 3.3)	2.7 (2.0 to 3.3)
	Quintile 3	4.7 (4.1 to 5.4)	4.7 (3.7 to 5.8)	4.8 (4.1 to 5.4)	4.7 (4.0 to 5.4)	4.7 (4.0 to 5.4)
	Quintile 4	7.5 (6.8 to 8.1)	7.5 (6.4 to 8.6)	7.5 (6.8 to 8.2)	7.6 (6.9 to 8.3)	7.6 (6.9 to 8.3)
	Quintile 5	15.0 (13.2 to 16.9)	15.0 (13.8 to 16.3)	15.0 (13.2 to 16.9)	15.0 (13.1 to 16.9)	15.0 (13.1 to 16.9)
Southeast MSA 2	Quintile 2	3.2 (2.5 to 3.8)	3.2 (2.1 to 4.2)	3.2 (2.5 to 3.8)	3.2 (2.5 to 3.8)	3.2 (2.5 to 3.9)
	Quintile 3	6.2 (5.6 to 6.8)	6.2 (5.1 to 7.3)	6.2 (5.6 to 6.8)	6.3 (5.7 to 7.0)	6.3 (5.7 to 6.9)
	Quintile 4	9.4 (8.7 to 10.1)	9.4 (8.3 to 10.6)	9.4 (8.7 to 10.1)	9.4 (8.7 to 10.1)	9.4 (8.7 to 10.2)
	Quintile 5	17.4 (15.4 to 19.3)	17.4 (16.1 to 18.6)	17.4 (15.4 to 19.4)	17.5 (15.5 to 19.4)	17.4 (15.5 to 19.4)
South Central MSA	Quintile 2	5.0 (4.2 to 5.9)	5.0 (4.1 to 5.9)	5.1 (4.2 to 5.9)	5.0 (4.0 to 6.0)	5.0 (4.0 to 6.0)
	Quintile 3	8.9 (8.1 to 9.7)	8.9 (7.9 to 9.9)	8.9 (8.1 to 9.8)	9.0 (8.0 to 10.0)	9.0 (8.0 to 10.0)
	Quintile 4	13.2 (12.2 to 14.2)	13.2 (12.1 to 14.3)	13.2 (12.2 to 14.2)	13.2 (12.1 to 14.2)	13.2 (12.1 to 14.2)
	Quintile 5	25.4 (23.3 to 27.6)	25.4 (24.2 to 26.7)	25.5 (23.4 to 27.7)	25.8 (23.4 to 28.1)	25.9 (23.5 to 28.2)
Midwest MSA	Quintile 2	3.5 (3.0 to 4.0)	3.5 (2.7 to 4.3)	3.6 (3.0 to 4.1)	3.5 (2.9 to 4.0)	3.6 (3.0 to 4.1)
	Quintile 3	6.7 (6.1 to 7.2)	6.7 (5.8 to 7.5)	6.5 (6.0 to 7.1)	6.6 (6.1 to 7.2)	6.5 (6.0 to 7.0)
	Quintile 4	10.5 (9.9 to 11.1)	10.5 (9.6 to 11.4)	10.4 (9.8 to 11.0)	10.5 (9.9 to 11.1)	10.4 (9.9 to 11.0)
	Quintile 5	21.8 (19.4 to 24.3)	21.8 (20.8 to 22.9)	21.7 (19.3 to 24.1)	21.8 (19.3 to 24.3)	21.7 (19.2 to 24.1)
West MSA	Quintile 2	3.3 (2.5 to 4.0)	3.3 (1.8 to 4.7)	3.3 (2.5 to 4.2)	3.3 (2.5 to 4.1)	3.4 (2.6 to 4.2)
	Quintile 3	6.0 (5.2 to 6.8)	6.0 (4.5 to 7.6)	6.0 (5.2 to 6.9)	6.0 (5.2 to 6.8)	6.0 (5.2 to 6.8)
	Quintile 4	9.9 (9.1 to 10.8)	9.9 (8.2 to 11.6)	10.0 (9.0 to 10.9)	9.9 (9.0 to 10.8)	10.0 (9.1 to 11.0)
	Quintile 5	17.2 (14.8 to 19.6)	17.2 (15.4 to 19.1)	17.2 (14.8 to 19.7)	17.1 (14.7 to 19.4)	17.1 (14.7 to 19.5)

© 2022 Song Z et al. *JAMA Health Forum.*

Statin therapy in patients with chronic coronary artery disease (%)						
**Region**	**Comparison Group**	**Model 1 Difference vs. Quintile 1 (95% CI)**	**Model 2 Difference vs. Quintile 1 (95% CI)**	**Model 3 Difference vs. Quintile 1 (95% CI)**	**Model 4 Difference vs. Quintile 1 (95% CI)**	**Model 5 Difference vs. Quintile 1 (95% CI)**
Southeast MSA 1	Quintile 2	-7.4 (-9.0 to -5.8)	-7.4 (-8.7 to -6.1)	-7.5 (-9.1 to -5.8)	-7.0 (-8.6 to -5.4)	-7.0 (-8.6 to -5.5)
	Quintile 3	-12.0 (-13.5 to -10.4)	-12.0 (-13.2 to -10.7)	-12.0 (-13.6 to -10.5)	-11.7 (-13.2 to -10.2)	-11.7 (-13.2 to -10.2)
	Quintile 4	-17.0 (-18.6 to -15.3)	-17.0 (-18.2 to -15.8)	-17.0 (-18.7 to -15.4)	-16.7 (-18.3 to -15.2)	-16.8 (-18.4 to -15.2)
	Quintile 5	-24.4 (-26.3 to -22.5)	-24.4 (-25.6 to -23.3)	-24.6 (-26.5 to -22.7)	-24.3 (-26.1 to -22.5)	-24.5 (-26.3 to -22.6)
Southeast MSA 2	Quintile 2	-8.8 (-10.4 to -7.3)	-8.8 (-10.2 to -7.5)	-8.8 (-10.4 to -7.3)	-9.0 (-10.6 to -7.5)	-9.0 (-10.6 to -7.5)
	Quintile 3	-13.4 (-14.8 to -11.9)	-13.4 (-14.7 to -12.0)	-13.4 (-14.9 to -11.9)	-13.3 (-14.9 to -11.8)	-13.3 (-14.9 to -11.8)
	Quintile 4	-18.5 (-19.9 to -17.0)	-18.5 (-19.8 to -17.1)	-18.4 (-19.9 to -16.9)	-18.3 (-19.9 to -16.8)	-18.3 (-19.8 to -16.7)
	Quintile 5	-26.9 (-29.1 to -24.7)	-26.9 (-28.1 to -25.7)	-27.0 (-29.2 to -24.7)	-26.9 (-29.0 to -24.8)	-27.0 (-29.1 to -24.9)
South Central MSA	Quintile 2	-9.3 (-10.4 to -8.2)	-9.3 (-10.4 to -8.3)	-9.1 (-10.3 to -7.9)	-8.8 (-10.1 to -7.5)	-8.6 (-9.9 to -7.2)
	Quintile 3	-14.6 (-15.7 to -13.5)	-14.6 (-15.6 to -13.6)	-14.6 (-15.7 to -13.5)	-14.0 (-15.3 to -12.7)	-14.0 (-15.3 to -12.6)
	Quintile 4	-21.1 (-22.2 to -20.0)	-21.1 (-22.2 to -20.0)	-21.0 (-22.2 to -19.9)	-20.6 (-21.9 to -19.4)	-20.6 (-21.9 to -19.3)
	Quintile 5	-32.8 (-35.1 to -30.5)	-32.8 (-33.9 to -31.7)	-32.6 (-34.9 to -30.2)	-32.0 (-34.2 to -29.8)	-31.8 (-34.1 to -29.5)
Midwest MSA	Quintile 2	-6.9 (-7.6 to -6.2)	-6.9 (-7.8 to -6.0)	-7.1 (-7.8 to -6.4)	-7.1 (-7.8 to -6.4)	-7.3 (-8.1 to -6.5)
	Quintile 3	-11.4 (-12.2 to -10.7)	-11.4 (-12.4 to -10.4)	-11.5 (-12.2 to -10.7)	-11.8 (-12.5 to -11.0)	-11.8 (-12.6 to -11.0)
	Quintile 4	-17.0 (-17.8 to -16.2)	-17.0 (-18.0 to -15.9)	-17.0 (-17.8 to -16.2)	-17.6 (-18.4 to -16.8)	-17.7 (-18.5 to -16.9)
	Quintile 5	-27.9 (-29.6 to -26.2)	-27.9 (-29.0 to -26.8)	-28.2 (-29.9 to -26.4)	-28.8 (-30.5 to -27.2)	-29.1 (-30.8 to -27.4)
West MSA	Quintile 2	-7.3 (-9.1 to -5.6)	-7.3 (-9.4 to -5.3)	-7.3 (-9.0 to -5.6)	-7.6 (-9.2 to -6.0)	-7.6 (-9.2 to -6.0)
	Quintile 3	-12.4 (-14.0 to -10.8)	-12.4 (-14.6 to -10.2)	-12.3 (-13.9 to -10.7)	-12.1 (-13.7 to -10.5)	-12.1 (-13.7 to -10.4)
	Quintile 4	-17.6 (-19.4 to -15.8)	-17.6 (-19.7 to -15.5)	-17.5 (-19.4 to -15.7)	-17.3 (-19.0 to -15.5)	-17.2 (-19.0 to -15.3)
	Quintile 5	-30.5 (-33.5 to -27.6)	-30.5 (-32.8 to -28.3)	-30.3 (-33.2 to -27.4)	-30.2 (-33.2 to -27.3)	-30.1 (-32.9 to -27.2)

* Differences between quintile 1 (reference) and quintiles 2-5 are derived from a model adjusted for risk score (age, sex, and diagnoses) and socioeconomic variables, with standard errors clustered by physician. 95% confidence intervals are shown in parentheses. Model 1 results are the main estimates. Sensitivity analyses tested the robustness of the main estimates to alterations in the model, focusing on the role of

© 2022 Song Z et al. *JAMA Health Forum.*

clinical risk and socioeconomic status adjustment. Model 2 omitted clustered standard errors. Model 3 omitted the DxCG risk score. Model 4 omitted the SES score. Model 5 omitted both the DxCG and SES scores. Stable estimates among these sensitivity analyses relative to Model 1 would suggest that any observable differences in patient age, sex, clinical diagnoses, and socioeconomic status characteristics across physician quintiles contributed minimal bias toward the differences in performance between the quintiles. However, they would not adjust for all potential confounders.

© 2022 Song Z et al. *JAMA Health Forum.*

eTable 2. Physician-Level Variation in Endocrinologist Diabetes Care*

Kidney function test in patients with diabetes (%)						
**Region**	**Comparison Group**	**Model 1 Difference vs. Quintile 1 (95% CI)**	**Model 2 Difference vs. Quintile 1 (95% CI)**	**Model 3 Difference vs. Quintile 1 (95% CI)**	**Model 4 Difference vs. Quintile 1 (95% CI)**	**Model 5 Difference vs. Quintile 1 (95% CI)**
Southeast MSA 1	Quintile 2	-3.0	-3.0	-2.4	-3.3	-2.9
		(-4.4 to -1.5)	(-4.7 to -1.2)	(-3.8 to -1.0)	(-4.9 to -1.8)	(-4.5 to -1.3)
	Quintile 3	-8.2	-8.2	-7.9	-8.1	-7.9
		(-9.8 to -6.5)	(-10.0 to -6.4)	(-9.5 to -6.3)	(-9.8 to -6.5)	(-9.5 to -6.2)
	Quintile 4	-13.7	-13.7	-13.3	-13.6	-13.3
		(-16.0 to -11.5)	(-15.5 to -12.0)	(-15.6 to -11.1)	(-15.9 to -11.3)	(-15.6 to -11.0)
	Quintile 5	-27.7	-27.7	-27.1	-27.6	-27.0
		(-32.6 to -22.9)	(-29.7 to -25.7)	(-32.0 to -22.2)	(-32.2 to -23.0)	(-31.7 to -22.4)
Southeast MSA 2	Quintile 2	-7.9	-7.9	-7.7	-7.9	-7.7
		(-10.5 to -5.3)	(-9.3 to -6.4)	(-10.1 to -5.2)	(-10.4 to -5.4)	(-10.1 to -5.3)
	Quintile 3	-16.0	-16.0	-15.5	-16.0	-15.5
		(-18.0 to -14.1)	(-17.7 to -14.3)	(-17.3 to -13.6)	(-18.0 to -14.0)	(-17.4 to -13.6)
	Quintile 4	-23.7	-23.7	-23.4	-23.6	-23.3
		(-26.0 to -21.4)	(-25.3 to -22.1)	(-25.6 to -21.1)	(-25.8 to -21.4)	(-25.5 to -21.1)
	Quintile 5	-37.0	-37.0	-36.7	-37.1	-36.8
		(-43.9 to -30.1)	(-39.0 to -35.0)	(-43.4 to -29.9)	(-44.1 to -30.1)	(-43.6 to -29.9)
South Central MSA	Quintile 2	-3.3	-3.3	-3.1	-3.3	-3.1
		(-4.2 to -2.3)	(-3.9 to -2.6)	(-4.1 to -2.0)	(-4.3 to -2.4)	(-4.0 to -2.1)
	Quintile 3	-6.8	-6.8	-6.5	-6.8	-6.4
		(-7.9 to -5.7)	(-7.6 to -6.1)	(-7.6 to -5.4)	(-7.8 to -5.7)	(-7.5 to -5.4)
	Quintile 4	-11.2	-11.2	-10.9	-11.0	-10.7
		(-12.8 to -9.5)	(-12.0 to -10.4)	(-12.7 to -9.2)	(-12.6 to -9.4)	(-12.4 to -9.1)
	Quintile 5	-24.1	-24.1	-23.9	-23.7	-23.4
		(-27.1 to -21.0)	(-24.9 to -23.2)	(-27.0 to -20.7)	(-26.7 to -20.7)	(-26.5 to -20.3)
Midwest MSA	Quintile 2	-5.5	-5.5	-5.6	-5.6	-5.6
		(-6.6 to -4.4)	(-6.3 to -4.7)	(-6.7 to -4.5)	(-6.7 to -4.5)	(-6.7 to -4.5)
	Quintile 3	-11.9	-11.9	-11.9	-11.9	-11.8
		(-13.1 to -10.7)	(-12.7 to -11.0)	(-13.0 to -10.7)	(-13.0 to -10.7)	(-13.0 to -10.7)
	Quintile 4	-18.6	-18.6	-18.5	-18.6	-18.5
		(-20.3 to -16.8)	(-19.5 to -17.7)	(-20.3 to -16.6)	(-20.4 to -16.8)	(-20.4 to -16.6)
	Quintile 5	-35.9	-35.9	-36.1	-36.0	-36.2
		(-39.4 to -32.4)	(-36.9 to -34.9)	(-39.5 to -32.6)	(-39.5 to -32.5)	(-39.6 to -32.7)
West MSA	Quintile 2	-8.4	-8.4	-8.4	-8.3	-8.3
		(-11.4 to -5.5)	(-9.9 to -7.0)	(-11.3 to -5.5)	(-11.1 to -5.6)	(-11.0 to -5.6)
	Quintile 3	-22.0	-22.0	-21.9	-21.9	-21.8
		(-25.8 to -18.2)	(-23.8 to -20.2)	(-25.6 to -18.1)	(-25.7 to -18.2)	(-25.5 to -18.2)
	Quintile 4	-48.3	-48.3	-48.0	-47.8	-47.5
		(-51.8 to -44.9)	(-50.0 to -46.7)	(-51.4 to -44.6)	(-51.2 to -44.4)	(-50.9 to -44.1)
	Quintile 5	-62.1	-62.1	-62.1	-62.3	-62.3
		(-66.5 to -57.7)	(-63.6 to -60.7)	(-66.4 to -57.7)	(-66.6 to -58.0)	(-66.6 to -58.0)

© 2022 Song Z et al. *JAMA Health Forum.*

Oral glucose-lowering medication in patients with diabetes (%)						
**Region**	**Comparison Group**	**Model 1 Difference vs. Quintile 1 (95% CI)**	**Model 2 Difference vs. Quintile 1 (95% CI)**	**Model 3 Difference vs. Quintile 1 (95% CI)**	**Model 4 Difference vs. Quintile 1 (95% CI)**	**Model 5 Difference vs. Quintile 1 (95% CI)**
Southeast MSA 1	Quintile 2	-5.3	-5.3	-5.2	-5.3	-5.3
		(-7.3 to -3.3)	(-7.3 to -3.2)	(-7.3 to -3.2)	(-7.1 to -3.5)	(-7.1 to -3.4)
	Quintile 3	-8.5	-8.5	-8.5	-8.5	-8.5
		(-10.5 to -6.5)	(-10.7 to -6.3)	(-10.5 to -6.6)	(-10.4 to -6.5)	(-10.4 to -6.6)
	Quintile 4	-11.4	-11.4	-11.4	-10.8	-10.8
		(-13.5 to -9.2)	(-13.3 to -9.4)	(-13.5 to -9.2)	(-12.7 to -8.8)	(-12.7 to -8.9)
	Quintile 5	-23.2	-23.2	-23.2	-23.1	-23.1
		(-27.6 to -18.9)	(-25.4 to -21.1)	(-27.6 to -18.9)	(-27.3 to -18.8)	(-27.3 to -18.8)
Southeast MSA 2	Quintile 2	-4.7	-4.7	-4.7	-5.2	-5.2
		(-6.6 to -2.7)	(-6.6 to -2.7)	(-6.7 to -2.8)	(-7.0 to -3.3)	(-7.1 to -3.3)
	Quintile 3	-8.8	-8.8	-8.8	-8.3	-8.3
		(-10.4 to -7.1)	(-10.7 to -6.8)	(-10.5 to -7.1)	(-10.1 to -6.5)	(-10.1 to -6.5)
	Quintile 4	-13.3	-13.3	-13.3	-13.3	-13.3
		(-15.3 to -11.2)	(-15.3 to -11.2)	(-15.3 to -11.3)	(-15.2 to -11.4)	(-15.2 to -11.5)
	Quintile 5	-20.8	-20.8	-20.8	-21.4	-21.4
		(-28.2 to -13.3)	(-23.1 to -18.5)	(-28.3 to -13.3)	(-28.5 to -14.4)	(-28.5 to -14.4)
South Central MSA	Quintile 2	-5.3	-5.3	-5.5	-5.1	-5.2
		(-7.1 to -3.5)	(-6.6 to -4.1)	(-7.3 to -3.7)	(-6.9 to -3.2)	(-7.0 to -3.4)
	Quintile 3	-10.3	-10.3	-10.4	-10.3	-10.4
		(-12.1 to -8.5)	(-11.6 to -9.0)	(-12.3 to -8.6)	(-12.1 to -8.5)	(-12.3 to -8.6)
	Quintile 4	-15.4	-15.4	-15.5	-14.4	-14.5
		(-17.4 to -13.3)	(-16.7 to -14.0)	(-17.5 to -13.5)	(-16.4 to -12.5)	(-16.4 to -12.6)
	Quintile 5	-26.8	-26.8	-27.1	-25.3	-25.5
		(-29.9 to -23.7)	(-28.5 to -25.1)	(-30.2 to -24.0)	(-28.6 to -22.0)	(-28.9 to -22.2)
Midwest MSA	Quintile 2	-5.9	-5.9	-6.1	-5.9	-6.1
		(-7.2 to -4.6)	(-7.1 to -4.7)	(-7.4 to -4.7)	(-7.3 to -4.5)	(-7.5 to -4.7)
	Quintile 3	-9.7	-9.7	-9.8	-9.8	-9.9
		(-11.1 to -8.2)	(-11.0 to -8.3)	(-11.3 to -8.4)	(-11.2 to -8.3)	(-11.3 to -8.5)
	Quintile 4	-14.3	-14.3	-14.2	-14.5	-14.4
		(-15.7 to -12.9)	(-15.6 to -13.0)	(-15.7 to -12.8)	(-15.9 to -13.2)	(-15.8 to -13.0)
	Quintile 5	-24.6	-24.6	-24.7	-24.9	-25.0
		(-28.6 to -20.5)	(-25.8 to -23.3)	(-28.6 to -20.7)	(-29.1 to -20.7)	(-29.1 to -20.8)
West MSA	Quintile 2	-5.3	-5.3	-5.0	-5.4	-5.2
		(-7.8 to -2.7)	(-8.4 to -2.1)	(-7.5 to -2.4)	(-7.5 to -3.2)	(-7.3 to -3.1)
	Quintile 3	-13.6	-13.6	-12.8	-14.0	-13.0
		(-15.9 to -11.4)	(-16.5 to -10.8)	(-15.0 to -10.5)	(-16.3 to -11.6)	(-15.4 to -10.7)
	Quintile 4	-17.9	-17.9	-17.5	-18.4	-18.1
		(-20.1 to -15.7)	(-20.6 to -15.2)	(-19.7 to -15.3)	(-20.7 to -16.2)	(-20.3 to -15.8)
	Quintile 5	-31.2	-31.2	-30.9	-31.9	-31.7
		(-36.8 to -25.5)	(-33.9 to -28.4)	(-36.6 to -25.2)	(-36.9 to -26.9)	(-36.7 to -26.8)

* Differences between quintile 1 (reference) and quintiles 2-5 are derived from a model adjusted for risk score (age, sex, and diagnoses) and socioeconomic variables, with standard errors clustered by physician. 95% confidence intervals are shown in parentheses. Model 1 results are the main estimates. Sensitivity analyses tested the robustness of the main estimates to alterations in the model, focusing on the role of

© 2022 Song Z et al. *JAMA Health Forum.*

clinical risk and socioeconomic status adjustment. Model 2 omitted clustered standard errors. Model 3 omitted the DxCG risk score. Model 4 omitted the SES score. Model 5 omitted both the DxCG and SES scores. Stable estimates among these sensitivity analyses relative to Model 1 would suggest that any observable differences in patient age, sex, clinical diagnoses, and socioeconomic status characteristics across physician quintiles contributed minimal bias toward the differences in performance between the quintiles. However, they would not adjust for all potential confounders.

© 2022 Song Z et al. *JAMA Health Forum.*

eTable 3. Physician-Level Variation in Gastroenterologist Gastrointestinal Tract Care*

Polyp detected on screening colonoscopy (%)						
**Region**	**Comparison Group**	**Model 1 Difference vs. Quintile 1 (95% CI)**	**Model 2 Difference vs. Quintile 1 (95% CI)**	**Model 3 Difference vs. Quintile 1 (95% CI)**	**Model 4 Difference vs. Quintile 1 (95% CI)**	**Model 5 Difference vs. Quintile 1 (95% CI)**
Southeast MSA 1	Quintile 2	-12.7 (-15.7 to -9.7)	-12.7 (-14.5 to -10.9)	-12.7 (-15.7 to -9.7)	-12.8 (-15.8 to -9.7)	-12.7 (-15.7 to -9.7)
	Quintile 3	-20.6 (-23.7 to -17.5)	-20.6 (-22.4 to -18.8)	-20.6 (-23.7 to -17.5)	-20.4 (-23.5 to -17.3)	-20.4 (-23.5 to -17.4)
	Quintile 4	-30.8 (-33.7 to -27.9)	-30.8 (-32.5 to -29.1)	-30.8 (-33.7 to -27.9)	-30.8 (-33.6 to -27.9)	-30.8 (-33.7 to -28.0)
	Quintile 5	-42.6 (-46.4 to -38.7)	-42.6 (-44.3 to -40.9)	-42.6 (-46.4 to -38.7)	-42.4 (-46.3 to -38.5)	-42.4 (-46.2 to -38.5)
Southeast MSA 2	Quintile 2	-14.3 (-16.6 to -12.1)	-14.3 (-15.9 to -12.8)	-14.5 (-16.8 to -12.2)	-14.3 (-16.6 to -12.1)	-14.5 (-16.7 to -12.2)
	Quintile 3	-21.8 (-24.1 to -19.6)	-21.8 (-23.4 to -20.3)	-21.8 (-24.1 to -19.6)	-21.6 (-23.9 to -19.3)	-21.6 (-23.9 to -19.3)
	Quintile 4	-32.7 (-35.3 to -30.1)	-32.7 (-34.2 to -31.1)	-32.7 (-35.3 to -30.2)	-32.4 (-35.1 to -29.7)	-32.4 (-35.1 to -29.8)
	Quintile 5	-46.7 (-50.9 to -42.4)	-46.7 (-48.1 to -45.2)	-46.7 (-50.9 to -42.5)	-46.2 (-50.4 to -41.9)	-46.3 (-50.5 to -42.0)
South Central MSA	Quintile 2	-11.5 (-13.3 to -9.7)	-11.5 (-12.3 to -10.7)	-11.4 (-13.3 to -9.6)	-11.6 (-13.4 to -9.8)	-11.5 (-13.4 to -9.7)
	Quintile 3	-19.7 (-21.4 to -17.9)	-19.7 (-20.5 to -18.8)	-19.7 (-21.5 to -18.0)	-19.5 (-21.2 to -17.9)	-19.6 (-21.3 to -17.9)
	Quintile 4	-27.6 (-29.4 to -25.7)	-27.6 (-28.4 to -26.7)	-27.5 (-29.4 to -25.6)	-27.6 (-29.4 to -25.7)	-27.5 (-29.4 to -25.6)
	Quintile 5	-39.4 (-41.7 to -37.2)	-39.4 (-40.3 to -38.6)	-39.5 (-41.7 to -37.3)	-39.2 (-41.4 to -37.0)	-39.2 (-41.4 to -37.1)
Midwest MSA	Quintile 2	-15.7 (-18.4 to -12.9)	-15.7 (-16.4 to -14.9)	-15.5 (-18.2 to -12.8)	-15.6 (-18.3 to -12.8)	-15.4 (-18.2 to -12.7)
	Quintile 3	-23.0 (-25.7 to -20.3)	-23.0 (-23.8 to -22.2)	-23.0 (-25.7 to -20.3)	-23.0 (-25.7 to -20.4)	-23.0 (-25.7 to -20.4)
	Quintile 4	-30.1 (-32.8 to -27.5)	-30.1 (-30.9 to -29.4)	-30.0 (-32.7 to -27.3)	-30.1 (-32.8 to -27.4)	-30.0 (-32.7 to -27.3)
	Quintile 5	-43.4 (-46.6 to -40.3)	-43.4 (-44.2 to -42.6)	-43.4 (-46.6 to -40.3)	-43.4 (-46.6 to -40.3)	-43.4 (-46.5 to -40.3)
West MSA	Quintile 2	-8.2 (-10.0 to -6.4)	-8.2 (-9.6 to -6.9)	-8.1 (-9.9 to -6.2)	-8.0 (-9.8 to -6.2)	-7.9 (-9.7 to -6.0)
	Quintile 3	-13.7 (-15.5 to -11.9)	-13.7 (-15.1 to -12.3)	-13.4 (-15.3 to -11.6)	-13.5 (-15.3 to -11.6)	-13.2 (-15.1 to -11.3)
	Quintile 4	-20.9 (-22.6 to -19.1)	-20.9 (-22.3 to -19.4)	-20.7 (-22.4 to -18.9)	-20.5 (-22.2 to -18.9)	-20.4 (-22.0 to -18.7)
	Quintile 5	-31.9 (-35.0 to -28.9)	-31.9 (-33.4 to -30.4)	-31.9 (-35.0 to -28.8)	-31.6 (-34.6 to -28.5)	-31.5 (-34.6 to -28.4)

© 2022 Song Z et al. *JAMA Health Forum.*

Endoscopy in patients with GERD and no alarm symptoms (%)						
**Region**	**Comparison Group**	**Model 1 Difference vs. Quintile 1 (95% CI)**	**Model 2 Difference vs. Quintile 1 (95% CI)**	**Model 3 Difference vs. Quintile 1 (95% CI)**	**Model 4 Difference vs. Quintile 1 (95% CI)**	**Model 5 Difference vs. Quintile 1 (95% CI)**
Southeast MSA 1	Quintile 2	4.6 (3.5 to 5.8)	4.6 (3.3 to 6.0)	4.6 (3.5 to 5.7)	4.6 (3.5 to 5.7)	4.6 (3.5 to 5.7)
	Quintile 3	7.8 (6.7 to 8.8)	7.8 (6.3 to 9.2)	7.7 (6.6 to 8.8)	7.7 (6.7 to 8.8)	7.7 (6.6 to 8.8)
	Quintile 4	11.0 (9.9 to 12.2)	11.0 (9.5 to 12.5)	11.1 (9.9 to 12.2)	11.0 (9.8 to 12.2)	11.1 (9.9 to 12.3)
	Quintile 5	15.4 (13.7 to 17.1)	15.4 (14.0 to 16.9)	15.4 (13.7 to 17.1)	15.3 (13.7 to 17.0)	15.3 (13.6 to 17.0)
Southeast MSA 2	Quintile 2	3.5 (2.4 to 4.6)	3.5 (2.2 to 4.8)	3.5 (2.4 to 4.6)	3.5 (2.4 to 4.5)	3.5 (2.4 to 4.5)
	Quintile 3	5.9 (4.9 to 7.0)	5.9 (4.6 to 7.3)	5.9 (4.9 to 6.9)	5.9 (4.8 to 6.9)	5.8 (4.8 to 6.9)
	Quintile 4	8.5 (7.5 to 9.6)	8.5 (7.2 to 9.8)	8.5 (7.4 to 9.6)	8.4 (7.4 to 9.5)	8.4 (7.3 to 9.5)
	Quintile 5	12.3 (10.9 to 13.8)	12.3 (11.1 to 13.6)	12.3 (10.9 to 13.8)	12.3 (10.8 to 13.7)	12.3 (10.8 to 13.7)
South Central MSA	Quintile 2	4.4 (3.8 to 5.0)	4.4 (3.7 to 5.1)	4.4 (3.8 to 5.0)	4.5 (3.8 to 5.2)	4.5 (3.8 to 5.2)
	Quintile 3	7.1 (6.5 to 7.8)	7.1 (6.5 to 7.8)	7.1 (6.4 to 7.7)	7.1 (6.4 to 7.8)	7.0 (6.3 to 7.7)
	Quintile 4	11.0 (10.3 to 11.7)	11.0 (10.3 to 11.7)	10.9 (10.2 to 11.6)	10.8 (10.0 to 11.5)	10.7 (9.9 to 11.4)
	Quintile 5	15.8 (14.8 to 16.8)	15.8 (15.1 to 16.5)	15.7 (14.7 to 16.8)	15.7 (14.7 to 16.7)	15.6 (14.5 to 16.6)
Midwest MSA	Quintile 2	5.5 (4.6 to 6.4)	5.5 (4.8 to 6.2)	5.5 (4.6 to 6.4)	5.5 (4.6 to 6.4)	5.5 (4.6 to 6.4)
	Quintile 3	8.7 (7.8 to 9.6)	8.7 (8.0 to 9.4)	8.7 (7.8 to 9.6)	8.7 (7.8 to 9.6)	8.7 (7.8 to 9.6)
	Quintile 4	12.2 (11.3 to 13.1)	12.2 (11.5 to 12.9)	12.2 (11.3 to 13.1)	12.2 (11.3 to 13.1)	12.1 (11.2 to 13.0)
	Quintile 5	19.4 (17.6 to 21.3)	19.4 (18.7 to 20.1)	19.2 (17.4 to 21.1)	19.4 (17.5 to 21.2)	19.2 (17.3 to 21.1)
West MSA	Quintile 2	3.6 (2.7 to 4.5)	3.6 (2.4 to 4.8)	3.6 (2.8 to 4.5)	3.7 (2.8 to 4.6)	3.7 (2.8 to 4.6)
	Quintile 3	6.4 (5.5 to 7.3)	6.4 (5.1 to 7.7)	6.5 (5.6 to 7.4)	6.3 (5.4 to 7.2)	6.4 (5.5 to 7.3)
	Quintile 4	8.9 (8.1 to 9.8)	8.9 (7.8 to 10.1)	9.0 (8.2 to 9.8)	8.7 (7.8 to 9.6)	8.8 (7.9 to 9.7)
	Quintile 5	12.8 (11.6 to 13.9)	12.8 (11.5 to 14.0)	12.8 (11.6 to 14.0)	12.6 (11.4 to 13.8)	12.7 (11.5 to 13.9)

* Differences between quintile 1 (reference) and quintiles 2-5 are derived from a model adjusted for risk score (age, sex, and diagnoses) and socioeconomic variables, with standard errors clustered by physician. 95% confidence intervals are shown in parentheses. Model 1 results are the main estimates. Sensitivity analyses tested the robustness of the main estimates to alterations in the model, focusing on the role of

© 2022 Song Z et al. *JAMA Health Forum.*

clinical risk and socioeconomic status adjustment. Model 2 omitted clustered standard errors. Model 3 omitted the DxCG risk score. Model 4 omitted the SES score. Model 5 omitted both the DxCG and SES scores. Stable estimates among these sensitivity analyses relative to Model 1 would suggest that any observable differences in patient age, sex, clinical diagnoses, and socioeconomic status characteristics across physician quintiles contributed minimal bias toward the differences in performance between the quintiles. However, they would not adjust for all potential confounders.

© 2022 Song Z et al. *JAMA Health Forum.*

eTable 4. Physician-Level Variation in Pulmonologist COPD Care*

Bronchodilator in patients with chronic obstructive pulmonary disease (COPD) (%)						
**Region**	**Comparison Group**	**Model 1 Difference vs. Quintile 1 (95% CI)**	**Model 2 Difference vs. Quintile 1 (95% CI)**	**Model 3 Difference vs. Quintile 1 (95% CI)**	**Model 4 Difference vs. Quintile 1 (95% CI)**	**Model 5 Difference vs. Quintile 1 (95% CI)**
Southeast MSA 1	Quintile 2	-8.0	-8.0	-8.0	-7.2	-7.2
		(-10.6 to -5.3)	(-11.1 to -4.8)	(-10.8 to -5.2)	(-9.5 to -4.8)	(-9.6 to -4.7)
	Quintile 3	-12.2	-12.2	-12.1	-12.0	-11.9
		(-14.4 to -10.0)	(-15.3 to -9.2)	(-14.5 to -9.8)	(-14.4 to -9.7)	(-14.4 to -9.4)
	Quintile 4	-15.7	-15.7	-15.8	-14.8	-14.8
		(-18.0 to -13.4)	(-18.9 to -12.5)	(-18.3 to -13.3)	(-17.3 to -12.4)	(-17.4 to -12.3)
	Quintile 5	-22.9	-22.9	-23.1	-21.9	-22.1
		(-26.2 to -19.6)	(-26.1 to -19.7)	(-26.4 to -19.8)	(-24.9 to -18.9)	(-25.1 to -19.1)
Southeast MSA 2	Quintile 2	-8.9	-8.9	-8.8	-8.6	-8.5
		(-11.1 to -6.8)	(-12.0 to -5.9)	(-10.9 to -6.6)	(-10.7 to -6.6)	(-10.5 to -6.4)
	Quintile 3	-14.9	-14.9	-14.8	-14.8	-14.7
		(-17.1 to -12.7)	(-17.9 to -11.9)	(-17.0 to -12.6)	(-16.9 to -12.8)	(-16.9 to -12.6)
	Quintile 4	-19.4	-19.4	-19.4	-19.1	-19.0
		(-21.8 to -17.1)	(-22.3 to -16.6)	(-21.7 to -17.1)	(-21.3 to -16.9)	(-21.2 to -16.8)
	Quintile 5	-25.3	-25.3	-25.2	-25.3	-25.2
		(-32.0 to -18.6)	(-28.3 to -22.3)	(-31.8 to -18.6)	(-31.7 to -18.9)	(-31.6 to -18.8)
South Central MSA	Quintile 2	-11.6	-11.6	-11.8	-11.3	-11.5
		(-14.1 to -9.0)	(-14.5 to -8.6)	(-14.3 to -9.3)	(-14.0 to -8.7)	(-14.1 to -8.9)
	Quintile 3	-19.6	-19.6	-19.6	-18.6	-18.7
		(-22.3 to -16.9)	(-22.7 to -16.6)	(-22.2 to -17.0)	(-21.1 to -16.1)	(-21.1 to -16.3)
	Quintile 4	-25.2	-25.2	-25.2	-24.8	-24.9
		(-27.8 to -22.5)	(-28.1 to -22.3)	(-27.8 to -22.7)	(-27.3 to -22.3)	(-27.4 to -22.5)
	Quintile 5	-38.8	-38.8	-38.8	-38.5	-38.5
		(-41.6 to -36.1)	(-42.0 to -35.6)	(-41.5 to -36.2)	(-41.4 to -35.7)	(-41.2 to -35.8)
Midwest MSA	Quintile 2	-10.6	-10.6	-10.5	-10.3	-10.2
		(-13.1 to -8.1)	(-13.5 to -7.7)	(-13.0 to -8.0)	(-12.9 to -7.8)	(-12.7 to -7.7)
	Quintile 3	-17.2	-17.2	-17.5	-17.2	-17.5
		(-19.6 to -14.8)	(-20.1 to -14.3)	(-19.9 to -15.1)	(-19.7 to -14.7)	(-20.0 to -15.0)
	Quintile 4	-24.1	-24.1	-24.2	-24.0	-24.1
		(-26.6 to -21.5)	(-26.8 to -21.3)	(-26.7 to -21.7)	(-26.6 to -21.4)	(-26.6 to -21.6)
	Quintile 5	-33.7	-33.7	-33.6	-33.4	-33.2
		(-36.7 to -30.7)	(-36.6 to -30.8)	(-36.6 to -30.5)	(-36.5 to -30.3)	(-36.4 to -30.1)
West MSA	Quintile 2	-10.8	-10.8	-11.0	-11.8	-12.2
		(-13.7 to -7.8)	(-16.3 to -5.3)	(-14.0 to -8.1)	(-16.0 to -7.5)	(-16.8 to -7.6)
	Quintile 3	-16.6	-16.6	-16.7	-16.4	-16.4
		(-19.5 to -13.7)	(-22.4 to -10.8)	(-19.8 to -13.7)	(-19.5 to -13.3)	(-19.7 to -13.1)
	Quintile 4	-25.0	-25.0	-24.8	-25.0	-24.7
		(-28.4 to -21.5)	(-31.0 to -19.0)	(-28.5 to -21.0)	(-29.5 to -20.4)	(-29.9 to -19.6)
	Quintile 5	-37.1	-37.1	-36.3	-37.5	-36.8
		(-41.1 to -33.1)	(-43.0 to -31.2)	(-40.2 to -32.5)	(-42.0 to -33.0)	(-41.6 to -32.1)

© 2022 Song Z et al. *JAMA Health Forum.*

Spirometry in patients with chronic obstructive pulmonary disease (COPD)						
**Region**	**Comparison Group**	**Model 1 Difference vs. Quintile 1 (95% CI)**	**Model 2 Difference vs. Quintile 1 (95% CI)**	**Model 3 Difference vs. Quintile 1 (95% CI)**	**Model 4 Difference vs. Quintile 1 (95% CI)**	**Model 5 Difference vs. Quintile 1 (95% CI)**
Southeast MSA 1	Quintile 2	-9.3	-9.3	-9.3	-9.3	-9.2
		(-11.5 to -7.2)	(-11.5 to -7.2)	(-11.4 to -7.1)	(-11.4 to -7.2)	(-11.4 to -7.1)
	Quintile 3	-18.0	-18.0	-18.0	-17.5	-17.5
		(-20.1 to -15.9)	(-20.4 to -15.6)	(-20.1 to -15.9)	(-19.6 to -15.4)	(-19.6 to -15.3)
	Quintile 4	-25.7	-25.7	-25.8	-24.9	-24.9
		(-28.4 to -23.0)	(-28.2 to -23.2)	(-28.4 to -23.1)	(-28.0 to -21.7)	(-28.1 to -21.8)
	Quintile 5	-41.5	-41.5	-41.6	-40.6	-40.7
		(-45.0 to -37.9)	(-44.1 to -38.8)	(-45.1 to -38.0)	(-44.1 to -37.1)	(-44.2 to -37.2)
Southeast MSA 2	Quintile 2	-11.9	-11.9	-11.9	-11.5	-11.4
		(-15.0 to -8.8)	(-14.2 to -9.6)	(-15.0 to -8.8)	(-14.6 to -8.3)	(-14.6 to -8.3)
	Quintile 3	-21.1	-21.1	-21.0	-20.6	-20.5
		(-23.7 to -18.4)	(-23.6 to -18.6)	(-23.8 to -18.3)	(-23.3 to -17.9)	(-23.3 to -17.7)
	Quintile 4	-29.4	-29.4	-29.4	-29.2	-29.1
		(-32.1 to -26.8)	(-32.1 to -26.8)	(-32.1 to -26.6)	(-32.0 to -26.3)	(-32.0 to -26.1)
	Quintile 5	-46.7	-46.7	-46.7	-46.3	-46.3
		(-54.8 to -38.7)	(-49.5 to -44.0)	(-54.9 to -38.6)	(-54.6 to -38.0)	(-54.7 to -37.9)
South Central MSA	Quintile 2	-6.9	-6.9	-6.9	-6.7	-6.8
		(-8.1 to -5.7)	(-7.9 to -5.9)	(-8.1 to -5.7)	(-8.0 to -5.5)	(-8.0 to -5.5)
	Quintile 3	-15.4	-15.4	-15.6	-15.5	-15.6
		(-16.4 to -14.5)	(-16.7 to -14.2)	(-16.5 to -14.6)	(-16.5 to -14.4)	(-16.7 to -14.5)
	Quintile 4	-25.9	-25.9	-26.0	-25.4	-25.4
		(-28.4 to -23.5)	(-27.4 to -24.5)	(-28.5 to -23.5)	(-28.1 to -22.8)	(-28.1 to -22.8)
	Quintile 5	-40.1	-40.1	-40.1	-39.8	-39.8
		(-42.7 to -37.5)	(-41.7 to -38.5)	(-42.7 to -37.5)	(-42.3 to -37.2)	(-42.3 to -37.3)
Midwest MSA	Quintile 2	-12.4	-12.4	-12.1	-12.4	-12.0
		(-14.4 to -10.4)	(-13.8 to -10.9)	(-14.1 to -10.1)	(-14.4 to -10.3)	(-14.0 to -10.0)
	Quintile 3	-24.1	-24.1	-24.1	-24.0	-23.9
		(-25.7 to -22.4)	(-25.6 to -22.5)	(-25.7 to -22.4)	(-25.7 to -22.3)	(-25.6 to -22.3)
	Quintile 4	-32.5	-32.5	-32.2	-32.3	-32.0
		(-34.1 to -30.8)	(-34.1 to -30.8)	(-33.8 to -30.5)	(-33.9 to -30.7)	(-33.7 to -30.3)
	Quintile 5	-50.3	-50.3	-50.1	-50.4	-50.2
		(-53.8 to -46.7)	(-51.9 to -48.6)	(-53.8 to -46.5)	(-53.9 to -46.8)	(-53.8 to -46.6)
West MSA	Quintile 2	-11.6	-11.6	-11.6	-11.6	-11.5
		(-14.6 to -8.7)	(-14.4 to -8.8)	(-14.7 to -8.4)	(-14.3 to -8.9)	(-14.4 to -8.6)
	Quintile 3	-24.2	-24.2	-24.1	-24.0	-23.9
		(-28.0 to -20.3)	(-27.3 to -21.1)	(-28.1 to -20.2)	(-27.8 to -20.2)	(-27.9 to -20.0)
	Quintile 4	-38.2	-38.2	-38.0	-37.8	-37.6
		(-41.7 to -34.6)	(-41.5 to -34.8)	(-41.5 to -34.5)	(-41.7 to -34.0)	(-41.4 to -33.9)
	Quintile 5	-58.9	-58.9	-59.2	-58.4	-58.7
		(-67.0 to -50.9)	(-62.1 to -55.8)	(-67.1 to -51.3)	(-66.9 to -49.9)	(-67.2 to -50.3)

* Differences between quintile 1 (reference) and quintiles 2-5 are derived from a model adjusted for risk score (age, sex, and diagnoses) and socioeconomic variables, with standard errors clustered by physician. 95% confidence intervals are shown in parentheses. Model 1 results are the main estimates. Sensitivity

© 2022 Song Z et al. *JAMA Health Forum.*

analyses tested the robustness of the main estimates to alterations in the model, focusing on the role of clinical risk and socioeconomic status adjustment. Model 2 omitted clustered standard errors. Model 3 omitted the DxCG risk score. Model 4 omitted the SES score. Model 5 omitted both the DxCG and SES scores. Stable estimates among these sensitivity analyses relative to Model 1 would suggest that any observable differences in patient age, sex, clinical diagnoses, and socioeconomic status characteristics across physician quintiles contributed minimal bias toward the differences in performance between the quintiles. However, they would not adjust for all potential confounders.

© 2022 Song Z et al. *JAMA Health Forum.*

eTable 5. Physician-Level Variation in Obstetrician Prenatal and Delivery Care*

Appropriate prenatal screening in pregnant patients (%)						
**Region**	**Comparison Group**	**Model 1 Difference vs. Quintile 1 (95% CI)**	**Model 2 Difference vs. Quintile 1 (95% CI)**	**Model 3 Difference vs. Quintile 1 (95% CI)**	**Model 4 Difference vs. Quintile 1 (95% CI)**	**Model 5 Difference vs. Quintile 1 (95% CI)**
Southeast MSA 1	Quintile 2	-11.1 (-13.6 to -8.6)	-11.1 (-14.3 to -7.9)	-11.2 (-13.7 to -8.7)	-11.3 (-13.9 to -8.7)	-11.4 (-14.0 to -8.8)
	Quintile 3	-20.1 (-22.6 to -17.6)	-20.1 (-23.1 to -17.1)	-20.4 (-22.9 to -17.8)	-20.4 (-22.9 to -18.0)	-20.7 (-23.1 to -18.2)
	Quintile 4	-30.5 (-33.1 to -27.9)	-30.5 (-33.6 to -27.4)	-30.8 (-33.4 to -28.2)	-30.9 (-33.3 to -28.5)	-31.2 (-33.6 to -28.8)
	Quintile 5	-51.2 (-57.2 to -45.2)	-51.2 (-54.1 to -48.2)	-51.2 (-57.0 to -45.3)	-51.7 (-57.5 to -45.9)	-51.7 (-57.4 to -46.0)
Southeast MSA 2	Quintile 2	-9.6 (-11.6 to -7.7)	-9.6 (-12.4 to -6.8)	-9.7 (-11.7 to -7.7)	-9.5 (-11.5 to -7.5)	-9.5 (-11.5 to -7.5)
	Quintile 3	-15.8 (-17.6 to -14.0)	-15.8 (-18.9 to -12.7)	-15.8 (-17.6 to -14.0)	-15.8 (-17.7 to -14.0)	-15.9 (-17.7 to -14.0)
	Quintile 4	-23.4 (-25.5 to -21.3)	-23.4 (-26.5 to -20.3)	-23.5 (-25.6 to -21.4)	-23.1 (-25.2 to -20.9)	-23.2 (-25.3 to -21.0)
	Quintile 5	-33.8 (-37.8 to -29.9)	-33.8 (-36.9 to -30.8)	-34.0 (-38.0 to -30.1)	-34.2 (-38.2 to -30.2)	-34.4 (-38.4 to -30.4)
South Central MSA	Quintile 2	-7.6 (-8.5 to -6.8)	-7.6 (-8.6 to -6.7)	-7.7 (-8.7 to -6.8)	-7.5 (-8.4 to -6.6)	-7.6 (-8.6 to -6.6)
	Quintile 3	-13.7 (-14.5 to -12.8)	-13.7 (-14.6 to -12.7)	-13.7 (-14.6 to -12.7)	-13.4 (-14.4 to -12.4)	-13.4 (-14.4 to -12.4)
	Quintile 4	-21.4 (-22.5 to -20.3)	-21.4 (-22.5 to -20.3)	-21.3 (-22.4 to -20.2)	-21.0 (-22.1 to -19.9)	-20.9 (-22.0 to -19.7)
	Quintile 5	-37.7 (-40.2 to -35.2)	-37.7 (-38.8 to -36.5)	-37.6 (-40.2 to -35.1)	-37.2 (-39.8 to -34.6)	-37.2 (-39.8 to -34.6)
Midwest MSA	Quintile 2	-4.9 (-5.8 to -4.1)	-4.9 (-6.0 to -3.9)	-4.9 (-5.8 to -4.1)	-5.1 (-5.9 to -4.2)	-5.1 (-5.9 to -4.2)
	Quintile 3	-10.6 (-11.6 to -9.6)	-10.6 (-11.8 to -9.4)	-10.6 (-11.6 to -9.6)	-10.9 (-11.9 to -9.9)	-10.9 (-11.9 to -9.9)
	Quintile 4	-17.2 (-18.1 to -16.3)	-17.2 (-18.4 to -16.0)	-17.4 (-18.3 to -16.5)	-17.5 (-18.4 to -16.6)	-17.7 (-18.6 to -16.8)
	Quintile 5	-27.7 (-31.0 to -24.4)	-27.7 (-29.1 to -26.3)	-27.7 (-31.0 to -24.3)	-28.0 (-31.4 to -24.5)	-27.9 (-31.4 to -24.5)
West MSA	Quintile 2	-5.3 (-6.3 to -4.3)	-5.3 (-6.9 to -3.7)	-5.3 (-6.3 to -4.3)	-5.4 (-6.4 to -4.3)	-5.4 (-6.4 to -4.4)
	Quintile 3	-9.6 (-10.7 to -8.6)	-9.6 (-11.3 to -8.0)	-9.7 (-10.7 to -8.7)	-9.8 (-10.7 to -8.8)	-9.8 (-10.8 to -8.9)
	Quintile 4	-14.6 (-15.8 to -13.4)	-14.6 (-16.4 to -12.8)	-14.6 (-15.7 to -13.4)	-14.7 (-15.9 to -13.6)	-14.7 (-15.8 to -13.6)
	Quintile 5	-32.2 (-37.7 to -26.6)	-32.2 (-34.2 to -30.2)	-32.2 (-37.7 to -26.7)	-32.3 (-37.9 to -26.8)	-32.4 (-37.9 to -26.9)

© 2022 Song Z et al. *JAMA Health Forum.*

Caesarean delivery in patients with low-risk pregnancies (%)						
**Region**	**Comparison Group**	**Model 1 Difference vs. Quintile 1 (95% CI)**	**Model 2 Difference vs. Quintile 1 (95% CI)**	**Model 3 Difference vs. Quintile 1 (95% CI)**	**Model 4 Difference vs. Quintile 1 (95% CI)**	**Model 5 Difference vs. Quintile 1 (95% CI)**
Southeast MSA 1	Quintile 2	13.8 (10.6 to 17.1)	13.8 (11.5 to 16.1)	13.6 (10.3 to 16.9)	14.0 (10.7 to 17.2)	13.7 (10.5 to 17.0)
	Quintile 3	21.5 (18.3 to 24.7)	21.5 (19.1 to 23.9)	21.5 (18.2 to 24.7)	21.5 (18.3 to 24.7)	21.4 (18.2 to 24.7)
	Quintile 4	28.9 (25.6 to 32.2)	28.9 (26.4 to 31.3)	28.9 (25.6 to 32.2)	28.9 (25.7 to 32.2)	29.0 (25.7 to 32.2)
	Quintile 5	45.5 (40.2 to 50.8)	45.5 (43.1 to 47.9)	45.8 (40.5 to 51.1)	45.5 (40.2 to 50.8)	45.8 (40.5 to 51.1)
Southeast MSA 2	Quintile 2	10.3 (7.6 to 13.1)	10.3 (8.1 to 12.6)	10.4 (7.6 to 13.2)	10.4 (7.7 to 13.1)	10.5 (7.7 to 13.2)
	Quintile 3	16.9 (14.2 to 19.6)	16.9 (14.6 to 19.2)	17.0 (14.2 to 19.7)	16.9 (14.2 to 19.6)	16.9 (14.2 to 19.7)
	Quintile 4	23.2 (20.4 to 26.0)	23.2 (20.9 to 25.6)	23.0 (20.2 to 25.9)	23.2 (20.5 to 26.0)	23.0 (20.3 to 25.8)
	Quintile 5	36.6 (32.3 to 40.9)	36.6 (34.2 to 39.0)	36.7 (32.3 to 41.0)	36.5 (32.2 to 40.7)	36.5 (32.2 to 40.8)
South Central MSA	Quintile 2	10.3 (9.0 to 11.6)	10.3 (9.3 to 11.3)	10.2 (8.9 to 11.5)	10.2 (8.9 to 11.5)	10.1 (8.8 to 11.4)
	Quintile 3	17.0 (15.7 to 18.2)	17.0 (16.0 to 18.0)	16.7 (15.4 to 18.0)	16.8 (15.5 to 18.0)	16.5 (15.3 to 17.8)
	Quintile 4	24.1 (22.8 to 25.4)	24.1 (23.1 to 25.1)	23.8 (22.5 to 25.2)	23.8 (22.5 to 25.1)	23.5 (22.2 to 24.9)
	Quintile 5	43.3 (39.7 to 46.9)	43.3 (42.2 to 44.4)	43.1 (39.5 to 46.8)	42.8 (39.2 to 46.4)	42.6 (39.0 to 46.3)
Midwest MSA	Quintile 2	11.1 (9.9 to 12.2)	11.1 (10.2 to 11.9)	11.0 (9.8 to 12.1)	11.0 (9.9 to 12.1)	10.9 (9.8 to 12.1)
	Quintile 3	18.0 (16.9 to 19.2)	18.0 (17.1 to 19.0)	17.9 (16.7 to 19.0)	18.0 (16.9 to 19.1)	17.8 (16.7 to 19.0)
	Quintile 4	24.5 (23.4 to 25.7)	24.5 (23.6 to 25.5)	24.5 (23.3 to 25.7)	24.6 (23.4 to 25.8)	24.5 (23.3 to 25.7)
	Quintile 5	40.6 (37.9 to 43.3)	40.6 (39.6 to 41.7)	40.6 (37.9 to 43.3)	40.6 (37.9 to 43.3)	40.6 (37.9 to 43.2)
West MSA	Quintile 2	13.7 (11.9 to 15.4)	13.7 (12.2 to 15.2)	13.8 (12.0 to 15.6)	13.9 (12.2 to 15.7)	14.0 (12.2 to 15.8)
	Quintile 3	21.6 (20.0 to 23.3)	21.6 (20.0 to 23.3)	21.6 (19.9 to 23.3)	21.7 (20.0 to 23.3)	21.6 (19.9 to 23.3)
	Quintile 4	30.1 (28.4 to 31.8)	30.1 (28.3 to 31.8)	30.2 (28.4 to 31.9)	30.4 (28.7 to 32.2)	30.5 (28.7 to 32.2)
	Quintile 5	51.7 (45.8 to 57.6)	51.7 (49.8 to 53.5)	51.7 (45.7 to 57.6)	51.7 (45.7 to 57.7)	51.8 (45.7 to 57.8)

* Differences between quintile 1 (reference) and quintiles 2-5 are derived from a model adjusted for risk score (age, sex, and diagnoses) and socioeconomic variables, with standard errors clustered by physician. 95% confidence intervals are shown in parentheses. Model 1 results are the main estimates. Sensitivity analyses tested the robustness of the main estimates to alterations in the model, focusing on the role of

© 2022 Song Z et al. *JAMA Health Forum.*

clinical risk and socioeconomic status adjustment. Model 2 omitted clustered standard errors. Model 3 omitted the DxCG risk score. Model 4 omitted the SES score. Model 5 omitted both the DxCG and SES scores. Stable estimates among these sensitivity analyses relative to Model 1 would suggest that any observable differences in patient age, sex, clinical diagnoses, and socioeconomic status characteristics across physician quintiles contributed minimal bias toward the differences in performance between the quintiles. However, they would not adjust for all potential confounders.

.

© 2022 Song Z et al. *JAMA Health Forum.*

eTable 6. Physician-Level Variation in Orthopedist Joint Care*

Any physical therapy (PT) prior to elective hip or knee replacement (%)						
**Region**	**Comparison Group**	**Model 1 Difference vs. Quintile 1 (95% CI)**	**Model 2 Difference vs. Quintile 1 (95% CI)**	**Model 3 Difference vs. Quintile 1 (95% CI)**	**Model 4 Difference vs. Quintile 1 (95% CI)**	**Model 5 Difference vs. Quintile 1 (95% CI)**
Southeast MSA 1	Quintile 2	-5.9 (-8.7 to -3.0)	-5.9 (-10.1 to -1.6)	-5.8 (-8.8 to -2.9)	-5.8 (-8.3 to -3.3)	-5.7 (-8.2 to -3.2)
	Quintile 3	-10.2 (-12.8 to -7.6)	-10.2 (-14.1 to -6.3)	-10.3 (-12.9 to -7.7)	-9.7 (-12.0 to -7.3)	-9.8 (-12.1 to -7.4)
	Quintile 4	-13.2 (-16.2 to -10.2)	-13.2 (-16.7 to -9.7)	-13.1 (-16.1 to -10.1)	-12.3 (-15.2 to -9.4)	-12.2 (-15.2 to -9.2)
	Quintile 5	-15.9 (-18.7 to -13.0)	-15.9 (-19.1 to -12.6)	-15.9 (-18.8 to -13.0)	-15.2 (-17.7 to -12.7)	-15.3 (-17.8 to -12.7)
Southeast MSA 2	Quintile 2	-27.3 (-37.1 to -17.6)	-27.3 (-31.7 to -23.0)	-27.3 (-37.0 to -17.6)	-27.3 (-36.7 to -17.9)	-27.2 (-36.6 to -17.9)
	Quintile 3	-35.1 (-44.6 to -25.7)	-35.1 (-38.9 to -31.3)	-35.1 (-44.6 to -25.7)	-35.2 (-44.3 to -26.1)	-35.2 (-44.2 to -26.1)
	Quintile 4	-38.4 (-47.8 to -29.0)	-38.4 (-42.1 to -34.7)	-38.4 (-47.8 to -29.0)	-38.3 (-47.3 to -29.3)	-38.3 (-47.3 to -29.3)
	Quintile 5	-43.7 (-53.3 to -34.2)	-43.7 (-47.7 to -39.7)	-43.6 (-53.2 to -34.0)	-43.9 (-52.9 to -34.8)	-43.8 (-52.8 to -34.7)
South Central MSA	Quintile 2	-20.2 (-28.5 to -12.0)	-20.2 (-22.7 to -17.8)	-20.1 (-28.3 to -12.0)	-20.6 (-29.2 to -12.0)	-20.6 (-29.1 to -12.1)
	Quintile 3	-26.5 (-34.7 to -18.3)	-26.5 (-28.9 to -24.1)	-26.1 (-34.2 to -18.1)	-27.0 (-35.6 to -18.4)	-26.7 (-35.2 to -18.2)
	Quintile 4	-31.5 (-39.7 to -23.3)	-31.5 (-33.7 to -29.3)	-31.4 (-39.5 to -23.4)	-32.3 (-40.9 to -23.7)	-32.2 (-40.7 to -23.7)
	Quintile 5	-36.5 (-44.6 to -28.4)	-36.5 (-38.9 to -34.1)	-36.5 (-44.5 to -28.4)	-36.9 (-45.5 to -28.3)	-37.0 (-45.5 to -28.4)
Midwest MSA	Quintile 2	-27.4 (-33.6 to -21.1)	-27.4 (-29.3 to -25.4)	-27.3 (-33.5 to -21.1)	-27.1 (-33.3 to -20.8)	-27.0 (-33.2 to -20.8)
	Quintile 3	-32.9 (-39.1 to -26.7)	-32.9 (-34.9 to -30.9)	-32.8 (-39.0 to -26.7)	-33.3 (-39.4 to -27.1)	-33.2 (-39.4 to -27.0)
	Quintile 4	-38.9 (-45.1 to -32.7)	-38.9 (-41.0 to -36.7)	-38.9 (-45.1 to -32.7)	-39.1 (-45.3 to -32.8)	-39.1 (-45.3 to -32.9)
	Quintile 5	-45.5 (-51.8 to -39.3)	-45.5 (-47.9 to -43.2)	-45.4 (-51.6 to -39.2)	-45.9 (-52.1 to -39.6)	-45.7 (-52.0 to -39.5)
West MSA	Quintile 2	-22.3 (-29.1 to -15.5)	-22.3 (-26.1 to -18.5)	-22.3 (-29.1 to -15.5)	-22.7 (-29.4 to -16.0)	-22.7 (-29.5 to -15.9)
	Quintile 3	-33.8 (-40.1 to -27.6)	-33.8 (-37.3 to -30.4)	-34.2 (-40.4 to -28.0)	-34.0 (-40.2 to -27.8)	-34.4 (-40.6 to -28.2)
	Quintile 4	-41.3 (-47.6 to -35.1)	-41.3 (-45.2 to -37.4)	-41.6 (-47.8 to -35.4)	-40.8 (-46.9 to -34.7)	-41.0 (-47.1 to -34.9)
	Quintile 5	-49.1 (-56.4 to -41.9)	-49.1 (-53.2 to -45.1)	-49.2 (-56.3 to -42.1)	-48.2 (-54.8 to -41.7)	-48.2 (-54.6 to -41.7)

© 2022 Song Z et al. *JAMA Health Forum.*

Arthroscopy in patients with new hip or knee osteoarthritis (%)						
**Region**	**Comparison Group**	**Model 1 Difference vs. Quintile 1 (95% CI)**	**Model 2 Difference vs. Quintile 1 (95% CI)**	**Model 3 Difference vs. Quintile 1 (95% CI)**	**Model 4 Difference vs. Quintile 1 (95% CI)**	**Model 5 Difference vs. Quintile 1 (95% CI)**
Southeast MSA 1	Quintile 2	4.4 (3.1 to 5.7)	4.4 (2.4 to 6.4)	4.4 (3.1 to 5.7)	4.3 (3.0 to 5.7)	4.3 (3.0 to 5.6)
	Quintile 3	10.3 (9.0 to 11.6)	10.3 (7.9 to 12.7)	10.3 (9.0 to 11.5)	9.9 (8.5 to 11.3)	9.9 (8.5 to 11.3)
	Quintile 4	17.4 (16.2 to 18.6)	17.4 (14.6 to 20.1)	17.4 (16.2 to 18.6)	17.1 (15.8 to 18.4)	17.1 (15.8 to 18.4)
	Quintile 5	27.9 (25.5 to 30.2)	27.9 (24.8 to 31.0)	27.9 (25.5 to 30.3)	27.5 (25.0 to 30.0)	27.5 (25.0 to 30.0)
Southeast MSA 2	Quintile 2	4.2 (3.2 to 5.1)	4.2 (2.8 to 5.6)	4.2 (3.2 to 5.1)	4.1 (3.1 to 5.2)	4.1 (3.1 to 5.2)
	Quintile 3	8.6 (7.8 to 9.5)	8.6 (6.9 to 10.4)	8.6 (7.7 to 9.4)	8.3 (7.5 to 9.2)	8.3 (7.4 to 9.2)
	Quintile 4	14.7 (13.5 to 15.8)	14.7 (12.7 to 16.6)	14.6 (13.5 to 15.8)	14.7 (13.5 to 15.9)	14.6 (13.4 to 15.8)
	Quintile 5	25.0 (22.1 to 27.8)	25.0 (22.7 to 27.2)	25.0 (22.1 to 27.9)	25.2 (22.2 to 28.2)	25.2 (22.2 to 28.3)
South Central MSA	Quintile 2	4.0 (3.3 to 4.7)	4.0 (3.2 to 4.8)	4.0 (3.2 to 4.7)	4.1 (3.3 to 4.8)	4.0 (3.3 to 4.8)
	Quintile 3	9.5 (8.9 to 10.2)	9.5 (8.6 to 10.5)	9.5 (8.8 to 10.3)	9.5 (8.8 to 10.1)	9.4 (8.7 to 10.1)
	Quintile 4	15.9 (15.0 to 16.8)	15.9 (14.8 to 17.0)	15.8 (15.0 to 16.7)	15.7 (14.8 to 16.7)	15.6 (14.7 to 16.6)
	Quintile 5	28.1 (26.3 to 29.9)	28.1 (26.8 to 29.4)	28.0 (26.3 to 29.8)	28.1 (26.3 to 29.9)	28.0 (26.2 to 29.8)
Midwest MSA	Quintile 2	3.6 (3.1 to 4.2)	3.6 (2.9 to 4.3)	3.7 (3.1 to 4.3)	3.6 (3.0 to 4.2)	3.7 (3.1 to 4.3)
	Quintile 3	7.3 (6.7 to 7.9)	7.3 (6.4 to 8.1)	7.4 (6.8 to 8.0)	7.3 (6.7 to 7.9)	7.4 (6.8 to 8.0)
	Quintile 4	12.6 (11.9 to 13.2)	12.6 (11.6 to 13.5)	12.7 (12.1 to 13.4)	12.6 (11.9 to 13.2)	12.7 (12.1 to 13.4)
	Quintile 5	23.0 (21.7 to 24.3)	23.0 (21.9 to 24.2)	23.0 (21.7 to 24.3)	23.0 (21.7 to 24.3)	23.0 (21.7 to 24.3)
West MSA	Quintile 2	4.5 (3.5 to 5.5)	4.5 (2.8 to 6.2)	4.4 (3.4 to 5.4)	4.5 (3.4 to 5.6)	4.4 (3.4 to 5.5)
	Quintile 3	8.8 (7.8 to 9.7)	8.8 (6.9 to 10.6)	8.7 (7.8 to 9.7)	8.6 (7.6 to 9.6)	8.6 (7.6 to 9.6)
	Quintile 4	13.9 (12.8 to 14.9)	13.9 (11.8 to 15.9)	14.0 (12.9 to 15.1)	13.7 (12.7 to 14.7)	13.8 (12.7 to 14.8)
	Quintile 5	22.9 (20.0 to 25.9)	22.9 (20.6 to 25.3)	23.1 (20.1 to 26.0)	23.2 (20.2 to 26.2)	23.3 (20.4 to 26.3)

* Differences between quintile 1 (reference) and quintiles 2-5 are derived from a model adjusted for risk score (age, sex, and diagnoses) and socioeconomic variables, with standard errors clustered by physician. 95% confidence intervals are shown in parentheses. Model 1 results are the main estimates. Sensitivity analyses tested the robustness of the main estimates to alterations in the model, focusing on the role of

© 2022 Song Z et al. *JAMA Health Forum.*

clinical risk and socioeconomic status adjustment. Model 2 omitted clustered standard errors. Model 3 omitted the DxCG risk score. Model 4 omitted the SES score. Model 5 omitted both the DxCG and SES scores. Stable estimates among these sensitivity analyses relative to Model 1 would suggest that any observable differences in patient age, sex, clinical diagnoses, and socioeconomic status characteristics across physician quintiles contributed minimal bias toward the differences in performance between the quintiles. However, they would not adjust for all potential confounders.

© 2022 Song Z et al. *JAMA Health Forum.*

eTable 7. Physician-Level Variation in Orthopedist/Neurosurgeon Spine Care*

Spinal fusion in patients with low back pain (%)						
**Region**	**Comparison Group**	**Model 1 Difference vs. Quintile 1 (95% CI)**	**Model 2 Difference vs. Quintile 1 (95% CI)**	**Model 3 Difference vs. Quintile 1 (95% CI)**	**Model 4 Difference vs. Quintile 1 (95% CI)**	**Model 5 Difference vs. Quintile 1 (95% CI)**
Southeast MSA 1	Quintile 2	14.1 (8.5 to 19.8)	14.1 (8.3 to 20.0)	14.1 (8.6 to 19.6)	14.6 (8.9 to 20.3)	14.6 (9.1 to 20.2)
	Quintile 3	23.3 (18.0 to 28.6)	23.3 (17.8 to 28.8)	23.2 (18.1 to 28.4)	24.1 (19.0 to 29.3)	24.1 (19.2 to 29.0)
	Quintile 4	36.6 (30.9 to 42.3)	36.6 (31.5 to 41.7)	36.4 (31.0 to 41.8)	37.8 (31.6 to 44.1)	37.7 (31.9 to 43.6)
	Quintile 5	57.5 (49.3 to 65.7)	57.5 (52.5 to 62.5)	58.0 (49.8 to 66.1)	57.5 (49.2 to 65.9)	58.1 (49.8 to 66.4)
Southeast MSA 2	Quintile 2	18.1 (14.5 to 21.7)	18.1 (14.1 to 22.1)	18.3 (14.7 to 22.0)	17.9 (14.3 to 21.6)	18.2 (14.5 to 21.8)
	Quintile 3	28.9 (25.3 to 32.6)	28.9 (24.9 to 33.0)	28.9 (25.4 to 32.4)	28.7 (24.9 to 32.5)	28.7 (25.1 to 32.3)
	Quintile 4	38.7 (34.4 to 43.0)	38.7 (34.2 to 43.2)	38.8 (34.4 to 43.1)	38.5 (34.1 to 42.8)	38.5 (34.1 to 42.9)
	Quintile 5	58.8 (50.1 to 67.5)	58.8 (54.4 to 63.1)	59.1 (50.4 to 67.8)	58.1 (49.1 to 67.1)	58.4 (49.5 to 67.4)
South Central MSA	Quintile 2	11.6 (8.7 to 14.5)	11.6 (9.3 to 13.9)	12.1 (9.0 to 15.1)	11.4 (8.5 to 14.3)	11.9 (9.0 to 14.7)
	Quintile 3	19.8 (16.8 to 22.7)	19.8 (17.4 to 22.1)	21.1 (18.1 to 24.2)	20.4 (17.5 to 23.2)	21.8 (18.9 to 24.7)
	Quintile 4	32.7 (29.4 to 36.0)	32.7 (30.3 to 35.1)	34.2 (30.9 to 37.5)	32.5 (29.2 to 35.8)	34.1 (30.8 to 37.4)
	Quintile 5	55.4 (48.8 to 61.9)	55.4 (53.2 to 57.6)	56.6 (50.3 to 62.9)	55.5 (49.1 to 61.9)	56.7 (50.6 to 62.9)
Midwest MSA	Quintile 2	13.9 (10.7 to 17.1)	13.9 (11.2 to 16.6)	13.7 (10.1 to 17.2)	13.9 (10.4 to 17.4)	13.7 (9.8 to 17.6)
	Quintile 3	23.4 (20.5 to 26.3)	23.4 (20.6 to 26.2)	23.5 (20.3 to 26.8)	23.5 (20.4 to 26.6)	23.7 (20.1 to 27.2)
	Quintile 4	30.8 (27.8 to 33.7)	30.8 (27.9 to 33.6)	30.4 (27.2 to 33.7)	31.2 (28.1 to 34.3)	30.9 (27.3 to 34.4)
	Quintile 5	45.0 (40.6 to 49.4)	45.0 (42.1 to 47.9)	45.8 (41.2 to 50.4)	45.1 (40.7 to 49.6)	45.9 (41.3 to 50.6)
West MSA	Quintile 2	16.3 (12.7 to 20.0)	16.3 (11.8 to 20.9)	16.0 (12.1 to 19.9)	16.2 (12.8 to 19.7)	15.9 (12.0 to 19.8)
	Quintile 3	24.4 (21.5 to 27.3)	24.4 (19.6 to 29.1)	24.8 (21.3 to 28.3)	23.5 (20.2 to 26.8)	24.0 (20.3 to 27.8)
	Quintile 4	39.7 (35.4 to 44.0)	39.7 (34.5 to 44.9)	41.0 (36.3 to 45.6)	39.5 (35.1 to 43.9)	40.7 (36.1 to 45.4)
	Quintile 5	63.0 (58.2 to 67.8)	63.0 (58.1 to 67.9)	62.6 (57.7 to 67.5)	62.7 (58.4 to 67.0)	62.3 (57.9 to 66.7)

© 2022 Song Z et al. *JAMA Health Forum.*

Any physical therapy (PT) in patients with new cervical spine pain (%)						
**Region**	**Comparison Group**	**Model 1 Difference vs. Quintile 1 (95% CI)**	**Model 2 Difference vs. Quintile 1 (95% CI)**	**Model 3 Difference vs. Quintile 1 (95% CI)**	**Model 4 Difference vs. Quintile 1 (95% CI)**	**Model 5 Difference vs. Quintile 1 (95% CI)**
Southeast MSA 1	Quintile 2	-22.5 (-30.0 to -15.1)	-22.5 (-29.6 to -15.5)	-22.5 (-29.9 to -15.1)	-23.0 (-30.5 to -15.5)	-23.0 (-30.4 to -15.5)
	Quintile 3	-32.4 (-39.8 to -25.0)	-32.4 (-39.7 to -25.1)	-31.5 (-39.1 to -24.0)	-33.3 (-40.6 to -25.9)	-32.4 (-39.8 to -25.0)
	Quintile 4	-40.1 (-47.5 to -32.8)	-40.1 (-46.2 to -34.1)	-39.9 (-47.2 to -32.6)	-40.5 (-47.8 to -33.1)	-40.3 (-47.5 to -33.1)
	Quintile 5	-48.1 (-55.5 to -40.7)	-48.1 (-53.4 to -42.7)	-47.6 (-55.1 to -40.2)	-48.0 (-55.3 to -40.7)	-47.6 (-54.9 to -40.3)
Southeast MSA 2	Quintile 2	-13.3 (-18.7 to -7.8)	-13.3 (-19.4 to -7.1)	-13.3 (-18.8 to -7.8)	-13.9 (-19.3 to -8.6)	-13.9 (-19.3 to -8.5)
	Quintile 3	-20.3 (-25.7 to -14.9)	-20.3 (-26.3 to -14.3)	-20.3 (-25.7 to -14.9)	-21.3 (-26.5 to -16.0)	-21.2 (-26.5 to -16.0)
	Quintile 4	-30.3 (-35.9 to -24.7)	-30.3 (-36.0 to -24.5)	-30.3 (-35.8 to -24.7)	-31.1 (-36.5 to -25.7)	-31.1 (-36.5 to -25.7)
	Quintile 5	-39.9 (-46.2 to -33.7)	-39.9 (-44.8 to -35.1)	-39.9 (-46.1 to -33.7)	-41.0 (-47.2 to -34.7)	-41.0 (-47.2 to -34.7)
South Central MSA	Quintile 2	-17.3 (-21.5 to -13.2)	-17.3 (-20.1 to -14.5)	-17.6 (-21.8 to -13.5)	-16.8 (-20.8 to -12.7)	-17.1 (-21.1 to -13.1)
	Quintile 3	-27.9 (-31.9 to -23.9)	-27.9 (-30.6 to -25.2)	-28.0 (-32.0 to -24.0)	-27.9 (-32.0 to -23.9)	-28.0 (-32.0 to -24.0)
	Quintile 4	-34.5 (-38.6 to -30.4)	-34.5 (-37.2 to -31.9)	-34.7 (-38.7 to -30.6)	-34.8 (-38.8 to -30.9)	-35.0 (-38.9 to -31.1)
	Quintile 5	-46.1 (-50.5 to -41.7)	-46.1 (-48.6 to -43.5)	-46.1 (-50.5 to -41.8)	-46.1 (-50.4 to -41.8)	-46.1 (-50.4 to -41.8)
Midwest MSA	Quintile 2	-10.6 (-13.1 to -8.1)	-10.6 (-13.3 to -7.9)	-10.7 (-13.2 to -8.2)	-10.5 (-13.1 to -8.0)	-10.6 (-13.2 to -8.1)
	Quintile 3	-17.1 (-19.5 to -14.7)	-17.1 (-19.8 to -14.4)	-17.2 (-19.6 to -14.7)	-17.1 (-19.6 to -14.7)	-17.3 (-19.7 to -14.8)
	Quintile 4	-24.2 (-26.7 to -21.8)	-24.2 (-27.0 to -21.5)	-24.5 (-27.0 to -22.0)	-24.2 (-26.7 to -21.6)	-24.4 (-26.9 to -21.9)
	Quintile 5	-36.0 (-39.1 to -32.9)	-36.0 (-38.6 to -33.4)	-35.6 (-38.7 to -32.5)	-36.2 (-39.5 to -33.0)	-35.9 (-39.1 to -32.7)
West MSA	Quintile 2	-9.4 (-13.8 to -5.0)	-9.4 (-17.4 to -1.4)	-9.1 (-13.2 to -5.1)	-10.5 (-15.5 to -5.6)	-10.1 (-14.6 to -5.6)
	Quintile 3	-17.7 (-21.7 to -13.8)	-17.7 (-25.1 to -10.4)	-17.2 (-21.0 to -13.4)	-18.0 (-22.4 to -13.6)	-17.4 (-21.5 to -13.4)
	Quintile 4	-24.2 (-28.5 to -19.9)	-24.2 (-31.9 to -16.5)	-22.9 (-27.3 to -18.5)	-25.6 (-30.7 to -20.5)	-24.2 (-29.5 to -19.0)
	Quintile 5	-37.1 (-44.1 to -30.1)	-37.1 (-43.8 to -30.4)	-36.5 (-43.5 to -29.5)	-39.3 (-46.6 to -32.0)	-38.7 (-45.9 to -31.5)

* Differences between quintile 1 (reference) and quintiles 2-5 are derived from a model adjusted for risk score (age, sex, and diagnoses) and socioeconomic variables, with standard errors clustered by physician. 95% confidence intervals are shown in parentheses. Model 1 results are the main estimates. Sensitivity analyses tested the robustness of the main estimates to alterations in the model, focusing on the role of

© 2022 Song Z et al. *JAMA Health Forum.*

clinical risk and socioeconomic status adjustment. Model 2 omitted clustered standard errors. Model 3 omitted the DxCG risk score. Model 4 omitted the SES score. Model 5 omitted both the DxCG and SES scores. Stable estimates among these sensitivity analyses relative to Model 1 would suggest that any observable differences in patient age, sex, clinical diagnoses, and socioeconomic status characteristics across physician quintiles contributed minimal bias toward the differences in performance between the quintiles. However, they would not adjust for all potential confounders.

© 2022 Song Z et al. *JAMA Health Forum.*

eTable 8. Physician-Level Variations in Health Care Spending within Domains of Care

	Quintile 1	Quintile 2		Quintile 3		Quintile 4		Quintile 5	
	Mean (Reference)	Mean	Difference (95% CI)	Mean	Difference (95% CI)	Mean	Difference (95% CI)	Mean	Difference (95% CI)
**Cardiologists**									
Spending on CAD-related care ($ per patient per year)[1]									
Southeast MSA 1	3,807	4,358	714	5,047	1,385	5,627	2,207	7,092	3,807
			(511 to 916)		(1,198 to 1,572)		(1,991 to 2,424)		(3,439 to 4,175)
Southeast MSA 2	3,589	4,011	573	4,845	1,265	5,373	2,093	7,594	4,452
			(411 to 735)		(1,112 to 1,418)		(1,917 to 2,268)		(3,748 to 5,157)
South Central MSA	3,636	4,445	1,176	5,105	1,894	5,930	2,849	7,592	4,797
			(977 to 1,374)		(1,709 to 2,079)		(2,643 to 3,055)		(4,407 to 5,186)
Midwest MSA	3,561	4,340	839	4,871	1,401	5,521	2,001	6,968	3,481
			(697 to 981)		(1,268 to 1,535)		(1,858 to 2,144)		(3,185 to 3,777)
West MSA	3,091	3,517	508	4,197	1,104	4,730	1,892	5,657	2,914
			(215 to 801)		(855 to 1,352)		(1,612 to 2,173)		(2,453 to 3,374)
**Endocrinologists**									
Spending on diabetes-related care ($ per patient per year)[2]									
Southeast MSA 1	2,078	2,422	383	2,654	708	3,058	940	3,518	1,523
			(261 to 506)		(578 to 837)		(801 to 1,079)		(1,249 to 1,796)
Southeast MSA 2	2,083	2,449	362	2,757	624	2,958	841	3,453	1,382
			(235 to 490)		(492 to 755)		(707 to 974)		(1,167 to 1,597)
South Central MSA	2,098	2,525	410	2,857	802	3,326	1,168	4,188	1,986
			(225 to 596)		(626 to 978)		(961 to 1,375)		(1,704 to 2,268)
Midwest MSA	2,182	2,520	412	2,724	661	2,942	859	3,665	1,575
			(316 to 507)		(574 to 747)		(770 to 949)		(1,287 to 1,864)
West MSA	2,150	2,500	428	2,776	664	3,067	1,011	3,884	1,718
			(322 to 534)		(527 to 801)		(900 to 1,121)		(1,240 to 2,197)
**Gastroenterologists**									
Spending per colonoscopy episode ($)[3]									
Southeast MSA 1	1,086	1,233	149	1,411	330	1,641	556	2,070	981
			(119 to 180)		(279 to 380)		(508 to 605)		(887 to 1,075)
Southeast MSA 2	1,145	1,313	169	1,444	294	1,611	462	2,163	1,000
			(129 to 210)		(257 to 330)		(420 to 503)		(879 to 1,121)
South Central MSA	1,393	1,520	123	1,637	235	1,780	374	2,233	821
			(102 to 143)		(214 to 256)		(351 to 396)		(611 to 1,032)
Midwest MSA	1,224	1,434	214	1,574	352	1,754	520	2,257	1,000

© 2022 Song Z et al. *JAMA Health Forum.*

	Quintile 1	Quintile 2		Quintile 3		Quintile 4		Quintile 5	
	Mean (Reference)	Mean	Difference (95% CI)	Mean	Difference (95% CI)	Mean	Difference (95% CI)	Mean	Difference (95% CI)
			(179 to 249)		(316 to 388)		(484 to 556)		(854 to 1,146)
West MSA	570	673	113	1,087	516	1,397	821	1,743	1,163
			(96 to 131)		(463 to 569)		(797 to 844)		(1,063 to 1,262)
**Pulmonologists**									
Spending on COPD-related care ($ per patient per year)[4]									
Southeast MSA 1	1,752	1,995	280	2,628	792	2,849	1,355	3,729	2,398
			(-36 to 595)		(490 to 1,095)		(1,046 to 1,664)		(1,897 to 2,899)
Southeast MSA 2	1,652	2,040	508	2,351	851	2,856	1,345	3,562	2,360
			(243 to 772)		(679 to 1,023)		(1,140 to 1,549)		(1,969 to 2,751)
South Central MSA	1,494	2,039	496	2,483	1,007	3,148	1,744	4,785	3,632
			(338 to 653)		(828 to 1,186)		(1,554 to 1,934)		(3,151 to 4,112)
Midwest MSA	1,924	2,221	645	2,707	1,242	3,546	2,009	4,361	3,138
			(428 to 863)		(1,062 to 1,423)		(1,809 to 2,209)		(2,743 to 3,533)
West MSA	1,485	1,517	387	2,071	700	2,170	1,109	2,766	1,963
			(208 to 567)		(569 to 832)		(960 to 1,258)		(1,499 to 2,428)
**Obstetricians**									
Spending per episode of low-risk pregnancy ($)[5]									
Southeast MSA 1	12,120	14,454	2,313	15,828	3,657	18,094	5,930	22,237	10,250
			(1,913 to 2,713)		(3,331 to 3,983)		(5,420 to 6,439)		(9,699 to 10,801)
Southeast MSA 2	12,777	15,934	3,131	17,866	5,078	19,924	7,250	21,776	9,086
			(2,589 to 3,672)		(4,567 to 5,590)		(6,726 to 7,773)		(8,490 to 9,682)
South Central MSA	12,949	14,526	1,546	15,478	2,513	16,506	3,558	18,377	5,436
			(1,314 to 1,778)		(2,279 to 2,747)		(3,317 to 3,799)		(5,096 to 5,776)
Midwest MSA	10,925	13,290	2,375	14,639	3,729	15,618	4,722	17,452	6,543
			(2,133 to 2,617)		(3,505 to 3,953)		(4,497 to 4,947)		(6,216 to 6,869)
West MSA	15,793	17,073	1,292	18,394	2,640	20,212	4,492	22,917	7,056
			(1,046 to 1,538)		(2,375 to 2,905)		(4,206 to 4,778)		(6,558 to 7,554)
**Orthopedic Surgeons (Joint)**									
Spending per episode of hip or knee replacement ($)[6]									
Southeast MSA 1	1,895	2,965	1,043	4,415	2,437	6,172	4,149	9,190	7,629
			(654 to 1,433)		(2,102 to 2,772)		(3,713 to 4,585)		(6,377 to 8,880)
Southeast MSA 2	1,469	2,678	1,195	4,072	2,664	6,088	4,712	10,505	9,074
			(866 to 1,523)		(2,400 to 2,928)		(4,358 to 5,066)		(7,999 to 10,148)
South Central MSA	1,746	2,628	906	3,841	2,088	5,868	4,035	11,208	9,175
			(730 to 1,082)		(1,888 to 2,289)		(3,748 to 4,321)		(8,250 to 10,099)

© 2022 Song Z et al. *JAMA Health Forum.*

	Quintile 1	Quintile 2		Quintile 3		Quintile 4		Quintile 5	
	Mean (Reference)	Mean	Difference (95% CI)	Mean	Difference (95% CI)	Mean	Difference (95% CI)	Mean	Difference (95% CI)
Midwest MSA	2,250	3,155	965	4,286	1,987	5,808	3,495	11,634	9,187
			(820 to 1,109)		(1,830 to 2,143)		(3,277 to 3,713)		(7,474 to 10,901)
West MSA	1,883	2,780	1,014	4,511	2,486	6,952	5,029	13,373	11,633
			(712 to 1,316)		(2,125 to 2,846)		(4,575 to 5,482)		(10,194 to 13,072)
**Orthopedic Surgeons and Neurosurgeons (Spine)**									
Spending on low back pain care ($ per patient per year)[7]									
Southeast MSA 1	2,150	2,979	880	4,525	2,697	7,506	5,415	12,869	10,954
			(-23 to 1,784)		(1,732 to 3,663)		(4,490 to 6,341)		(8,810 to 13,098)
Southeast MSA 2	2,666	3,703	801	4,833	2,077	6,659	4,097	10,483	7,912
			(-280 to 1,883)		(1,342 to 2,811)		(3,071 to 5,123)		(6,864 to 8,960)
South Central MSA	3,359	4,072	824	7,290	3,930	10,088	6,772	15,964	12,949
			(-57 to 1,704)		(3,305 to 4,556)		(6,079 to 7,466)		(11,427 to 14,472)
Midwest MSA	2,470	2,972	609	3,979	1,663	6,074	3,695	10,207	7,857
			(73 to 1,145)		(1,214 to 2,112)		(3,188 to 4,202)		(6,735 to 8,979)
West MSA	3,199	5,651	2,297	7,282	4,595	10,042	7,299	16,656	14,438
			(928 to 3,666)		(3,313 to 5,876)		(6,646 to 7,951)		(12,186 to 16,691)

Values shown are unadjusted averages of performance by quintile of physicians. Differences between quintile 1 (reference) and quintiles 2-5, respectively, are derived from a statistical model adjusted for risk score (age, sex, and diagnoses) and socioeconomic variables, with standard errors clustered by physician.

1. CAD-related is defined as cardiovascular inpatient admissions, and all claims with CAD, heart failure, diabetes, hyperlipidemia, obesity, or hypertension. Also included are drug fills for statins, beta blocker, ACE inhibitor, ARB, diuretic, anti-platelets, and oral diabetes agents.

2. Diabetes-related care includes all claims with a diabetes diagnosis listed as the principal diagnosis.

3. A colonoscopy "episode" spans 3 days prior through 14 days after the colonoscopy.

4. COPD-related care includes all claims with a COPD/bronchiectasis diagnosis listed as the principal diagnosis.

5. The definition of low-risk pregnancies in this study is provided in the footnote to eFigure 5.

6. An episode of hip or knee replacement is defined as one year after surgery, and spending on all non-pharmacy claims with a principal diagnosis of joint pain over the following year is included.

7. The spending measure refers to medical spending on all non-pharmacy claims with a principal diagnosis of low back pain one year following their initial diagnosis.

© 2022 Song Z et al. *JAMA Health Forum.*
